# A Systematic Review and Meta-Analysis of the Use of the National Institutes of Health Toolbox Cognition Battery in Clinical Populations

**DOI:** 10.1007/s11065-025-09669-3

**Published:** 2025-07-07

**Authors:** Kelly H. Watson, Abagail E. Ciriegio, Claire F. Miller, Marissa C. Roth, Bruce E. Compas

**Affiliations:** 1 Department of Neurology, Vanderbilt University Medical Center, Nashville, TN 37232 USA; 2 Department of Psychology & Human Development, Vanderbilt University, Nashville, TN 37203 USA; 3 Department of Pediatrics, University of Utah Health, Salt Lake City, UT 84132 USA

**Keywords:** NIH Toolbox, Cognition, Fluid, Crystallized, Assessment, Meta-analysis

## Abstract

The National Institutes of Health Toolbox Cognition Battery (NIHTB-CB) is an assessment tool that has been widely utilized in research with clinical populations across the lifespan. Despite its widespread use, a systematic review and meta-analysis of cognitive function utilizing this battery in clinical samples has not been reported. To address this gap, 84 studies were identified after systematically searching PsycINFO, PubMed, and ProQuest (71 peer-reviewed articles, 11 dissertations, 2 master’s theses) comprising 6331 clinical participants. Study quality was assessed using the QUADAS-2 tool. Results identified significant deficits in the Fluid Cognition Composite and the associated subtests (attention, working memory, processing speed, executive function) in clinical samples when compared to both the NIHTB-CB normative data and recruited comparison samples. Unexpectedly, there was some evidence that clinical participants scored higher on Crystallized Cognition subtests than the normative data but scored significantly lower than recruited controls. There was mixed evidence for performance differences on a Total Cognition Composite measure of cognitive function. There was some evidence of publication bias, and results were moderated by study quality and participant demographics. The implications of the findings for clinical research settings are discussed and suggested future directions are provided.

**Supplementary Information:**

The online version contains supplementary material available at 10.1007/s11065-025-09669-3.

Impairments in cognitive function are a prominent feature of a wide range of acute and chronic physical and mental health problems across the lifespan. Deficits in overall cognitive function, and more specifically in aspects of executive function and memory, are reflected in at least 40 published meta-analyses reporting on hundreds of studies that have included thousands of participants spanning early childhood through late adulthood (e.g., Abramovitch et al., 2019; Babikan & Asarnow, 2009; Campbell et al., 2007; Crivelli et al., 2022; Jim et al., 2012; Karsdorp et al., 2007; Moran, 2016; Naguib et al., 2009; Prussien et al., 2019; Robinson et al., 2010; Schillerstrom et al., 2005; Scott et al., 2015; Semkovska et al., 2019). Overall, findings from these reviews indicate there are significant deficits in broad and specific domains of cognitive function associated with medical and psychiatric conditions with effect sizes that range from small to large in magnitude. These meta-analyses have reported on studies using a wide range of different measures of cognitive function; however, there is no agreed upon or widely accepted battery of cognitive measures for use in clinical research. The large and growing research literature on cognitive impairment in patients with mental and physical health problems highlights the need for reliable and valid research tools for the careful and accurate measurement of cognitive function, given test results can have important implications for diagnosis, prognosis, and treatment.

The National Institutes of Health Toolbox–Cognition Battery (NIHTB-CB; Gershon et al., 2010; Weintraub et al., 2013a, 2013b) is a promising research tool that was developed as part of the NIH Blueprint for Neuroscience Research (https://neuroscienceblueprint.nih.gov/) to develop a brief and comprehensive test battery that could be used as a “common currency” to assess cognitive functioning across all stages of development, different study designs, and varying populations (Bauer & Zelazo, 2013; Gershon et al., 2020a, 2020b; Weintraub et al., 2013a, 2013b). The goal was to create a unifying research tool to measure outcomes in clinical trials and track cognitive status in large-scale longitudinal and epidemiological studies that would allow for direct comparisons of findings across diverse research initiatives, thereby maximizing the accumulation of knowledge within and across scientific disciplines (Bauer & Zelazo, 2014). A recent scoping review by Fox et al. (2022) documented its wide use in clinical research in over 200 published studies and conference abstracts in clinical populations across the lifespan.

To date, a quantitative review of the NIHTB-CB in clinical research has not been reported. The goal of the current review is to provide a meta-analysis of the utility of the NIHTB-CB in identifying expected cognitive deficits in individuals with medical and psychiatric conditions. We first summarize the development of the NIHTB-CB and review characteristics of this battery that are relevant to evaluating its use in clinical research including the coverage of cognitive domains, the quality and representativeness of the normative sample, types of scores that are generated, and its three versions. We then report the results of a meta-analysis examining its use in samples with diagnosed health problems and provide directions for future research with this instrument.

## Development of NIHTB-CB

### Coverage of Cognitive Domains

Priorities in the development of the NIHTB-CB were to create a set of cognitive tasks that were brief to administer, well-validated, and appropriate for individuals spanning a wide age range from 3 to 85 years old (Gershon et al., 2010). The specific domains of cognitive function included in the NIHTB-CB were identified through a survey of over 100 multidisciplinary research and clinical experts who ranked cognitive subdomains in order of their importance for neurodevelopment, health, and ease of measurement (Weintraub et al., 2013a, 2013b). Based on expert opinion and guided by a systematic literature review, the five cognitive subdomains included in the NIHTB-CB are (1) executive function and attention (Zelazo et al., 2013), (2) episodic memory (Bauer et al., 2013), (3) working memory (Tulsky et al., 2013), (4) processing speed (Carlozzi et al., 2013), and (5) language (Gershon et al., 2013). These five cognitive subdomains are represented in two broad cognition composites, Fluid and Crystallized Cognition, based on the two-component theory of intellectual development (Cattell, 1971; Horn, 1968). Lastly, a Total Cognition Composite score is generated.

#### Fluid Cognition Composite

Fluid cognitive abilities are the skills needed to quickly and efficiently process and encode new information and solve novel problems. These abilities are particularly important for learning and adapting to new environmental demands (Cattell, 1971). The NIHTB-CB generates a Fluid Cognition Composite (FCC) score that is derived from performance on five of the subtests. First, the Flanker Inhibitory Control and Attention (FICA) subtest is a measure of inhibitory control and attention in which the examinee is presented with a row of arrows and asked to select the way the middle arrow is pointing, which is either in the same direction as the flanking arrows (congruent) or in the opposite direction of the flanking arrows (incongruent). Second, the Dimensional Change Card Sort (DCCS) subtest is a measure of set-shifting and cognitive flexibility that requires the examinee to quickly select one of two options that matches either the color or shape of the presented stimulus. Third, the Pattern Comparison Processing Speed (PCPS) subtest is a measure of processing speed in which two stimuli are displayed and the examinee is asked to decide as quickly as possible if the pictures are identical. Fourth, the List Sorting Working Memory (LSWM) subtest is a measure of working memory in which the participant is presented with stimuli and asked to recall the items in order based on a specified rule. Fifth, the Picture Sequence Memory (PSM) subtest is a measure of episodic memory in which the examinee is presented with a series of pictures of various activities and asked to reproduce the sequences from memory.

Previous research has shown that skills that can be considered as part of fluid cognition are sensitive to neurological insult that can occur from health conditions. For example, attention, memory, and aspects of executive function are the most common domains impacted in patients with cancer (Ahles & Root, 2018; Pendergrass et al., 2018). A meta-analysis of neuropsychological outcomes in patients with breast cancer treated with chemotherapy, compared with healthy control participants, revealed the largest effect sizes in the domains of processing speed and language (Cohen’s *d* = − 0.39), executive function (Cohen’s *d* = − 0.34), and memory (Cohen’s *d* = − 0.30), but no differences compared with patients who were not treated with chemotherapy (Bernstein et al., 2017). In early stages of Alzheimer’s disease, fluid cognitive abilities decline more rapidly than crystallized abilities (McDonough et al., 2016). Research has also shown that individuals with a range of psychiatric disorders perform poorer on assessments of fluid cognition (Keyes et al., 2017). At the same time, deficits in fluid cognition have also been documented in individuals at risk for psychiatric problems. Specifically, research has shown that deficits in executive function confer risk for a range of psychiatric conditions (e.g., Catalan et al., 2021; McTeague et al., 2016, 2017).

#### Crystallized Cognition Composite

Crystallized cognition is broadly a measure of accumulated verbal knowledge and is more heavily influenced by one’s prior learning experiences and environment than fluid cognition (Cattell, 1971). These skills are particularly important for academic achievement and success in work. The NIHTB-CB generates a Crystallized Cognition Composite (CCC) score that is derived from performance on two subtests. Specifically, the Oral Reading Recognition (ORR) subtest is a measure of reading decoding in which the examinee is presented with a letter or word and asked to read it aloud. Second, the Picture Vocabulary (PV) subtest is a measure of receptive language in which the examinee is aurally presented with a word and asked to select one of four pictures that most closely represents the meaning of the word. Previous research has shown that crystallized abilities are less susceptible than fluid cognitive abilities to the effects of health conditions, including traumatic brain injury (Tulsky et al., 2017) and cardiovascular health (King et al., 2023), and that crystallized cognitive function remains relatively stable with age while fluid cognition declines with age (e.g., Heaton et al., 2014). Moreover, the *discrepancy* between fluid and crystallized cognitive function (i.e., lower fluid cognition as compared with crystallized cognition) is an early cognitive marker of dementia (e.g., Bajpai et al., 2022).

#### Cognitive Function Composite

The NIHTB-CB generates a Cognitive Function Composite (CFC) score that is a combination of fluid and crystallized cognitive abilities. During the development of the NIHTB-CB, most experts (57%) ranked a total cognitive score as valuable for large scale studies in which cognition is not the primary outcome of interest, but researchers are still interested in obtaining a broad measure of cognitive functioning.

### Normative Sample

To fully understand an examinee’s performance on a cognitive test, it is informative to have established normative data based on a representative sample of peers to provide a reference for the examinee’s expected performance. A large national normative study for the NIHTB-CB was conducted in 2011. As described in Beaumont et al. (2013), the normative sample included 4859 non-institutionalized community dwelling participants (3955 whose primary language was English; Carlozzi et al., 2015) ages 3–85 who were cognitively able to provide informed consent and assent from 10 US sites. The goal was to match the 2010 census data on race, ethnicity, and educational level. Quota sampling techniques were used to stratify the sample by age, sex, and language. It is noteworthy that the original normative sample was skewed toward younger age groups, with 100 to 150 participants representing each age for ages 3 to 8, over 200 participants for each age for ages 9 to 17, but only 100 to 300 participants representing each decade for ages 18 to 85 (Carlozzi et al., 2015).

### Score Types

The NIHTB-CB generates three types of standardized scores that are based on the normal curve where higher scores indicate better performance. Beaumont et al. (2013) details how the normative scores were computed.

#### Uncorrected Standard Scores

The Uncorrected Standard Score compares the examinee’s performance to the performance of all test-takers in the NIHTB-CB norming study regardless of age or other demographic characteristics. These scores provide a measure of the examinee’s overall abilities when compared to the general US population. The Uncorrected Standard Scores have a mean of 100 and a standard deviation of 15. These scores are primarily intended to monitor absolute change in cognitive abilities over time.

#### Age Corrected Standard Scores

The Age Corrected Standard Score (mean of 100, standard deviation of 15) compare the examinee’s performance to the average performance of test-takers in the normative sample at the same age as the examinee. NIH Toolbox normative scores are available for each year of age from 3 through 17, as well as for age brackets 18–29, 30–39, 40–49, 50–59, 60–69, 70–79, and 80–85. This was intended for targeted, accurate comparisons for any research study participant group with the US population.

#### Fully Corrected T Scores

Fully Corrected *T* Scores have a mean of 50 and a standard deviation of 10, and they were developed to account for key demographic variables that have been shown to impact test performance (Casaletto et al., 2015). These scores compare the performance of the examinee to those in the norming sample, while adjusting for age, sex, race/ethnicity (White/Asian, Black, Hispanic, multiracial), and educational attainment (education is often used as a proxy for socioeconomic status; parental education is used for examinees ages 3–17). A “fully corrected” score allows for comparison within a narrower grouping.

### Test Version

There have been three versions of the NIHTB-CB since its release. The original version (V1) was developed for administration on a web-based desktop computer and normative data were collected. In 2015, a second version (V2) was released after it was adapted to an iPad application for greater portability and ease of administration. Notably, there was not a re-norming of the measure when the transition was made from the desktop computer to the iPad version. In April 2023, a third version (V3) was released. Administration remains on an iPad and the seven subtests from V2 remain on V3, although novel subtests were also added, and new normative data were collected. Given that no published studies to date have used V3 and V2 will remain available for use until June 30, 2028, the focus of the meta-analysis will be on studies that used V1 and V2. We return to V3 in the “Discussion” section.

Despite the widespread and growing use of the NIHTB-CB in clinical research, concerns have been raised about the accuracy of V2 subtest scores for the FICA. These concerns are represented in a study that directly compared scores obtained on V1 with scores obtained on V2 (Brearly et al., 2019). Specifically, a sample of 49 combat-exposed post-deployment veterans without neurologic disorder, severe mental illness, current substance use disorder, or a history of moderate or severe traumatic brain injury completed the V1 and V2 cognitive battery on the same day in an experimental within-subjects crossover design. Significant performance differences were found between test versions on the FICA subtest such that participants performed better on V1 than V2. Scores were moderately correlated across tests, except for low correlations for the PCPS. Brearly et al. (2019) recommended caution when interpreting V2 scores of the NIHTB-CB, particularly for the FICA, and suggested the development of V2 norms from the iPad administration may be necessary to ensure valid interpretation of acquired data.

### Aims of the Current Study

The primary aim of the current meta-analysis was to quantitatively review and synthesize the available literature using the NIHTB-CB in clinical research from childhood through adulthood. The overarching goal was to review the degree to which the NIHTB-CB identifies performance differences between clinical samples and two comparison groups: (1) population normative data, and (2) recruited comparison samples. First, we hypothesized significant differences on the FCC for clinical samples when compared to normative data and recruited comparison samples, such that the clinical groups would obtain significantly lower scores. Analyses of the five fluid cognition subtests were exploratory to determine the extent to which deficits are detected in clinical samples on subdomains of fluid cognition. Second, we did not anticipate significant differences on the CCC or the two crystallized cognition subtests for clinical samples when compared to either normative data or recruited comparison samples. Third, given that the CFC is a composite of the FCC and CCC, analyses of the CFC between clinical groups and the normative data and recruited comparison samples were exploratory.

Based on previous research, we examined six possible moderators of effect sizes in clinical samples: (1) study quality, (2) NIHTB-CB test version, (3) participant age, (4) participant sex, (5) participant education, and (6) publication status. First, we examined the quality of the study using the QUADAS-2 tool (Whiting et al., 2011) as a potential moderator of the effects. Second, based on findings reported by Brearly et al. (2019), we hypothesized that the NIHTB-CB test version would moderate effects on the FICA, such that participants would perform significantly lower on V2 than V1. Analysis of test version as a moderator was exploratory for the remaining subtests and composites. Third, we examined participant age as a potential moderator. While the effects of health problems may be stronger with increasing age as a consequence of longer-term exposure to disease and treatments, alternatively, children and adolescents may experience more adverse effects to cognition because the developing brain may be more vulnerable to insult. Fourth, we examined sex effects, which have been noted on some subtests with females typically scoring higher than males, particularly in episodic memory (Casaletto et al., 2015; Heaton, Miller, et al., 2004). Fifth, education was examined as a potential moderator given it often correlates strongly with premorbid cognitive abilities (e.g., Lovden et al., 2020). And finally, publication bias may be reflected in larger effects in published as compared with unpublished studies.

## Method

The present meta-analysis was conducted in accordance with the Preferred Reporting Items for Systematic Reviews and Meta-Analyses (PRISMA) guidelines (Liberati et al., 2009; Moher et al., 2015). The current study was pre-registered through Open Science Framework (OSF) in June 2022. The study methods and hypotheses were included in the preregistration, which can be viewed at 10.17605/OSF.IO/UKW37. Samples that could not be classified as clinical groups (e.g., community samples, veterans, undergraduates) were initially included in the literature review as described in the pre-registration; however, it was ultimately determined that their inclusion was beyond the scope of this meta-analysis and were not included due to the extreme heterogeneity of these non-clinical samples.

### Literature Search

**Fig. 1:**
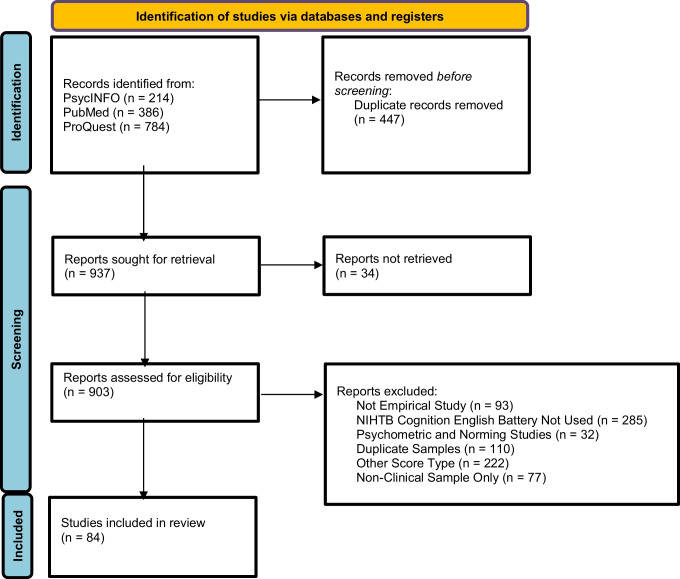
PRISMA flow diagram

A search for empirical reports published up until December 2021 was performed to identify peer-reviewed articles and unpublished dissertations and master’s theses that reported NIHTB-CB test scores. A systematic literature search was conducted using PsycINFO, PubMed, and ProQuest databases, with the specific search terms *National Institutes of Health Toolbox Cognition* OR *NIH Toolbox Cognition* across all fields (i.e., title, abstract, keywords). The initial search process yielded 1384 articles, doctoral dissertations, or master’s theses (see Fig. 1 for a PRISMA flow diagram). A review of the titles resulted in 937 unique records, 903 of which were accessible for review. Based on criteria outlined below, 84 studies were included in the quantitative meta-analysis. Book chapters, non-peer reviewed journal articles, and review articles were not included.

### Eligibility Criteria

The following criteria determined the selection of studies included in the meta-analysis: the NIHTB-CB was administered to participants ages 3 years and older using the English version, participants met criteria for a condition listed in the International Classification of Diseases 11th Revision (ICD-11; World Health Organization, 2022) or Diagnostic and Statistical Manual of Mental Disorders, Fifth Edition (DSM-5; American Psychiatric Association, 2013), the sample size was greater than two, the study was published in English, at least one subtest from the NIHTB-CB was administered, and the mean and standard deviation using the Fully Corrected *T* score or Age Corrected Standard score was reported. Articles were excluded if they only provided raw scores, computed scores, uncorrected scores, or did not specify the type of score used. In the case of duplicate samples, only one publication was included. In total, 110 studies were identified as being a duplicate sample, the vast majority (> 60%) of which were from large, national repositories of data such as the Adolescent Brain Cognitive Development study (National Institutes of Health & Centers, 2024) or the Human Connectome Project (HCP; Van Essen et al., 2012). For the remaining duplicate samples, peer-reviewed journal articles were prioritized. That is, for any thesis that was later published as a peer-reviewed journal article, data from the peer-reviewed journal article was used. If there were multiple peer-reviewed journal articles reporting findings from the same dataset, we then prioritized the article that provided the most relevant cognitive data. If multiple publications reported the same number of cognitive factors, we prioritized Fully Corrected *T* scores over Age Corrected scores. Lastly, if the relevant cognitive data were the same across publications, we chose the publication with the largest sample size.

The corresponding authors of peer-reviewed studies, dissertations, and master’s theses that were missing relevant statistics or study information (*n* = 116) were emailed in an effort to retrieve the relevant information for the analyses. We received 47 replies from corresponding authors (40.5%). Thirty-one authors provided at least some relevant data to be included, 16 authors responded but were unable to provide additional information, and 69 authors did not respond to the request for information. Characteristics of the included studies are presented in Table 1. An asterisk (*) indicates the corresponding author was emailed but did not respond to our request for information; however, there was still adequate information presented in the study to be included in the meta-analysis. A double asterisk (**) indicates that the corresponding author was emailed and responded to our request. A superscript ^1^ was placed next to information and data sent from the corresponding author to be included in the meta-analysis.

**Table 1:** Description of the included studies using the NIHTB-CB in clinical samples

Study	Clinical sample	Sub-Group	% F	*M* Age	*M* Edu	Control sample	Subtest	Composite	Version	**Score**	**Type**
Apple et al. (2017)**	Breast Cancer Survivors, *N* = 16	Cancer and Tumor	100	37.93	16.64	*N* = 18 100% F *M*_age_ = 27.17 *M*_edu_ = 16.22	FICA, DCCS, LSWM, PCPS, PSM, ORR, PV	FCC, CCC, CFC	V1^1^	FC^1^	PR
Avci et al. (2017)*	HIV, *N* = 52	HIV	9.62	55.86	13.63	*N* = 21 28.6% F *M*_age_ = 61.66 *M*_edu_ = 14.23	PSM		V1	FC	PR
Barton (2021)	Developmental Delays, *N* = 119	Neurodevelopment	21.5	9.24	NS		FICA, LSWM		V2	FC	T
Blank et al. (2020)**	Hearing Loss *N* = 39	Hearing Loss	49.0	6.64	NS	*N* = 41 41.0% F *M*_age_ = 5.67	FICA, DCCS^1^, PCPS^1^		V2	FC^1^	PR
Brewster et al. (2022)**	Insomnia, *N* = 28		82.1	65.14	15.14		FICA, DCCS, LSWM, PCPS, PSM, ORR, PV	FCC, CCC, CFC	V1	FC^1^	PR
Carlozzi et al. (2017)**	SCI, *n* = 158		22	47.47	14.03		FICA, DCCS, LSWM, PCPS, PSM, ORR, PV	FCC, CCC	V1	FC	PR
	TBI, *n* = 161	Acquired Brain Injury	36	39.38	14.08						
	Stroke, *n* = 175	Acquired Brain Injury	50	56.13	14.09						
Casaletto (2016)*	HIV and Substance Use Disorder, *N* = 90	HIV and Substance Use Disorder	11.1	49.50	13.17		FICA, DCCS, LSWM, PCPS, PSM		NS	FC	T
Cassetta et al. (2020)**	Treatment Resistant Psychosis, *N* = 38		21.1	35.55	12.00		FICA, DCCS, LSWM, PCPS, PSM, ORR, PV	FCC, CCC, CFC^1^	V2	FC^1^	PR
Chen et al. (2020)*	Breast Cancer Survivors, *N* = 19	Cancer and Tumor	100	66.60	NS	*N* = 14 100% F *M*_age_ = 68.10	FICA, DCCS, LSMW, PCPS, PSM, ORR, PV	FCC, CCC, CFC	NS	AC	PR
Clausen et al. (2021)**	TBI, *N* = 26	Acquired Brain Injury	15.4	43.30	NS	*N* = 33 24.2% F *M*_age_ = 47.00	FICA, DCCS, LSWM, PCPS, PSM^1^		V2	FC	PR
Cohen (2019)	Breast Cancer Survivors, *N* = 40	Cancer and Tumor	100	57.33	NS		FICA, DCCS, PSM		V2	FC	T
Davis et al. (2019)**	Parkinson’s Disease, *N* = 20	Neurodegenerative	45.0	63.40	NS	*N* = 19 63.2% F *M*_age_ = 62.68	FICA, DCCS, LSWM, PCPS, PSM, ORR, PV	FCC, CCC, CFC	V2^1^	FC^1^	PR
de Silva et al. (2021) **	Preterm Children *N* = 51		51.0	4.70	NS	*N* = 35 48.6% F *M*_age_ = 4.6	DCCS		V2^1^	FC	PR
Downes et al. (2022)**	Sickle Cell Silent Stroke, *n* = 10	Acquired Brain Injury	50.0	12.20	NS		FICA, DCCS, PCPS, PSM, PV		V2	AC	PR
	Sickle Cell No Stroke, *n* = 18		66.7	13.90	NS						
Dudley- Javoroski et al. (2022)*	SCI, *N* = 23		4.3	43.26	NS	*N* = 20 50.0% F *M*_age_ = 48.45	LSWM, PSM		V2	FC	PR
Dunbar et al. (2019)**	1 TBI, *n* = 41	Acquired Brain Injury	22.0	39.54	NS	*N* = 96 33.3% F *M*_age_ = 38.73		CFC	V2	FC	PR
	2 TBI, *n* = 47		26.2	41.21							
	3 TBI, *n* = 142		15.5	40.77							
Dutta (2020)**	Aphasia, *N* = 22	Acquired Brain Injury	36.0	63.68	16.63	*N* = 24 75.0% F *M*_age_ = 61.16 *M*_edu_ = 17.00	FICA, DCCS, LSWM		V2	FC^1^	T
Elias et al. (2020)*	Discharged from ICU, *N* = 30		38.0	70.64	NS		FICA, DCCS		V2	FC	PR
Eneva et al. (2017)*	BED, *n* = 23		100	23.34	15.39	*N* = 29 100% F *M*_age_ = 24.52 *M*_edu_ = 15.21	FICA, DCCS, LSWM, PCPS, PSM		V1	AC	PR
	OW, *n* = 48	Metabolic and Vascular	100	38.04	15.10						
	BED + OW *n* = 32	Metabolic and Vascular	100	36.34	13.84						
Engel (2018)**	Stroke and TBI, *N* = 10	Acquired Brain Injury	60.0	51.00	15.4		FICA, DCCS, LSWM, PCPS, PSM, ORR, PV	FCC, CCC, CFC	V2	AC	T
Frazer et al. (2018)	Cocaine Use Disorder, *N* = 20	Substance Use Disorder	30.0	46.25	12.8	*N* = 20 55.0% F *M*_age_ = 45.10 *M*_edu_ = 13.15	FICA, DCCS, LSWM, PCPS, PSM, ORR, PV	CFC	V1	FC	PR
French et al. (2021)**	Stroke *N* = 49	Acquired Brain Injury	46.9	64.90	NS		FICA^1^, DCCS^1^, LSWM^1^, PCPS^1^, PSM^1^	FCC	V2	FC^1^	PR
Gills et al. (2021)**	MCI, *N* = 11	Neurodegenerative	90.9	78.6	NS	*N* = 44 65.9% F *M*_age_ = 50.32	FICA, DCCS, PCPS, PSM		V2	FC^1^	PR
Gladstone et al. (2019)*	Mild TBI, *N* = 30	Acquired Brain Injury	56.7	15.36	NS			FCC, CCC	V1	AC	PR
Hackett et al. (2018)**	Alzheimer’s Disease, n = 14	Neurodegenerative Disease	38.0	78.4	14.7	*N* = 112 55.0% F *M*_age_ = 52.5 *M*_edu_ = 15.6	FICA, DCCS, PCPS, ORR, PV		V1^1^	AC^1^	PR
	Amnestic MCI *n* = 22	Neurodegenerative	48.0	74.3	15.4						
	Non-Amnestic MCI, *n* = 16	Neurodegenerative	32.0	71.8	15.4						
Hartman et al. (2018)**	Breast Cancer Survivors *N* = 87	Cancer and Tumor	100	57.2	NS		FICA, DCCS, LSWM, PCPS, PSM	FCC, CCC	NS	AC	PR
Hawkins et al. (2021a)*	OW/Obese *N* = 48	Metabolic and Vascular	85.4	43.58	NS		FICA, DCCS, LSWM, PCPS, PSM, ORR, PV		V2	FC	PR
Hawkins et al. (2021b)*	OW/Obese *N* = 103		73.0	45.37	NS		FICA, DCCS, LSWM, PCPS, PSM, ORR, PV		V2	FC	PR
Henry (2020)	Chronic Pain Taking Opioids *N* = 43	Pain Syndrome	58.1	59.70	NS		FICA, DCCS, LSWM, PCPS, PSM, ORR, PV	FCC, CCC, CFC	V2	FC	T
Hoffman et al. (2021)**	Cerebral Palsy *N* = 13	Neurodevelopment	NS	34.2	NS	*N* = 16 *M*_age_ = 34	FICA^1^, DCCS^1^, LSWM^1^, PCPS^1^,PSM^1^, ORR^1^, PV^1^	FCC, CCC^1^, CFC	V2^1^	FC^1^	PR
Hollister (2015)	Stutter, *N* = 46	Neurodevelopment	22.0	12.1	NS	*N* = 46 22.0% F *M*_age_ = 12.1	FICA, DCCS		V1	AC	T
Hoover et al. (2019)*	ASD, *N* = 23	Neurodevelopment	43.0	11.0	NS		PV		NS	AC	PR
Hwang et al. (2019)**	Temporal Lobe Epilepsy, *N* = 55	Epilepsy	54.5	40.1	14.2	*N* = 58 56.9% F *M*_age_ = 34.0 *M*_edu_ = 15.0	FICA, DCCS, LSWM, PCPS		V1	AC	PR
Kaat et al. (2022)**	Down Syndrome *n* = 54	Neurodevelopment	51.0	17.4	NS			FCC, CCC, CFC	V2	AC	PR
	Fragile X, *n* = 54	Neurodevelopment	28.0	18.5	NS						
	Intellectual Disability, *n* = 40	Neurodevelopment	35.0	17.5	NS						
Kalyani et al. (2019)*	Parkinson’s Disease, *N* = 33	Neurodegenerative	60.1	65.87	NS		FICA, DCCS, PCPS, PSM, PV		NS	FC	PR
Kamerer et al. (2019)**	Hearing Loss, *N* = 32	Hearing Loss	59.4	41.72^1^	NS		FICA, DCCS, LSWM, PCPS, PSM		V2	FC^1^	PR
Karawani et al. (2018)**	Hearing Loss, *N* = 32	Hearing Loss	59.4	74.56	NS		FICA, LSWM, PCPS		NS	AC	PR
Kim et al. (2021)**	Cirrhosis, *N* = 54	Metabolic and Vascular	38.9	61.00	NS		FICA, DCCS, LSWM, PCPS		V2	FC	PR
Koopowitz et al. (2021)**	MDD, *n* = 30		100	28.17	10.83	*N* = 86 100% F *M*_age_ = 27.56 *M*_edu_ = 11.65	FICA, DCCS, LSWM, PCPS		V1	AC^1^	PR
	PTSD, *n* = 35		100	28.86	10.78						
	PTSD + MDD *n* = 23		100	30.70	9.96						
Kratz et al. (2020)**	Fibromyalgia *N* = 50	Pain Syndrome	88.0	44.9	15.7	*N* = 50 88.0% F *M*_age_ = 45.2 *M*_edu_ = 15.8	FICA, DCCS, LSWM, PCPS		V2	FC	PR
Kringle (2019)**	Stroke, *N* = 24^1^	Acquired Brain Injury	61.9	70.81	NS		FICA^1^, DCCS^1^, LSWM^1^, PCPS^1^, ORR^1^, PV^1^	FCC^1^, CCC^1^, CFC	V2^1^	FC^1^	T
Kunker et al. (2020)*	TBI, *N* = 18	Acquired Brain Injury	77.8	29.11	NS	*N* = 14 64.3% F *M*_age_ = 23.79		FCC	V2	AC	PR
Ludyga et al. (2021)*	ASD, *N* = 174	Neurodevelopment	NS	10.5	NS	*N* = 202 *M*_age_ = 10.3	FICA, DCCS, LSWM, PCPS		NS	AC	PR
Lundine et al. (2018)**	TBI, *N* = 5	Acquired Brain Injury	60.0	16.02	NS	*N* = 50 52.0% F *M*_age_ = 15.49	FICA, DCCS, LSWM, PCPS, PSM	CFC	V1	AC^1^	PR
Maas et al. (2021)*	ICU, *N* = 42		38.1	65.38	NS		FICA, LSWM, PCPS		NS	FC	PR
MacIsaac (2018)	NSSI, Proximal, *N* = 39		94.9	20.92	NS	*N* = 194 82.5% F *M*_age_ = 21.61	FICA		V2	AC	T
	NSSI, Distal, *N* = 44		86.4	20.43							
Marinac et al. (2019)**	Breast Cancer Survivors, *N* = 30	Cancer and Tumor	100	62.20	NS		FICA, DCCS, LSWM, PCPS, PSM		NS	AC	PR
Mazzoli et al. (2021)**	Intellectual Disability, *N* = 21	Neurodevelopment	37.0	9.9	NS		FICA, LSWM		V2	AC^1^	PR
Meier et al. (2022)*	Stroke with Aphasia, *n* = 14	Acquired Brain Injury	42.9	62.29	14.21		FICA, DCCS, PCPS		V2	AC	PR
	Stroke, No Aphasia *n* = 15	Acquired Brain Injury	46.7	59.33	14.93						
Meredith et al. (2020)*	Alcohol Use Disorder, *N* = 77	Substance Use Disorder	27.3	44.10	13.90	*N* = 38 45.8% F *M*_age_ = 45.80 *M*_edu_ = 15.60	FICA, LSWM, PCPS, PSM, ORR		NS	FC	PR
Modi et al. (2018)**	Epilepsy, *N* = 18	Epilepsy	77.8	15.49	NS	*N* = 14^1^ 50.0% F *M*_age_ = 15.35^1^	FICA, DCCS, LSWM, PCPS^1^		V1	AC	PR
Molinaro et al. (2021)**	HIV, *N* = 208	HIV	45.0	11.6	NS	*N* = 208 52.0% F *M*_age_ = 12.0	FICA, DCCS, LSWM, PCPS		V2	AC^1^	PR
Moore et al. (2020)**	Prenatal Tobacco Exposure, *N* = 200		45.0	4.5	NS		FICA, DCCS, PV		V2	FC	PR
Moriarty (2019)*	Cardiovascular Disease, *N* = 20	Metabolic and Vascular	25.0	64.80	NS		FICA, DCCS, LSWM, PCPS, PSM		V2	FC	T
Morrow et al. (2020)**	TBI, *N* = 41	Acquired Brain Injury	53.6	37.10	14.90	*N* = 41 70.7% F *M*_age_ = 22.60 *M*_edu_ = 15.70	LSWM, PSM		V2	FC^1^	PR
Mulhauser et al. (2021)*	Obesity *N* = 117	Metabolic and Vascular	75.2	44.86	NS	*N* = 46 82.6% F *M*_age_ = 44.07	FICA, DCCS, LSWM, PCPS, PSM	FCC	V1	AC	PR
Norman et al. (2019)*	TBI, *n* = 20	Acquired Brain Injury	60.0	29.20	15.90		LSWM, PCPS, PV		NS	FC	PR
	Orthopedic Injury, *n* = 21		23.8	28.23	16.10						
Paolillio (2021)*	HIV and Heavy Alcohol Use, *N* = 23	HIV	4.0	56.90	14.00			FCC, CCC	V2	FC	T
Papalambros et al. (2019)*	MCI, *N* = 9	Neurodegenerative	55.6	72.00	16.00		FICA, DCCS, LSWM, PCPS, PSM, ORR, PV	FCC, CCC, CFC	NS	FC	PR
Pardej (2020)**	Neuro-fibromatosis, *N* = 20	Neurodevelopment	40.0	5.45	NS		FICA, DCCS, LSWM, PCPS^1^, PSM^1^, ORR^1^, PV^1^	CCC^1^	V2	AC	T
Pozar et al. (2020)*	MCI, *N* = 13	Neurodegenerative	85.0	73.62	14.23	*N* = 27 93.0% F *M*_age_ = 73.07 *M*_edu_ = 15.11	DCCS, LSWM, PCPS, PSM		V1	FC	PR
Prussien et al. (2021)**	Sickle Cell Anemia *N* = 60^1^		NS	17.20	NS		FICA^1^, DCCS^1^ LSWM^1^, PCPS^1^, PSM^1^	FCC	V2	AC	PR
Read et al. (2020)*	ADHD and Anxiety, *N* = 219	Neurodevelopment	23.0	10.54	NS		FICA, DCCS, LSWM, PCPS, PSM, ORR, PV	CFC	V2	AC	PR
Rebchuk et al. (2022)**	Stroke Survivors *N* = 53	Acquired Brain Injury	36.5	47.00	NS	*N* = 53 50.9% F *M*_age_ = 44.00	FICA^1^, DCCS^1^ LSWM^1^, PCPS^1^, PSM^1^ ORR^1^, PV^1^	FCC, CCC, CFC	V2	FC	PR
Richerson et al. (2021)*	End Stage Renal Disease, *N* = 23		34.8	66.30	NS		FICA, DCCS, LSWM, PCPS, PSM, ORR, PV	FCC, CCC, CFC	V2	AC	PR
Rockhold et al. (2021)**	Prenatal Alcohol Exposure, *N* = 49		53.1	11.67	NS	*N* = 46 47.8% F *M*_age_ = 12.02	FICA, DCCS, LSWM, PSM^1^		V2	FC	PR
Russell-Schulz et al. (2021)**	Mild TBI, *N* = 15	Acquired Brain Injury	46.7	37.20	15.50	*N* = 12 33.3% F *M*_age_ = 37.20 *M*_edu_ = 16.40		FCC, CCC, CFC^1^	V2	FC^1^	PR
Sanborn et al. (2022)*	Opioid Use Disorder, *N* = 177	Substance Use Disorder	48.0	42.19	NS		FICA, DCCS LSWM, PCPS PSM, ORR, PV	CFC	V2	FC	PR
Schmithorst et al. (2022)*	Congenital Heart Disease, *N* = 27		29.6	14.5	NS	*N* = 53 50.9% F *M*_age_ = 14.4	FICA, DCCS, LSWM, PCPS, PSM, ORR, PV	FCC, CCC, CFC	V1	AC	PR
Scholl et al. (2021)**	Parkinson’s Disease, *N* = 81	Neurodegenerative	30.0	68.7	15.80	*N* = 41 43.9% F *M*_age_ = 71.3 *M*_edu_ = 16.60	FICA, DCCS, PCPS, PSM, PV		V2^1^	FC	PR
Shapiro et al. (2021)**	Type I Diabetes *n* = 1095	Metabolic and Vascular	53.2	21.00	NS		FICA, DCCS, LSWM, PCPS, PSM	FCC	NS	AC	PR
	Type II Diabetes *n* = 285		70.5	24.60	NS						
Shiau et al. (2021)*	HIV, *N* = 69	HIV	49.3	64.60	NS	*N* = 38 42.1% F *M*_age_ = 66.00	FICA, DCCS, LSWM, PCPS, ORR		V2	FC	PR
Siciliano et al. (2021)**	Hypoplastic Left Heart Syndrome *N* = 20		20.0	11.20	NS		FICA^1^, DCCS^1^, LSWM^1^, PCPS^1^, PSM^1^	FCC	V2	AC	PR
Siciliano et al. (2022)**	Brain Tumor *N* = 10	Cancer and Tumor	39.0	11.98	NS		FICA^1^, DCCS^1^, LSWM^1^, PCPS^1^, PSM^1^	FCC	V1	FC^1^	PR
Sinha et al. (2018)*	Oropharyngeal Cancer, *N* = 56	Cancer and Tumor	7.1		NS		FICA, DCCS, LSWM, PCPS, PSM, ORR, PV	FCC, CCC, CFC	NS	FC	PR
Slack (2018)	COPD, *N* = 19		63.2	68.15	NS		FICA, DCCS, LSWM, PCPS, PSM, ORR, PV	FCC, CCC, CFC	NS	FC	T
Solomon et al. (2021)*	ASD, *N* = 66	Neurodevelopment	16.7	17.5	NS	*N* = 66 22.7% F *M*_age_ = 17.5	FICA, DCCS, LSWM, PCPS, PSM, ORR, PV	FCC, CCC	V2	AC	PR
Terry et al. (2019)*	TBI, *n* = 49	Acquired Brain Injury	50.0	41.0	NS		FICA, DCCS, LSWM, PCPS, PSM	FCC	V1	FC	PR
	TBI + Depression *n* = 27	Acquired Brain Injury	50.0	41.0	NS						
Thomas et al. (2021)	TBI *N* = 16	Acquired Brain Injury	53.3	47.7	NS		FICA, DCCS, LSWM, PCPS, PSM, ORR, PV	FCC, CCC, CFC	V2	FC	PR
Thompson et al. (2020)	Epilepsy, *N* = 38	Epilepsy	58.0	10.6	NS		FICA, DCCS, LSWM, PCPS, PSM, ORR, PV	FCC, CCC, CFC	V1	AC	PR
Wakaizumi et al. (2021)*	Chronic Back Pain: Opioid Therapy, *n* = 29	Pain Syndromes	55.2	58.60	NS		FICA, DCCS, LSWM, PCPS, PSM, PV		NS	FC	PR
	Chronic Back Pain: No Therapy, *n* = 29		55.2	57.70	NS						
Watson et al. (2020)*	TBI, *n* = 13	Acquired Brain Injury	36.0	11.40	6.00		FICA, DCCS, LSWM, PCPS, PSM, ORR, PV	FCC, CCC, CFC	NS	AC	PR
	Vascular, *n* = 10		50.0	11.40							
	Encephalitis, *n* = 7		43.3	11.40							
	Brain Tumor, *n* = 4	Cancer and Tumor	50.0	11.40							
	Other Brain Injury, *n* = 5	Acquired Brain Injury	60.0	11.40							
Zimmerman et al. (2019)*	Chronic Insomnia, *N* = 14			46.50	15.86		FICA, DCCS, LSWM, PCPS, PV		V1	AC	PR
Zuniga and Moran (2018)*	Breast Cancer Survivors, *N* = 29	Cancer and Tumor	100	50.10	NS	*N* = 38 100% F *M*_age_ = 50.80	LSWM, PSM, ORR, PV		V1	AC	PR

*TBI* traumatic brain injury, *SCI* spinal cord injury, *ICU* intensive care unit, *BED* binge eating disorder, *OW* overweight, *MCI* mild cognitive impairment, *ADHD* attention-deficit/hyperactivity disorder, *COPD* chronic obstructive pulmonary disease, *ASD* autism spectrum disorder, *NSSI* non-suicidal self injury, *MDD* major depressive disorder, *PTSD* posttraumatic stress disorder, *M* mean, *F* female, *Edu* education, *FICA* flanker inhibitory control attention, *DCCS* dimensional change card sort, *LSWM* list sort working memory, *PCPS* pattern comparison processing speed, *PSM* picture sequence memory, *ORR* oral reading recognition, *PV* picture vocabulary, *FCC* fluid cognition composite, *CCC* crystallized cognition composite, *CFC* cognitive function composite, *V1* version 1 (Desktop), *V2* version 2 (iPad), *NS* not specified, *AC* age corrected, *FC* fully corrected, *PR* peer-reviewed article, *T* thesis

* = corresponding author was emailed and did not respond to our request

** = corresponding author responded to request, ^1^ = data provided by author

### Data Extraction and Coding

A spreadsheet was developed and used to extract the relevant data from selected articles. The spreadsheet included (1) study information (name of the first author, year of publication, country of origin, and peer-reviewed article/thesis); (2) description of the sample(s) and size; (3) demographic information of the sample(s), including mean age, mean education, and percent of the sample who identified as female; (4) test version (i.e., V1, V2, unspecified); (5) the types of standardized scores reported (i.e., fully corrected, age corrected); and (6) the means and standard deviations for the subtest and composite scores. The included articles and theses were divided among the authors who conducted the extraction independently. The reliability of data entry was checked on 20 randomly selected articles (24%) and there were three errors identified out of the 292 pieces of data entered (1%).

The study was included if the study participants met criteria for a condition listed in the ICD-11 or DSM-5. Studies that included comorbid medical and/or psychiatric conditions were included given comorbid health conditions are common across the lifespan (e.g., Jones et al., 2004; Sharma et al., 2019). There was significant heterogeneity and minimal diagnostic overlap in the clinical samples of the included studies (see Table S1 for a list of conditions). To facilitate interpretation of the broad clinical category, subgroup analyses were also conducted for specific types of diseases and disorders when at least three studies reported findings for the NIHTB-CB subtest or composite. Ten disease specific subgroups were identified and coded by the multidisciplinary team of authors, including a physician, two clinical psychologists, and two clinical psychology doctoral students (see Table S1). This collaborative approach ensured a comprehensive and accurate classification of subgroups.

### Risk of Bias Assessment

The methodological quality and potential bias of each study was assessed by the co-authors using the Quality Assessment of Diagnostic Accuracy Studies–2 instrument (QUADAS-2; Whiting et al., 2011). The QUADAS-2 is a structured tool developed to assess the potential bias of included studies in meta-analyses with the signaling questions within each domain modified for relevance to the present meta-analysis (see Table S2). “Risk of bias” was assessed in four domains: patient selection, index test (i.e., NIHTB-CB), reference standard (i.e., clinical diagnosis), and flowing and timing. Patient selection and reference standard domains were further assessed regarding their applicability to the review question. The reviewers extracted data from all included studies using a predetermined form and rated each domain as “low risk of bias,” “high risk of bias,” or “unclear risk of bias” based on the established criteria. Thirty-four publications (41%) were assessed by two reviewers and disagreements were resolved through consensus. During consensus meetings, the two reviewers each explained their reasoning for any domain where they disagreed on the rating using relevant information and data they extracted from the study (e.g., description of how participants were recruited). Based on the discussion, the reviewers agreed on a consensus rating.

### Calculation of Effect Sizes

**Fig. 2:**
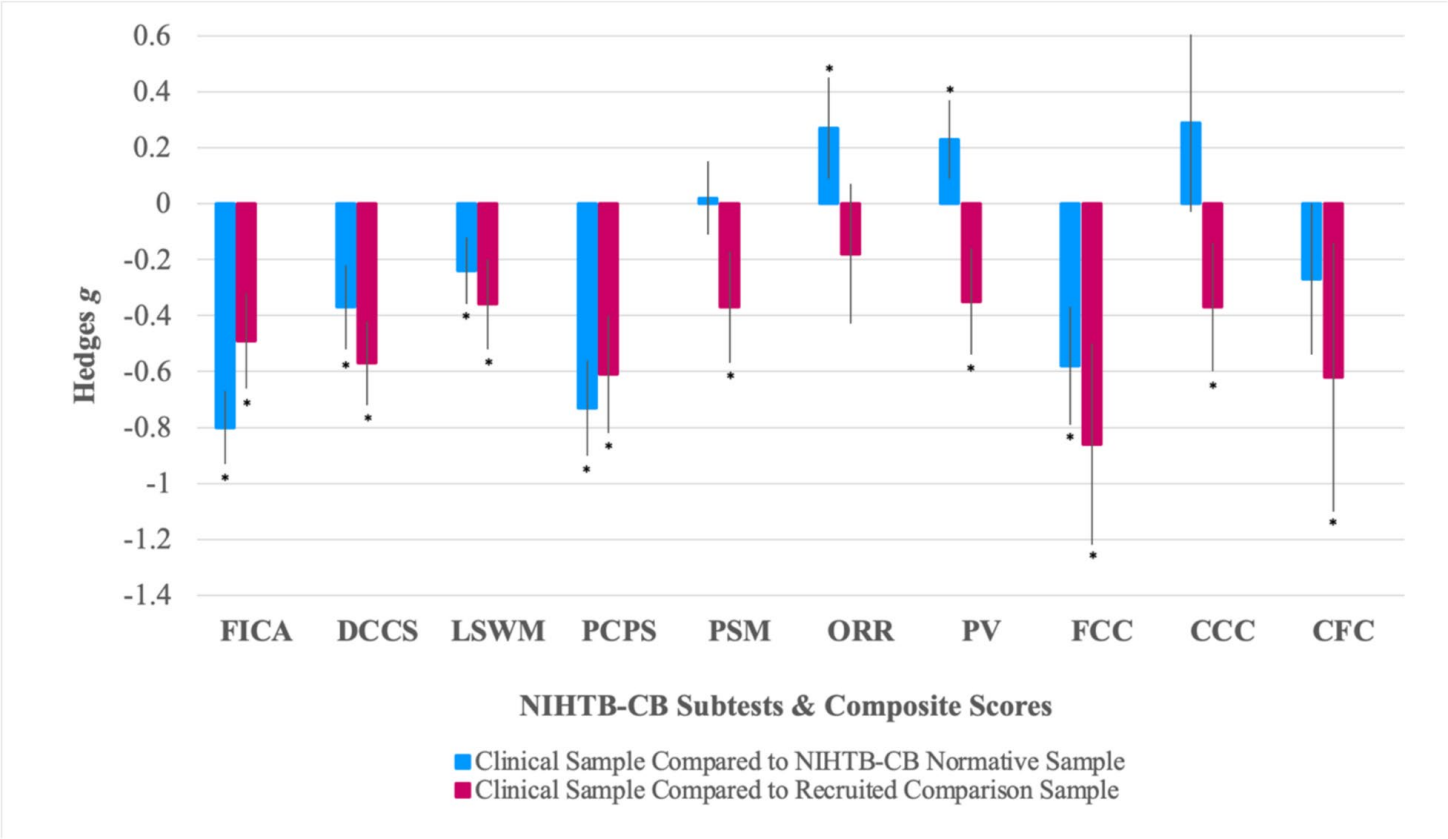
Effect sizes comparing clinical samples to normative data and recruited comparison samples. *FCC* fluid cognition composite, *CCC* crystallized cognition composite, *CFC* cognitive function composite, *FICA* flanker inhibitory control attention test, *DCCS* dimensional change card sort test, *LSWM* list sort working memory test, *PCPS* pattern comparison processing speed test, *PSM* picture sequence memory test, *ORR* oral reading recognition test, *PV* picture vocabulary test, *p < .05

Analyses were conducted with the Comprehensive Meta-Analysis (CMA) Software Version 4 (Borenstein, Hedges, Higgins, & Rothstein, 2005). Hedge’s *g* was calculated using a random effects model to determine the number of standard deviations by which the performance of the clinical study samples differed from the NIHTB-CB normative sample performance (Table 2) or from a recruited comparison sample (Table 3). A positive Hedge’s *g* indicates that the clinical study sample performed better than the normative or recruited comparison sample, while a negative Hedge’s *g* indicates the performance of the clinical study sample was below the performance of the normative or recruited comparison sample (see Fig. 2). Tables 2 and 3 also include the standard error, variance, and 95% confidence interval for each effect. Significant effects are reflected in 95% confidence intervals that do not cross zero. For significant effects, the magnitude is interpreted based on Cohen’s *d* criteria where 0.20 is a small effect, 0.50 is a medium effect, and 0.80 is a large effect (Cohen, 1988). For each effect, we also report the (1) *Z* value, a test of the null effect where a larger value indicates a higher probability that the groups are significantly different from each other; (2) Tau, an estimate of the standard deviation of the effects across studies; (3) Q statistic and corresponding degrees of freedom, an estimate of total within group heterogeneity; and (4) *I*^2^, which represents the proportion of variance in the observed effect that is due to heterogeneity rather than sampling error (Higgins et al., 2003). No steps were required to prepare the data for presentation or synthesis (e.g., missing summary statistics, data conversions).

**Table 2:** Clinical sample compared to NIHTB-CB normative sample

Subtest/composite	*k*	Point estimate	SE	Variance	95% CI	Z value	Tau	*Q* (df)	I^2^
**Clinical**									
CFC	25	− .27	.14	.02	[− .54, .00]	− 1.93	.65	**337.00 (24)**	92.88
FCC	33	− **.58**	.10	.01	**[**− **.78,** − **.37]**	− 5.54	.55	**329.22 (32)**	90.28
CCC	26	.29	.17	.03	[− .04, .61]	1.73	.81	**479.36 (25)**	94.79
FICA	70	− **.80**	.07	.01	**[**− **.93,** − **.67]**	− 11.83	.53	**795.04 (69)**	91.32
LSWM	63	− **.24**	.06	.00	**[**− **.36,** − **.12]**	− 3.91	.44	**555.94 (62)**	88.85
DCCS	66	− **.37**	.08	.01	**[**− **.52,** − **.22]**	− 4.78	.59	**908.33 (65)**	92.84
PCPS	63	− **.73**	.09	.01	**[**− **.91,** − **.56]**	− 8.82	.68	**1140.49 (62)**	94.56
PSM *Adjusted value*	54	− **.13** .02	.06	.00	**[**− **.25,** − **.02]** [− .10, .15]	− 2.23	.39	**349.42 (53)** **553.65**	84.83
ORR *Adjusted value*	31	**.45** **.27**	.09	.01	**[.27, .63]** **[.09, .45]**	4.90	.47	**271.96 (30)** **399.29**	88.97
PV	38	**.23**	.07	.01	**[.09, .37]**	3.20	.39	**237.01 (37)**	84.39
**Acquired brain injury**									
CFC	8	− .25	.22	.05	[− .68, .18]	− 1.15	.56	**49.86 (7)**	85.96
FCC	11	− **.58**	.14	.02	**[**− **.85,** − **.31]**	− 4.23	.39	**53.11 (10)**	81.17
CCC	8	**.30**	.15	.02	**[.01, .60]**	2.01	.35	**29.59 (7)**	76.34
FICA *Adjusted Value*	13	− **.89** − **.75**	.10	.01	**[**− **1.09,** − **.70]** **[**− **.97,** − **.54]**	− 9.11	.27	**34.36 (12)** **58.67**	65.07
LSWM *Adjusted value*	13	− **.33** − .14	.10	.01	**[**− **.53,** − **.13]** [− .36, .08]	− 3.18	.30	**43.86 (12)** **106.86**	72.64
DCCS	13	− **.65**	.26	.07	**[**− **1.15,** − **.14]**	− 2.52	.89	**257.35 (12)**	95.34
PCPS	13	− **.92**	.15	.02	**[**− **1.21,** − **.63]**	− 6.23	.47	**81.44 (12)**	85.26
PSM *Adjusted value*	12	− **.33** − .26	.13	.02	**[**− **.58,** − **.08]** [− .53, .01]	− 2.59	.38	**56.94 (11)** **69.73**	80.68
ORR	6	.30	.16	.03	[− .02, .62]	1.86	.33	**20.58 (5)**	75.70
PV	8	.25	.13	.02	[.00, .49]	1.99	.27	**20.18 (7)**	65.31
**Cancer and tumor**									
CFC	4	.00	.48	.23	[− .94, .94]	− .01	.90	**43.53 (3)**	93.11
FCC	6	− .43	.27	.07	[− .95, .10]	− 1.60	.59	**39.69 (5)**	87.40
CCC	5	**.68**	.31	.10	**[.08, 1.29]**	2.21	.63	**37.94 (4)**	89.46
FICA	8	− **.43**	.13	.02	**[− .67, − .18]**	− 3.42	.26	**16.60 (7)**	57.82
LSWM	8	**.24**	.11	.01	[**.03**, **.45**]	2.26	.20	13.30 (7)	47.36
DCCS	8	− .04	.16	.02	[− .35, .26]	− .28	.36	**25.85 (7)**	72.92
PCPS	7	− **.58**	.26	.07	**[**− **1.08,** − **.07]**	− 2.23	.63	**57.64 (6)**	89.59
PSM	9	.26	.17	.03	[− .08, .59]	1.50	.45	**42.14 (8)**	81.02
ORR	5	**.89**	.15	.02	**[.60, 1.17]**	6.10	.22	7.69 (4)	47.98
PV	5	.56	.42	.18	[− .26, 1.39]	1.34	.90	**65.67 (4)**	93.91
**Epilepsy**									
FICA	3	− .73	.68	.46	[− 2.06, .60]	− 1.07	1.16	**73.67 (2)**	97.29
LSWM	3	− **.66**	.33	.11	**[**− **1.30,** − **.01]**	− 2.00	.53	**15.39 (2)**	87.00
DCCS	3	− **.86**	.39	.15	**[**− **1.61,** − **.10]**	− 2.23	.64	**22.79 (2)**	91.23
PCPS	3	− 1.16	.64	.42	[− 2.42, .10]	− 1.80	1.10	**65.83 (2)**	96.96
**Hearing Loss**									
FICA	3	− .40	.30	.09	[− .98, .18]	− 1.34	.47	**13.22 (2)**	84.87
PCPS	3	− .38	.43	.19	[− 1.23, .47]	− .87	.71	**22.07 (2)**	90.94
**Human Immunodeficiency Virus**									
FICA	3	− **.94**	.45	.20	**[**− **1.81,** − **.06]**	− 2.09	.76	**61.76 (2)**	96.76
LSWM	3	− **.64**	.18	.03	**[**− **.99,** − **.29]**	− 3.54	.28	**10.53 (2)**	81.00
DCCS	3	− .68	.50	.25	[− 1.67, .30]	− 1.37	.86	**80.93 (2)**	97.53
PCPS	3	− 1.03	.69	.47	[− 2.38, .31]	− 1.50	1.18	**147.64 (2)**	98.65
**Metabolic and vascular**									
FCC	3	− .25	.31	.10	[− .86, .36]	− .82	.52	**37.33 (2)**	94.64
FICA	7	− **.95**	.17	.03	**[**− **1.28,** − **.62]**	− 5.66	.42	**60.21 (6)**	90.04
LSWM	7	− .06	.13	.02	[− .32, .20]	− .43	.32	**40.26 (6)**	85.10
DCCS	7	− .15	.18	.03	[− .50, .20]	− .82	.45	**73.76 (6)**	91.87
PCPS	7	− .16	.19	.03	[− .52, .21]	− .84	.47	**78.29 (6)**	92.34
PSM	6	.10	.16	.03	[− .22, .42]	.61	.37	**45.05 (5)**	88.90
PV	3	.24	.24	.06	[− .24, .72]	− 5.70	.40	**23.14 (2)**	91.36
**Neurodegenerative disorder**									
FICA	6	− **.94**	.09	.01	**[**− **1.11,** − **.76]**	− 10.35	.00	.37 (5)	.00
LSWM	3	− .04	.39	.15	[− .80, .72]	− .10	.60	**10.94 (2)**	81.72
DCCS	7	− .11	.08	.01	[− .28, .05]	− 1.36	.00	4.91 (6)	.00
PCPS	7	− **1.01**	.16	.03	**[**− **1.33,** − **.70]**	− 6.22	.34	**18.09 (6)**	66.82
PSM	6	− **.45**	.19	.04	**[**− **.83,** − **.07]**	− 2.32	.40	**18.84** (**5**)	73.47
ORR	4	**.60**	.21	.05	**[.18, 1.01]**	2.79	.33	7.22 (3)	58.46
PV	6	**.38**	.09	.01	**[.20, .55]**	4.27	.00	2.74 (5)	.00
**Neurodevelopmental disorder**									
CFC	3	− 1.07	.72	.52	[− 2.49, .35]	− 1.48	1.23	**63.07 (2)**	96.83
FCC	3	− **1.52**	.68	.47	**[**− **2.86,** − **.18]**	− 2.22	1.16	**50.87 (2)**	96.07
CCC	4	− .45	.83	.70	[− 2.09, 1.18]	− .54	1.65	**237.72 (2)**	98.74
FICA	8	− **.70**	.21	.04	**[**− **1.10,** − **.29]**	− 3.40	.55	**145.94 (7)**	95.20
LSWM	7	− **.56**	.18	.03	**[**− **.92,** − **.20]**	− 3.06	.45	**87.04 (6)**	93.11
DCCS	6	− .32	.21	.04	[− .72, .08]	− 1.56	.22	**77.22 (5)**	93.53
PCPS	5	− **.82**	.24	.06	**[**− **1.30,** − **.35]**	− 3.38	.51	**76.10 (4)**	94.74
PSM	4	.02	.10	.01	[− .18, .22]	.22	.13	5.26 (3)	42.91
ORR	4	.23	.20	.04	[− .16, .61]	1.17	.34	**18.89 (3)**	84.12
PV	5	.08	.17	.03	[− .24, .41]	.49	.32	**20.58 (4)**	80.57
**Pain syndrome**									
FICA	3	− **1.10**	.12	.02	**[**− **1.35,** − **.86]**	− 8.88	.13	3.20 (2)	37.53
LSWM	3	− .14	.10	.01	[− .33, .04]	− 1.52	.00	.51 (2)	.00
DCCS	3	− **.22**	.10	.01	**[**− **.31,** − **.03]**	− 2.32	.00	1.81 (2)	.00
PCPS	3	− .43	.22	.05	[− .86, .01]	− 1.93	.34	**10.43 (2)**	80.83
**Substance use disorder**									
FICA	4	− **.66**	.29	.09	**[**− **1.23,** − **.09]**	− 3.37	.56	**47.65 (3)**	93.70
LSWM	4	− .37	.22	.05	[− .79, .06]	− 1.67	.41	**27.81 (3)**	89.21
DCCS	3	− **.49**	.25	.06	**[**− **.96,** − **.01]**	− 1.98	.39	**13.06 (2)**	84.69
PCPS	4	− **.45**	.09	.01	**[**− **.62,** − **.28]**	− 5.04	.11	4.75 (3)	36.80
PSM	4	− .22	.13	.02	[− .48, .04]	− 1.68	.21	**9.95 (3)**	69.83
ORR	3	**.76**	.16	.02	**[.46, 1.06]**	4.92	.23	**7.69 (2)**	73.98

*FCC* fluid cognition composite, *CCC* crystallized cognition composite, *CFC* cognitive function composite, *FICA* flanker inhibitory control attention test, *DCCS* dimensional change card sort test, *LSWM* list sort working memory test, *PCPS* pattern comparison processing speed test, *PSM* picture sequence memory test, *ORR* oral reading recognition test, *PV* picture vocabulary test. *k* number of studies, *SE* standard error, *CI* confidence interval, *df* degrees of freedom. Bolded text indicates that the effect size was significant at the *p* < .05 level and the 95% CI does not contain zero. Adjusted values were calculated for any effect size in which the trim and fill analyses indicated significant publication bias. Adjusted values indicate the effect size when the trim and fill procedures are applied

**Table 3:** Clinical sample compared to recruited comparison samples

Subtest/composite	*k*	Point estimate	SE	Variance	95% CI	Z value	Tau	*Q* (df)	I^2^
**Clinical**									
CFC	8	− **.62**	.24	.06	**[**− **1.10,** − **.14]**	− 2.20	.60	**31.54 (7)**	77.80
FCC	10	− **.86**	.19	.03	**[**− **1.22,** − **.50]**	− 4.62	.49	**36.96 (9)**	75.65
CCC	8	− **.37**	.12	.01	**[**− **.59,** − **.14]**	− 3.13	.16	9.42 (7)	25.65
FICA	27	− **.49**	.09	.01	**[**− **.66,** − **.32]**	− 5.64	.37	**98.60 (26)**	73.63
LSWM	25	− **.36**	.08	.01	**[**− **.52,** − **.20]**	− 4.47	.31	**68.41 (24)**	64.92
DCCS	27	− **.57**	.08	.01	**[**− **.73,** − **.42]**	− 7.28	.31	**71.48 (26)**	63.63
PCPS	24	− **.61**	.11	.01	**[**− **.82,** − **.40]**	− 5.70	.45	**107.26 (23)**	78.56
PSM *Adjusted value*	21	− **.46** − **.37**	.10	.01	**[**− **.64,** − **.28]** **[**− **.56,** − **.17]**	− 4.84	.34	**55.98 (20)** **77.85**	64.27
ORR	13	− .18	.13	.02	[− .43, .07]	− 1.45	.36	**36.30 (12)**	66.95
PV	12	− **.35**	.10	.01	**[**− **.54,** − **.16]**	− 3.63	.20	**17.61 (11)**	37.52

*FCC* fluid cognition composite, *CCC* crystallized cognition composite, *CFC* cognitive function composite, *FICA* flanker inhibitory control attention test, *DCCS* dimensional change card sort test, *LSWM* list sort working memory test, *PCPS* pattern comparison processing speed test, *PSM* picture sequence memory test, *ORR* oral reading recognition test, *PV* picture vocabulary test. *k* number of studies, *SE* standard error, *CI* confidence interval, *df* degrees of freedom. Bolded text indicates that the effect size was significant at the *p* < .05 level and the 95% CI does not contain zero. Adjusted values were calculated for any effect size in which the trim and fill analyses indicated significant publication bias. Adjusted values indicate the effect size when the trim and fill procedures are applied

Effect sizes in meta-analyses based on small number of studies are subject to problems in data synthesis (Davey, Turner, Clarke, & Higgins, 2011); therefore, we set a threshold of a minimum of 5 studies to estimate effect sizes for the NIHTB-CB subtests and composite scores when compared to the NIHTB-CB normative sample. Given the minimal overlap in disease-specific subgroups across the studies (see Table S1), we set the threshold of a minimum of 3 studies to compare clinical subgroups (e.g., cancer and tumor) to the normative sample. Similarly, because relatively few studies recruited a comparison sample (*k* = 37), we set the threshold at a minimum of 3 studies to estimate effects for clinical samples compared to a recruited comparison sample.

### Moderation

Six variables were considered as potential moderators of effects of cognitive functioning in clinical samples in the CMA Program. Moderators included risk of bias, mean age, version of the NIHTB-CB (i.e., V1, V2), sex (i.e., percent female), mean education, and publication status (i.e., peer-reviewed journal article, thesis). Moderators were investigated for each of the NIHTB-CB subtests and composites. Continuous variables (i.e., mean age, percent female, mean level of education) were examined using meta-regression (see Table S3). Categorical variables (i.e., test version, publication type, risk of bias) were calculated through comparative subgroup analysis (see Table S4).

### Publication Bias

Several metrics were employed to test for potential publication bias which can occur when studies with small or non-significant effects are less likely to be published than results that are statistically significant. Publication bias was assessed for effects that aggregated results from at least 11 studies to ensure adequate power (Dalton et al., 2016). First, funnel plots for each analysis were visually inspected for evidence of biased reporting. Studies with larger sample sizes appear at the upper portion of the funnel plot while studies with smaller sample sizes appear at the lower portion. While it is expected that the studies will be distributed symmetrically around the combined effect size, small study publication bias is demonstrated by an asymmetric distribution at the lower portion of the funnel plot, indicating that smaller sized studies with larger than average effects were more likely to be published (Sedwick & Marston, 2015). Then, two additional statistics were computed to aid in quantification and interpretation of bias: Egger’s linear regression test (Egger et al., 1997) as well as Duval and Tweedie’s trim and fill analysis that corrects for publication bias (Duval & Tweedie, 2000). The Egger’s test is a statistical approach to assessing funnel plot asymmetry and the impact of study size by regressing the standardized effect on the inverse of the standard error. Trim and fill analysis evaluates plot asymmetry and estimates the number of studies that would need to be added to correct the skew in the distribution. The trim and fill technique involves two steps: (1) “trimming” the studies thought to contribute to bias on one side of the funnel plot, and (2) “filling in” these missing studies on other side of the funnel plot to create symmetry. The analysis then calculates a bias-adjusted point estimate and confidence interval to correct for the publication bias.

## Results

A total of 84 studies were included in the meta-analysis (71 peer-reviewed articles, 11 dissertations, 2 master’s thesis) with 6331 clinical participants. A summary of the characteristics of the included studies are reported in Table 1, including the sample size, mean age, percentage female, and mean education of the enrolled participants, when provided. There were 52 distinct clinical conditions that reported cognitive performance using the NIHTB-CB, such as cancer, stroke, traumatic brain injury, epilepsy, depression, posttraumatic stress disorder, and substance use disorder. Although the clinical conditions were heterogenous, there were a sufficient number of studies to examine cognitive functioning in ten narrow clinical subgroups (see Table S1): (1) acquired brain injury (ABI), (2) cancer and tumor, (3) epilepsy, (4) hearing loss, (5) human immunodeficiency virus (HIV), (6) metabolic and vascular diseases, (7) neurodegenerative disorders, (8) neurodevelopmental disorders, (9) pain syndromes, and (10) substance use disorders. Results are presented aggregated across all studies as a Hedge’s *g* in Tables 2 and 3. Summary statistics for each individual study are available from the authors upon request.

### Risk of Bias of Included Studies

Results of the QUADAS-2 for the included studies are summarized in Table 4 and displayed graphically in Figs. S1 and S2. Studies were not excluded based on their risk of bias or applicability concerns. Inter-rater agreement was acceptable (Cohen’s kappa = 0.77; McHugh, 2012). Only three studies were rated as having low risk of bias in all assessed categories; the remaining 81 studies had concerns in at least one of the domains (patient selection, index test, reference standard, flow and timing). Regarding patient selection risk of bias, 50.0% of studies (*k* = 42) were judged as being high risk, which was mostly due to recruitment methods that used a design other than a random or consecutive sampling procedure. Random or consecutive sampling reduces the risk of bias because all patients with a particular clinical diagnosis have an equal chance to be enrolled in the study and is therefore more representative of the target condition. Additionally, 38.1% of studies (*k* = 32) were judged as unclear risk of bias in patient selection. Regarding applicability concerns for patient selection, 15.5% (*k* = 13) were rated as high concern for reasons such as requiring cognitive assessment prior to participant inclusion, potentially introducing bias to their subsequent performance on the NIHTB-CB. Regarding the index test of the NIHTB-CB, only two studies (2.3%) were rated as high risk because the study explicitly stated the administration was not standardized. A remaining three studies (3.6%) were rated as unclear due to insufficient details around testing procedures. Regarding the reference standard risk of bias, one study was rated as high risk, whereas 31 studies (36.9%) were rated as unclear risk for lack of details on who administered the reference standard and how it was interpreted. There were applicability concerns for the reference standard for four studies largely due to lack of clarity on confirmation of medical or psychiatric diagnoses upon enrollment. Regarding flow and timing, 25.0% had high risk of bias and 34.5% had unclear risk of bias, which was primarily due to > 10% of enrolled participants being excluded from primary analyses (or lack of clarity about the proportion excluded), and lack of clarity regarding whether all patients received the same reference standard. Overall, these QUADAS-2 ratings highlight the variability in the quality of the included studies in the following domains: (1) patient selection risk of bias, (2) patient selection applicability, (3) reference standard risk of bias, and (4) flow and timing risk of bias. Given there was minimal variability in the index test risk of bias or reference standard applicability domains, these two were not included as potential moderators.

**Table 4:** QUADAS-2 quality assessment of included studies

Study	Risk of Bias				Applicability Concerns	
	Patient Selection	Index Test	Reference Standard	Flow & Timing	Patient Selection	Reference Standard
Apple et al. (2017)	H	L	L	L	L	L
Avci et al. (2017)	H	L	L	L	L	L
Barton (2021)	U	L	U	H	L	L
Blank et al. (2020)	H	L	U	U	L	L
Brewster et al. (2022)	U	L	U	L	H	L
Carlozzi et al. (2017)	U	L	L	H	L	L
Casaletto (2016)	H	L	L	L	H	H
Cassetta et al. (2020)	U	L	L	H	L	L
Chen et al. (2020)	U	L	L	L	L	L
Clausen et al. (2021)	U	L	L	L	L	L
Cohen (2019)	H	L	U	U	L	L
Davis et al. (2019)	H	L	L	U	L	L
de Silva et al. (2021)	L	L	L	H	L	L
Downes et al. (2022)	L	L	L	L	L	L
Dudley-Javoroski et al. (2022)	H	H	U	U	L	U
Dunbar et al. (2019)	U	L	L	L	H	L
Dutta (2020)	H	L	L	L	L	L
Elias et al. (2022)	H	L	L	H	H	H
Eneva et al. (2017)	H	L	L	L	H	L
Engel (2018)	H	L	H	U	L	L
Frazer et al. (2018)	H	L	L	L	L	L
French et al. (2021)	H	L	L	L	L	L
Gills et al. (2021)	H	L	U	L	L	L
Gladstone et al. (2019)	H	L	U	U	L	L
Hackett et al. (2018)	U	L	L	H	L	L
Hartmann et al. (2018)	H	L	L	U	H	L
Hawkins et al. (2021a)	H	L	L	L	L	L
Hawkins et al. (2021b)	H	L	L	L	L	L
Henry (2020)	H	L	U	L	H	H
Study	Risk of Bias				Applicability Concerns	
	Patient Selection	Index Test	Reference Standard	Flow & Timing	Patient Selection	Reference Standard
Hoffman et al. (2021)	U	L	U	H	L	L
Hollister (2015)	U	L	U	L	L	L
Hoover et al. (2019)	H	L	U	U	H	L
Hwang et al. (2019)	L	L	L	L	H	L
Kaat et al. (2022)	U	L	U	H	L	L
Kalyani et al. (2019)	H	L	U	U	L	L
Kamerer et al. (2019)	U	L	L	L	L	L
Karawani et al. (2018)	H	L	L	L	L	L
Kim et al. (2021)	L	L	L	H	L	L
Koopowitz et al. (2021)	U	L	L	L	H	L
Kratz et al. (2020)	H	L	U	U	L	L
Kringle (2019)	H	L	U	L	H	L
Kunker et al. (2020)	U	L	U	U	L	L
Ludyga et al. (2021)	H	L	L	H	L	L
Lundine et al. (2018)	U	H	L	L	L	L
Maas et al. (2021)	H	L	L	H	L	L
Marinac et al. (2019)	H	L	U	U	L	L
Mazzoli et al. (2021)	H	L	U	U	L	L
MacIsaac (2018)	H	L	U	H	L	L
Meier et al. (2022)	U	L	U	H	L	L
Meredith et al. (2020)	H	L	L	L	L	L
Modi et al. (2018)	U	L	L	U	L	L
Molinaro et al. (2021)	U	L	L	L	L	L
Moore et al. (2020)	U	L	L	H	L	L
Moriarty (2019)	U	L	U	U	L	L
Morrow et al. (2020)	U	L	U	H	L	L
Mulhauser et al. (2021)	U	L	L	L	L	L
Norman et al. (2019)	U	L	U	U	L	L
Paolillio (2021)	L	L	L	L	L	L
Papalambros et al. (2019)	U	L	L	U	L	L
Pardej (2020)	H	L	U	H	L	L
Pozar et al. (2020)	H	L	U	L	H	L
Study	Risk of Bias				Applicability Concerns	
	Reference Standard	Index Test	Reference Standard	Flow & Timing	Patient Selection	Reference Standard
Prussien et al. (2021)	U	L	L	U	L	L
Read et al. (2020)	H	L	L	L	L	L
Rebchuck et al. (2022)	H	L	L	U	L	L
Richerson et al. (2021)	H	L	L	H	L	L
Rockhold et al. (2021)	U	L	L	L	L	L
Russell-Schulz et al. (2021)	U	L	U	H	L	L
Sanborn et al. (2022)	H	L	U	U	L	L
Schmithorst et al. (2022)	H	L	U	U	L	L
Scholl et al. (2021)	U	L	U	L	U	L
Shapiro et al. (2021)	L	L	L	H	L	L
Shiau et al. (2021)	H	U	L	U	H	L
Siciliano et al. (2021)	L	L	L	U	L	L
Siciliano et al. (2022)	L	L	L	H	L	L
Sinha et al. (2018)	L	L	L	L	L	L
Slack (2018)	U	L	L	U	L	L
Solomon et al. (2021)	H	L	L	U	L	L
Terry et al. (2019)	L	L	L	H	L	L
Thomas et al. (2021)	U	U	L	U	U	L
Thompson et al. (2020)	U	L	L	U	L	L
Wakaizumi et al. (2021)	U	U	U	U	L	L
Watson et al. (2020)	H	L	L	H	L	L
Zimmerman et al. (2019)	H	L	L	U	L	L
Zuniga & Moran (2018)	H	L	U	L	L	L

## Publication Bias

### Comparisons with NIHTB-CB Normative Sample

The Egger’s tests were non-significant for the main effects within the clinical samples. Of the eight significant main effects for clinical samples, there were two significant trim and fill analyses that required values to be added to create a symmetrical funnel plot: episodic memory (PSM) and oral reading (ORR). The bias-adjusted effects are included in Table 2. After adjusting for bias, the ORR effect remained significant although PSM was no longer significant. As noted above, publication bias was assessed only when there were at least 11 studies (Higgins & Green, 2011). Given the small sample of studies for most clinical subgroups, publication bias could only be evaluated for ABI. Of the seven significant main effects for patients with an ABI, three fluid cognitive domains required values to be added to create a symmetrical funnel plot: inhibitory control (FICA), working memory (LSWM), and episodic memory (PSM). After adjusting for bias, the FICA effect remained significant although LSWM and PSM were no longer significant.

### Comparisons with Recruited Comparison Samples

None of the Egger’s tests yielded a significant result across the clinical samples. Furthermore, visual inspection of the funnel plots did not reveal any asymmetry among the data. Of the nine significant main effects, only one fluid cognitive domain (i.e., episodic memory; PSM) required values to be added to create a symmetrical funnel plot. However, it remained significant after adjusting for bias. There were not enough studies to examine publication bias for any clinical subgroup compared to recruited comparison samples.

## Main Effects

### Comparisons with NIHTB-CB Normative Sample

#### Clinical Samples

As hypothesized, the clinical samples performed significantly worse on the FCC (*g* = − 0.58, a medium effect), and there was no performance difference on the CCC compared to the NIHTB-CB normative sample (see Table 2; Fig. 2). Further, there was no significant performance difference on the CFC. On the NIHTB-CB subtests, the clinical samples performed significantly worse on four of the five fluid cognition subtests: LSWM, DCCS, FICA, and PCPS (*g*’s range from small to large, − 0.24 to − 0.80). There was no performance difference on PSM after adjusting for publication bias. Contrary to our hypothesis, clinical samples performed significantly better compared to the NIHTB-CB normative sample on the two crystallized cognition subtests: PV and ORR (*g*’s = 0.23 and 0.27, respectively; small effects).

#### ABI

Patients with an ABI performed significantly worse on the FCC (*g* = − 0.58). After adjusting for publication bias, patients with an ABI performed significantly worse on inhibitory control and attention (FICA; *g* = − 0.75), cognitive flexibility (DCCS; *g* = − 0.65), and processing speed (PCPS; *g* = − 0.92). After adjusting for publication bias, there were no performance differences in working memory or episodic memory. Contrary to expectation, patients performed better than normative data on the crystallized cognition composite (CCC; *g* = 0.30), although there were no performance differences on the crystallized cognition subtests.

#### Cancer and Tumor

There were no significant group differences on the CFC or FCC. Patients performed significantly worse on inhibitory control and attention (FICA; *g* = − 0.43) and processing speed (PCPS; *g* = − 0.58); there was no difference on cognitive flexibility and episodic memory. Contrary to expectations, patients with cancer or tumors performed better on working memory (LSWM; *g* = 0.24, small effect), oral reading recognition (ORR; *g* = 0.89, large effect), and the crystallized cognition composite (CCC; *g* = 0.68, medium effect).

#### Epilepsy

There were three studies that included patients with epilepsy and administered four fluid cognition subtests. Patients with epilepsy performed significantly worse than normative data on the working memory (LSWM; *g* = − 0.66) and cognitive flexibility (DCCS; *g* = − 0.86) subtests; no differences were found on measures of inhibitory control and attention or processing speed.

#### Hearing Loss

There were three studies that included patients with hearing loss that administered the FICA and PCPS subtests; no significant differences in cognitive performance were reported.

#### HIV

Three studies included patients with HIV and administered four fluid cognition subtests. Patients with HIV performed significantly worse on a measure of inhibitory control and attention (FICA; *g* = − 0.94) and working memory (LSWM; *g* = − 0.64); no performance differences were found on the cognitive flexibility or processing speed.

#### Metabolic and Vascular

There were enough studies of metabolic and vascular conditions to calculate effect sizes for the five fluid cognition subtests, fluid cognition composite, and one crystallized cognition subtest. A significant performance difference was found only on a measure of inhibitory control and attention (FICA; *g* = − 0.95) such that patients with a metabolic or vascular disease performed significantly worse than normative data.

#### Neurodegenerative Disorders

There were enough studies that included patients with a neurodegenerative disorder to calculate effects for the five fluid cognition subtests and two crystallized cognition subtests. Patients with a neurodegenerative disorder performed significantly worse on inhibitory control and attention (FICA; *g* = − 0.94), processing speed (PCPS; *g* = − 1.01), and episodic memory (PSM; *g* = − 0.45). There were no performance differences on measures of working memory or cognitive flexibility. Unexpectedly, patients with a neurodegenerative disease performed better than the normative data on the crystallized cognition subtests: receptive vocabulary (PV; *g* = 0.38) and oral reading (ORR; *g* = 0.60).

#### Neurodevelopmental Disorders

There were enough studies that included individuals with a neurodevelopmental disorder to calculate effects for all NIHTB-CB subtests and composites. There were performance differences on the composite of fluid cognition abilities (FCC; *g* = − 1.52) and three of the five associated subtests: inhibitory control (FICA; *g* = − 0.70), working memory (LSWM; *g* = − 0.56), and processing speed (PCPS; *g* = − 0.82). There were no performance differences on the total cognition composite, crystallized cognition measures, or subtests assessing episodic memory or cognitive flexibility.

#### Pain Syndromes

There were three studies that includes patients with pain syndromes and administered four fluid cognition subtests. Patients with a pain syndrome performed significantly worse on a measure of inhibitory control and attention (FICA; *g* = − 1.10) and cognitive flexibility (DCCS; *g* = − 0.22); there were no differences on working memory or processing speed.

#### Substance Use Disorders

There were four studies that included patients with a substance use disorder and administered the five fluid cognition subtests and one crystallized cognition subtest. Patients with a substance use disorder performed significantly worse on inhibitory control and attention (FICA: *g* = − 0.66), cognitive flexibility (DCCS: *g* = − 0.49), and processing speed (PCPS: *g* = − 0.45); unexpectedly, patients performed significantly better on a measure of reading recognition compared to normative data (ORR: *g* = 0.76).

### Comparisons with Recruited Comparison Samples

#### Clinical Samples

Consistent with our hypothesis, the clinical samples performed significantly worse on the composite of fluid cognition (*g* = − 0.86, large effect; see Table 3 and Fig. 2). Further, the clinical samples performed significantly worse on all five fluid cognition subtests, even after adjusting for publication bias on PSM. Contrary to our hypothesis, the clinical samples performed worse on the composite of crystallized cognition (*g* = − 0.37, small effect) and on the receptive vocabulary crystallized cognition subtest (PV; *g* = − 0.35, small effect). There was no performance difference on the oral reading subtest (ORR). 

### Moderators

The heterogeneity statistics for clinical samples in Table 2 indicate that there is significant variability across studies and suggest that a considerable proportion of the observed variance for the effects that may reflect methodological and clinical diversity among the included studies (*I*^2^ range: 84.39–94.79%; Higgins & Green, 2011). Therefore, six potential moderators were explored as contributors to the between group heterogeneity for the clinical samples and results are presented in Tables S3 and S4 and summarized below.

#### Study Quality

Since a numerical study quality score is not recommended (Whiting et al., 2011), groups were categorized into low, high, and unclear risk of bias for each QUADAS-2 domain. Overall, there was minimal evidence that risk of bias or applicability concerns were associated with effect size estimates except for the flow and timing domain (see Table S4). Results indicated that participants performed significantly worse on the CFC, FCC, and LSWM than the normative sample when studies were rated as high risk of bias. In contrast, participants performed better on two crystallized cognition measures (CCC and PV) than the normative data when studies were rated as low risk of bias.

#### Test Version

The effect of the NIHTB-CB test version (i.e., V1 desktop or V2 iPad) was examined as a potential moderator. As hypothesized, there were significant differences in performance on the FICA depending on which test version participants completed (*Q* = 10.25, *df* = 1, *p* = 0.001). Specifically, those who completed the FICA on the V2 iPad scored significantly lower when compared to the normative sample (*k* = 35, *g* = − 0.99, SE = 0.09, *Z* = − 11.73, *p* < 0.001) than those who completed it on the V1 desktop (*k* = 17, *g* = − 0.50, SE = 0.13, *Z* = − 3.90, *p* < 0.01).

#### Age

**Fig. 3:**
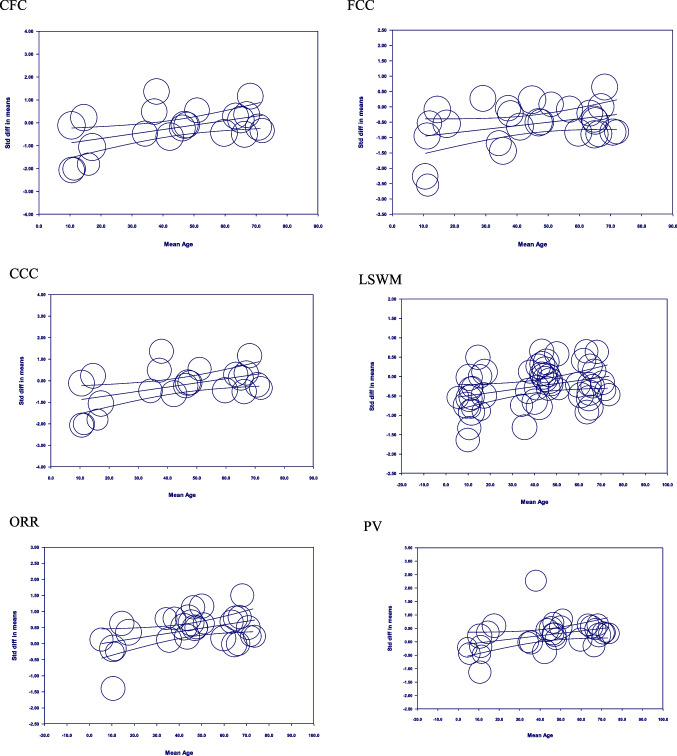
Significant moderation effect of age on NIHTB-CB scores in clinical samples. *CFC* cognitive function composite, *FCC* fluid cognition composite, *CCC* crystallized cognition composite, *LSWM* list sorting working memory, *ORR* oral reading recognition, *PV* picture vocabulary

Mean participant age was a significant moderator of effects on several measures of cognitive functioning such that studies that included older participants tended to perform better on cognitive measures compared to the NIHTB-CB normative data than those who included younger participants with a clinical condition (see Fig. 3 and Table S3). Specifically, age significantly moderated the effect for all three cognition composite scores: CFC (*k* = 22, *β* = 0.02, SE = 0.01, Z = 2.93, *p* < 0.01), FCC (*k* = 27, *β* = 0.01, SE = 0.01, Z = 1.98, *p* < 0.05), and CCC (*k* = 21, *β* = 0.01, SE = 0.01, Z = 2.21, *p* < 0.05). Further, age moderated the effect for both crystallized cognition subtests (ORR: *k* = 28, *β* = 0.01, SE = 0.00, Z = 2.56, *p* < 0.05 and PV: *k* = 30, *β* = 0.01, SE = 0.00, Z = 2.16, *p* < 0.05) and the LSWM fluid cognition subtest (*k* = 53, *β* = 0.01, SE = 0.00, Z = 2.46, *p* < 0.05).

#### Sex

**Fig. 4:**
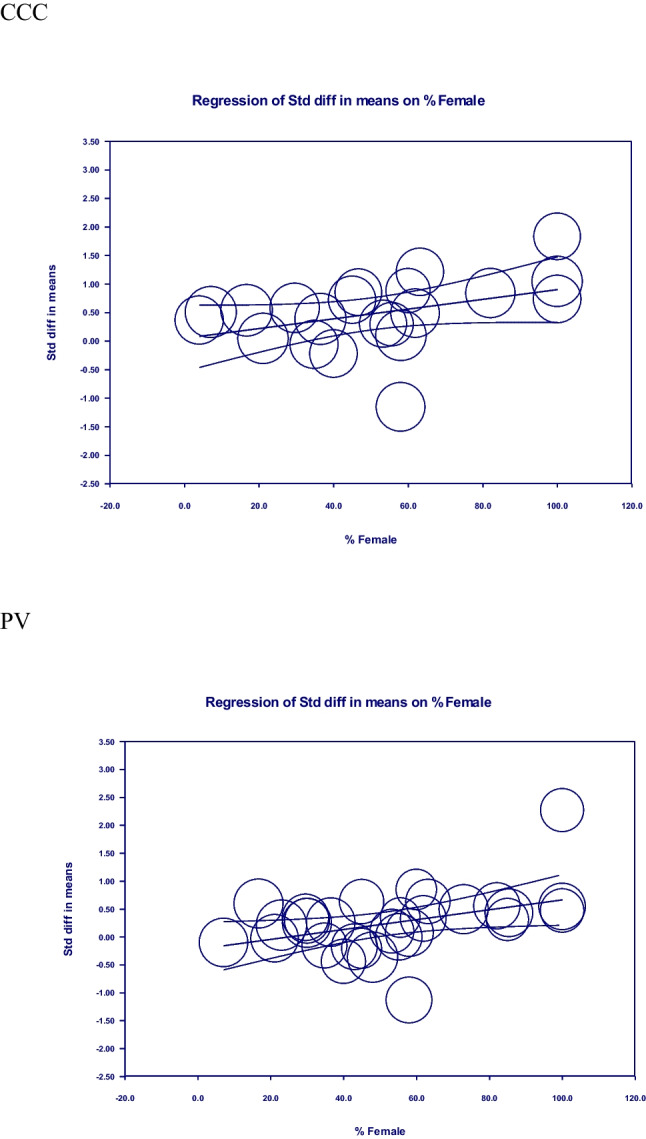
Significant moderation effect of sex on NIHTB-CB scores in clinical samples

Participant sex was examined as a moderator by analyzing the percentage of females in the sample, and two of the three crystallized cognition measures were moderated by sex such that studies with a higher percentage of female participants tended to perform better on the crystallized cognition subtests compared to the normative data (see Fig. 4 and Table S3). Specifically, participant sex significantly moderated the effect of the CCC (*k* = 21, *β* = 0.01, SE = 0.00, Z = 2.07, *p* < 0.05) and the PV subtest (*k* = 29, *β* = 0.01, SE = 0.00, Z = 2.59, *p* < 0.05).

#### Education

**Fig. 5:**
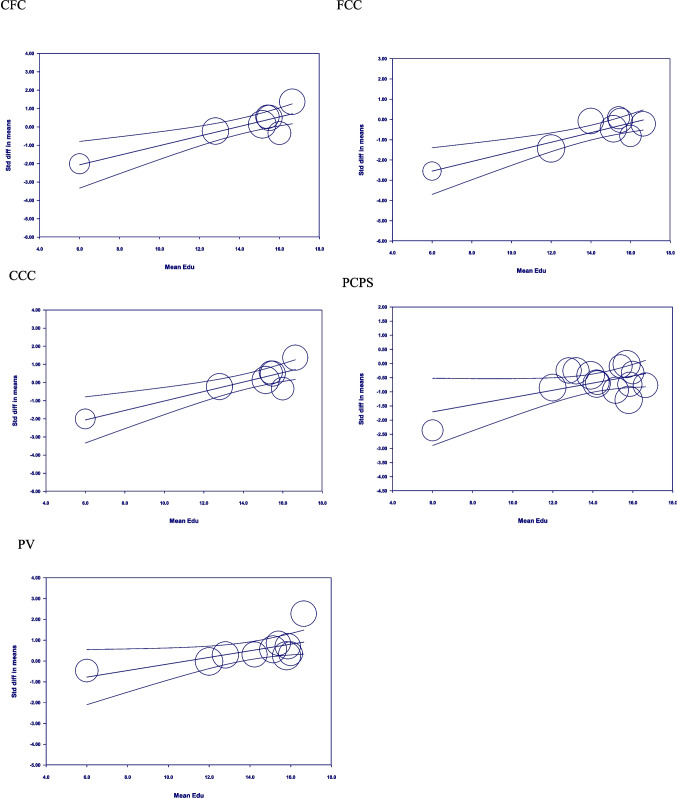
Significant moderation effect of education on NIHTB-CB scores in clinical samples

The effect of participant education was examined as a potential moderator by using the mean level of education in the sample for the studies that reported this information. Several cognitive domains were significantly moderated by participant education such that studies with a higher mean education level tended to perform better on both crystallized and fluid cognition measures (see Table S3; Fig. 5). Specifically, education significantly moderated the effect for all three composite scores: CFC (*k* = 7, *β* = 0.26, SE = 0.06, Z = 4.42, *p* < 0.01), FCC (*k* = 8, *β* = 0.24, SE = 0.05, Z = 4.33, *p* < 0.01), and CCC (*k* = 8, *β* = 0.22, SE = 0.05, Z = 4.56, *p* < 0.01). Further, education moderated the effect for one crystallized cognition subtest (PV: *k* = 10, *β* = 0.16, SE = 0.06, Z = 2.51, *p* < 0.05) and one fluid cognition subtest (PCPS: *k* = 14, *β* = 0.13, SE = 0.06, Z = 2.23, *p* < 0.05).

#### Publication Type

The effect of publication type (i.e., thesis or peer-reviewed journal article) was examined as a potential moderator in clinical samples (see Table S4). There was a significant difference on FICA (*Q* = 4.61, *df* = 1, *p* = 0.03), with participants included in peer-reviewed journal articles (*g* = − 0.86, SE = 0.07, *Z* = − 12.02, *p* < 0.001) performing significantly worse on this subtest than participants included in the theses (*g* = − 0.49, SE = 0.16, *Z* = − 3.08, *p* < 0.01).

## Discussion

The current study provides the first quantitative review of research with clinical samples using the English version of the NIHTB-CB across the lifespan. Building on the scoping review of Fox et al. (2022) which reported on the extent to which the NIHTB measures have been used with clinical populations, the primary goal was to examine the utility of the NIHTB-CB in identifying established differences in cognitive performance in research with clinical samples. It is evident from this meta-analytic review that there is a large body of research using the NIHTB-CB in clinical samples, as reflected in the 84 published and unpublished studies included from our literature search with 6331 clinical participants. This meta-analysis identified several key findings. First, there is clear evidence that the NIHTB-CB is sensitive to detecting differences in fluid cognitive skills in clinical samples when compared to both the NIHTB-CB normative sample and recruited comparison samples. Second, unexpectedly, the evidence for group differences in crystallized cognitive skills was mixed depending on the comparison sample. Third, there was minimal evidence for publication bias in studies comparing cognitive performance to the normative data or recruited comparison samples. Lastly, there was some evidence for moderator effects of study quality and participant demographics. Each of these findings will be discussed in turn.

### Fluid Cognition Composite and Subtests

As hypothesized, there was clear evidence for significantly poorer performance by clinical samples compared with the established NIHTB-CB normative data on the fluid cognition measures, which assess the skills needed to quickly and efficiently process and encode new information and solve novel problems. These abilities are particularly important for learning and adapting to new environmental demands and have consistently been shown to be impaired in a wide range of clinical populations (e.g., Abramovitch et al., 2019; Bernstein et al., 2017; Catalan et al., 2021; Moran, 2016; Naguib et al., 2009; Prussien et al., 2019). On the five fluid cognition subtests, clinical samples consistently performed worse on measures of processing speed, inhibitory control and attention, working memory, and cognitive flexibility regardless of the comparison group with most effect sizes medium to large in magnitude. The findings for impairments in episodic memory (i.e., PSM) were mixed depending on the comparison sample. Clinical samples performed worse on episodic memory compared to recruited controls, but performance differences were non-significant compared to the normative sample. It is notable that the PSM subtest was most frequently impacted by publication bias, and so there may be selective reporting for this subtest that contributes to inconsistencies.

Although there was significant heterogeneity in the clinical samples identified in the review of the use of the NIHTB-CB, there were ten narrower disease groups with sufficient studies (*k* = 3) to conduct subgroup analyses. The findings suggest variability in the cognitive performance across the various clinical conditions, but several patterns emerged that align well with prior research. Patients with ABI and substance use disorders demonstrated significant deficits in inhibitory control and attention, cognitive flexibility, and processing speed (e.g., Arciniegas et al., 2002; Keyes et al., 2017). For patients with cancer and neurodegenerative diseases, impairments were observed in processing speed and inhibitory control skills, which aligns with previous research demonstrating executive dysfunction and cognitive slowing in these diseases (e.g., Bernstein et al., 2017; Verbaan et al., 2007). Consistent with prior research, patients with neurodevelopmental disorders demonstrated significant impairments in fluid cognition, including inhibitory control, working memory, and processing speed (e.g., Corbett et al., 2009). Patients with HIV demonstrated marked deficits in working memory and inhibitory control and attention, reflecting known cognitive impairments in HIV (e.g., Walker & Brown, 2018). Metabolic and vascular diseases demonstrated impairment in inhibitory control, a common deficit established in previous research (e.g., Naguib et al., 2009). There was evidence that patients with a pain syndrome demonstrated significant impairments in inhibitory control and cognitive flexibility while patients with epilepsy demonstrated difficulties in cognitive flexibility and working memory, consistent with documented difficulties in these disorders (e.g., Berryman et al., 2014; Novak et al., 2022). Finally, there were no identified differences in cognitive performance for individuals with hearing loss. It is important to acknowledge that many sub-groups included a small number of studies (*k* < 8), limiting the power to detect significant effects. Therefore, while many of the findings provide evidence that the NIHTB-CB detects known cognitive deficits in a range of populations, these sub-group analyses should be interpreted with some caution.

### Crystallized Cognition Composite and Subtests

As expected, there was not a significant effect for clinical samples on the composite of crystallized cognitive skills, which assesses accumulated verbal knowledge and is more heavily influenced by one’s prior learning experiences and environment than fluid cognition. Research has shown that crystallized cognition abilities tend to be preserved in clinical populations (e.g., King et al., 2023; McDonough et al., 2016; Tulsky et al., 2017). Unexpectedly, there was some evidence that clinical samples performed significantly better on the crystallized cognition subtests compared to the NIHTB-CB normative sample with small effects after adjusting for publication bias. Further, the performance of the five clinical subgroups that administered crystallized cognition measures was consistently higher than the normative sample, with most effect sizes ranging from medium to large. In contrast, clinical participants consistently performed *worse* on crystallized cognition measures compared to recruited controls.

Overall, these unexpected and inconsistent findings raise concerns about the validity of the crystallized cognition data obtained from the NIHTB-CB in clinical research. There are several potential explanations for these inconsistent findings. First, this review revealed that a smaller body of research has examined crystallized cognition compared to fluid cognition in clinical populations. Further, one of the two crystallized cognition subtests was affected by publication bias, and so it is possible that significant effects were more likely to be published. Second, the NIHTB-CB normative data on the crystallized cognition tests may not accurately represent the general population and may overestimate skills. Third, the study participants may not have been representative of the clinical population, leading to skewed findings. There is evidence that patients with higher levels of education and fewer cognitive and environmental barriers and are more likely to participate in clinical research (e.g., Baquet et al., 2008). Given that crystallized cognition is influenced by environmental factors and participant demographics significantly moderated the crystallized cognition effects, this discrepancy in performance depending on the comparison sample underscores the importance of recruiting a matched comparison group in clinical research.

### Cognitive Function Composite

The findings were mixed regarding performance differences on the CFC, which is a composite of the FCC and CCC subtests. There was no difference in performance on CFC for clinical samples compared to the NIHTB-CB normative sample, but the clinical samples performed significantly *lower* on the CFC when compared with recruited comparison samples. Although 57% of experts rated a total cognitive score as important in the development of the NIHTB-CB (Weintraub et al., 2013a, 2013b), the variability in performance across studies raises questions about the utility of this composite with clinical samples.

### Publication Bias

There was some evidence for publication bias in the main effect analyses comparing clinical samples to the NIHTB-CB population normative sample. Overall, there were five significant trim and fill analyses. Notably, the measure of episodic memory (i.e., PSM) was consistently identified as demonstrating publication bias. After adjusting for bias, two of the three PSM effects were no longer significant, suggesting that performance deficits on this measure are consistently overestimated for clinical samples. Regarding clinical subgroup analyses, there were only enough studies in ABI to examine publication bias. However, there was evidence for publication bias on three fluid cognition measures, with two of the three effects no longer significant after adjusting for the bias. Taken together, publication bias findings highlight a critical issue in research on cognitive functioning as selective reporting can distort the presence of cognitive impairments in clinical populations. It is critical that both significant and non-significant findings are published to maximize transparency in clinical research. However, to further minimize publication bias, we contacted corresponding authors and included, when possible, any of their unpublished data.

### Moderator Analyses

The high between-study heterogeneity suggests there was methodological and clinical diversity in the included studies, and so six potential moderators were examined to try and account for some of the heterogeneity.

#### Study Quality

There was minimal evidence that the quality of the study impacted the strength of the effects except for bias noted in the flow and timing domain. Specifically, participants from studies that were rated as high risk of bias in flow and timing performed significantly worse on the CFC, FCC, and LSWM. In contrast, participants performed better on the two crystallized cognition subtests when studies were rated as low risk of bias. In the present review, the two most common reasons for the study being rated as “high risk of bias” included (1) patients receiving different reference standards to establish the clinical diagnosis, or (2) fewer than 90% of the enrolled participants were included in the statistical analysis. Verification bias may be introduced when patients are diagnosed with the clinical condition using different methods and standards, as patients may be inconsistently classified (Whiting et al., 2011). Additionally, missing data can introduce potential bias by reducing statistical power and reducing the representativeness of the sample, particularly if the data are not missing at random (Kang, 2013).

#### Test Version

Consistent with the findings by Brearly and colleagues (2019), performance on the FICA subtest was moderated by test version, such that clinical samples performed significantly worse compared to normative samples when the subtest was administered with V2 on the iPad compared to V1 on the desktop computer. Although the reason for the performance difference is unclear, Brearly et al. (2019) hypothesized that the performance difference may be driven by differences in the display of stimuli since the FICA depends upon discernment between small visual details. There were no other moderator effects of test version. Nevertheless, given that the findings with the FICA were replicated in this meta-analysis, it is timely that V3 of the Toolbox was recently released with a new normative sample of 3904 participants, ages 3 and older, that was representative of the US population based on age, biological sex, race/ethnicity, and education (Hook & Giella, 2023). Studies using V3 of the Toolbox will be important in resolving the disparities in findings on V1 and V2 on the FICA.

#### Age

Several age effects were found in these analyses. First, in clinical samples, younger individuals performed significantly worse on the FCC and CFC scores as well as the LSWM subtest when compared to the NIHTB-CB normative sample. While this gap in performance had closed by the 6th decade of life for the CFC and LSWM, differences in fluid cognition remained into late adulthood. These observed age effects may be due to two possible factors. First, it is possible that fluid cognition is more sensitive to effects of disease and treatment in younger children (e.g., Compas et al., 2017). Alternatively, it is possible that the NIHTB-CB is more sensitive to detecting differences in cognitive performance in younger age groups given the much larger cohort of children compared to adults in the original NIHTB-CB normative sample (Carlozzi et al., 2015). Regarding crystallized cognition, performance of clinical samples was comparable to the normative sample in children and then increased with age such that older adults were outperforming the normative data. While prior research demonstrates improvements in language and crystallized intelligence with age through middle adulthood (Weintraub et al., 2013b), given that the data included in the meta-analysis were age-corrected, effects for age were unexpected and suggest that the NIHTB-CB scores may overestimate crystallized cognition at older ages. Despite these observed differences as a function of age, it is important to note that the data included were cross-sectional in nature, and so changes should not be seen as longitudinal changes within individual participants, but rather a projection of functioning across the lifespan using the NIHTB-CB in diverse clinical populations. Furthermore, it is outside the scope of this review to explain mechanisms underlying cognitive dysfunction throughout the age span given the marked heterogeneity of the groups included in clinical samples.

#### Sex

Participant sex differences were identified on measures of crystallized cognition (see Fig. 4). Specifically, as the percentage of females in the sample increased, performance in two crystallized cognition measures also increased. This finding is in keeping with the effort undertaken by Casaletto and colleagues (2015) to calculate Fully Corrected *T* scores, who found a small, but significant impact of sex on cognitive performance. It is notable that seven of the included clinical samples included all-female cohorts with breast cancer or a history of breast cancer. However, the literature on cognitive outcomes in breast cancer survivors does not fully account for the patterns demonstrated in the present moderator analysis (e.g., Jim et al., 2012).

#### Education

Several cognitive domains were significantly moderated by participant education such that studies with a higher mean level of education tended to perform better on both crystallized and fluid cognition measures (see Fig. 5). These findings are consistent with a large body of literature that has shown a strong link between education and cognitive function (e.g., Lovden et al., 2020). While only 30% of the included studies reported mean level of participant education, the significant role of education highlights the importance of providing it in empirical studies and recognizing its role in cognitive performance. Notably, the Fully Corrected *T* scores computed by the NIHTB-CB account for participant demographic factors, including education. However, given the limited number of studies that reported participant education in the current review, we were unable to examine if the role of education was different in the Age-Corrected versus Fully Corrected scores computed by the NIHTB-CB.

#### Publication Type

Across all ten subtest and composite scores, publication type was a significant moderator for only the FICA subtest such that participants performed worse compared to the normative sample in peer-reviewed journal articles compared to unpublished theses. While this suggests there may be some publication bias on the FICA, it is promising that the remaining nine cognitive domains were non-significant. Furthermore, this underscores the importance of including unpublished literature in meta-analyses, which is a strength of our study.

### Strengths and Limitations of Current Meta-Analysis

This review has several strengths. First, a sufficiently large body of research including both published and unpublished studies (*k* = 71 peer-reviewed articles, *k* = 13 theses) was identified to conduct a comprehensive meta-analysis of the cognitive performance of clinical samples as compared with the NIHTB-CB normative sample and recruited comparison samples. Second, we were able to examine the use of the NIHTB-CB with ten disease subgroups. Third, we were able to carefully examine the potential presence of publication bias in clinical samples. Lastly, we were able to test for possible moderator effects associated with study quality, the first and second versions of the test battery, the demographic characteristics of the participants, and publication type.

Several limitations of this review are also noteworthy. First, there was significant heterogeneity across studies in the clinical populations included and the severity of the clinical conditions that may limit the generalizability of the findings and the conclusions that can be drawn about the NIHTB-CB. Second, there were not enough longitudinal studies to examine how cognitive deficits might change with disease course or treatment, to examine true developmental effects, or to assess the fitness of the NIHTB-CB as a measure to assess changes in cognitive function over time. Third, V3 of the NIHTB-CB was released in early 2023 and no published studies were available to examine cognitive effects with this new version of the battery. Fourth, only studies that administered the English version of the NIHTB-CB were included; therefore, these results may not be generalizable to other language versions of the battery. Lastly, there are other variables that may influence cognitive performance that could not be sufficiently explored given the enormous heterogeneity in which these variables were described and assessed in the included studies, such as disease severity, performance validity, cultural factors, and socioeconomic factors.

### Summary and Implications

There are several important implications and take-home messages from the findings of this meta-analysis. First, the findings from the NIHTB-CB align with the existing literature by documenting impairments in fluid cognition in several clinical groups (Centers for Disease Control and Prevention, 2023; Compas et al., 2017). This pattern broadly held for comparisons to the NIHTB-CB normative sample as well as recruited non-clinical comparison samples. It is notable that there was some evidence of publication bias, particularly on the PSM. Overall, it appears that the NIHTB-CB can be used as an efficient and easy-to-use screening tool for fluid cognition in research with clinical populations.

Second, unexpectedly with regard to crystallized cognitive function, there was some evidence that participants in clinical samples scored *higher* than the NIHTB-CB normative sample but scored significantly *lower* than recruited control samples. This pattern of findings raises concerns about the use of the NIHTB-CB to measure crystallized cognitive function and suggests that scores may be inflated in clinical groups. At this time, more research is needed to understand these patterns. However, given crystallized cognition is significantly influenced by environmental factors and participation barriers can lead to sampling bias, it is recommended that researchers use a matched comparison sample to best understand the influence of clinical conditions on cognition.

Third, as noted above, there was not a re-norming of the data when the platform transitioned from the desktop version (V1) to the iPad version (V2). Consequently, problems have been identified with the FICA subtest as a measure of attention and inhibitory control (Brearly et al., 2019). Consistent with findings reported by Brearly et al., it appears that the scores obtained on the FICA from V2 are artificially low. As access to V2 remains available through year 2028 and it is anticipated research will continue to be published from V2 (e.g., Nashiro et al., 2023), it is recommended that researchers interpret performance on this measure with caution.

## Future Directions

The results of this meta-analysis are helpful in identifying several directions for future research with the NIHTB-CB. First, while there were a sufficient number of studies to calculate effect sizes for a small number of specific clinical conditions, it will be important to revisit this research on the NIHTB-CB in the future as the body of literature continues to grow. Second, the NIHTB-CB V3 was released in early 2023 and, to date, no studies have been published using this new version. For V3, as described above, new normative data were collected by NIH Toolbox-trained examiners. It will be important to determine if the new normative sample addresses concerns identified in this meta-analysis concerning the FICA subtest and the crystallized cognition domain. Third, future research is needed to continue to evaluate the construct validity of the NIHTB-CB by administering it along with other well-established measures of neurocognitive function to determine the degree of convergence with other gold-standard measures. A number of studies have shown that the NIHTB-CB compares favorably with gold standard measures of cognitive function (e.g., Kringle et al., 2023; Manglani et al., 2022; Mungas et al., 2014; Weintraub et al., 2013a, 2013b), while other studies have found poor correspondence with gold standard measures (Ott et al., 2022). Further research is needed to resolve these discrepant findings.

In conclusion, the NIHTB-CB is a promising battery for continued use in clinical research to screen for cognitive problems in medical and psychiatric populations. The meta-analytic results show that the NIHTB-CB is effective in identifying deficits in overall fluid cognition and in specific aspects of this domain of cognitive function (attentional control, shifting, processing speed) in a broad range of clinical samples of all ages. Given its ease of administration and scoring for participants across the lifespan, these findings indicate the NIHTB-CB is a useful tool to assess fluid cognition in clinical research.

## Supplementary Information

Below is the link to the electronic supplementary material.

ESM 1 (DOCX 254 KB)

## Data Availability

The data that support the findings of this study are available from the corresponding author upon reasonable request.

## References

[CR1] Abramovitch, A., McCormack, B., Brunner, D., Johnson, M., & Wofford, N. (2019). The impact of symptom severity on cognitive function in obsessive-compulsive disorder: A meta-analysis. *Clinical Psychology Review,**67*, 36–44. 10.1016/j.cpr.2018.09.003 30528984 10.1016/j.cpr.2018.09.003

[CR2] Ahles, T. A., & Root, J. C. (2018). Cognitive effects of cancer and cancer treatments. *Annual Review of Clinical Psychology*, *14*(1548–5951 (Electronic)), 425–451. 10.1146/annurev-clinpsy-050817-084903 10.1146/annurev-clinpsy-050817-084903 PMC9118140 29345974

[CR3] American Psychiatric Association. (2013). *Diagnostic and Statistical Manual of Mental Disorders* (5th ed.). 10.1176/appi.books.9780890425596

[CR4] Apple, A. C., Ryals, A. J., Alpert, K. I., Wagner, L. I., Shih, P. A., Dokucu, M., Cella, D., Penedo, F. J., Voss, J. L., & Wang, L. (2017). Subtle hippocampal deformities in breast cancer survivors with reduced episodic memory and self-reported cognitive concerns. *Neuroimage Clinical,**14*, 685–691. 10.1016/j.nicl.2017.03.004 28377882 10.1016/j.nicl.2017.03.004 PMC5369871

[CR5] Arciniegas, D. B., Held, K., & Wagner, P. (2002). Cognitive impairment following traumatic brain injury. *Current Treatment Options in Neurology,**4*, 43–57. 11734103 10.1007/s11940-002-0004-6

[CR6] Avci, G., Woods, S. P., Verduzco, M., Sheppard, D. P., Sumowski, J. F., Chiaravalloti, N. D., DeLuca, J., & Group, H. I. V. N. R. P. (2017). Effect of retrieval practice on short-term and long-term retention in HIV+ individuals. *Journal of the International Neuropsychological Society,**23*(3), 214–222. 10.1017/S1355617716001089 28067192 10.1017/S1355617716001089 PMC5453504

[CR7] Babikian, T., & Asarnow, R. (2009). Neurocognitive outcomes and recovery after pediatric TBI: Meta-analytic review of the literature. *Neuropsychology,**23*(3), 283–296. 10.1037/a0015268 19413443 10.1037/a0015268 PMC4064005

[CR8] Bajpai, S., Upadhayay, A. D., Banerjee, J., Chakrawarthy, A., Chatterjee, P., Lee, J., & Dey, A. B. (2022). Discrepancy in fluid and crystallized intelligence: An early cognitive marker of dementia from the LASI-DAD cohort. *Dementia and Geriatric Cognitive Disorders Extra,**12*(1), 51–59. 10.1159/000520879 35611146 10.1159/000520879 PMC9082145

[CR9] * Barton, H. L. (2021). An examination of factors impacting executive functioning in children with developmental delays [Doctoral dissertation, University of Oregon].

[CR10] Bauer, P. J., Dikmen, S. S., Heaton, R. K., Mungas, D., Slotkin, J., & Beaumont, J. L. (2013). NIH toolbox cognition battery (CB): Measuring episodic memory. *Monographs of the Society for Research in Child Development*, *78*(4), 34–48. http://www.jstor.org.ezproxy.lib.utah.edu/stable/43772789 10.1111/mono.12033 PMC3950959 23952201

[CR11] Bauer, P. J., & Zelazo, P. D. (2013). NIH toolbox cognition battery (CB): Summary, conclusions, and implications for cognitive development. *Monographs of the Society for Research in Child Development*, *78*(4), 133–146. http://www.jstor.org.ezproxy.lib.utah.edu/stable/43772795 10.1111/mono.12039 23952207

[CR12] Bauer, P. J., & Zelazo, P. D. (2014). The National Institutes of Health Toolbox for the Assessment of Neurological and Behavioral Function: A tool for developmental science. *Child Development Perspectives,**8*(3), 119–124. 10.1111/cdep.12080

[CR13] Baquet, C. R., Henderson, K., Commiskey, P., & Morrow, J. N. (2008). Clinical trials: The art of enrollment. *Seminars in Oncology Nursing,**24*(4), 262–269. 10.1016/j.soncn.2008.08.006 19000600 10.1016/j.soncn.2008.08.006 PMC3262589

[CR14] Beaumont, J. L., Havlik, R., Cook, K. F., Hays, R. D., Wallner-Allen, K., Korper, S. P., Lai, J. S., Nord, C., Zill, N., Choi, S., Yost, K. J., Ustsinovich, V., Brouwers, P., Hoffman, H. J., & Gershon, R. (2013). Norming plans for the NIH Toolbox. *Neurology,**80*(11 Suppl 3), S87-92. 10.1212/WNL.0b013e3182872e70 23479550 10.1212/WNL.0b013e3182872e70 PMC3662345

[CR15] Bernstein, L. J., McCreath, G. A., Komeylian, Z., & Rich, J. B. (2017). Cognitive impairment in breast cancer survivors treated with chemotherapy depends on control group type and cognitive domains assessed: A multilevel meta-analysis. *Neuroscience and Biobehavioral Reviews,**83*, 417–428. 10.1016/j.neubiorev.2017.10.028 29092778 10.1016/j.neubiorev.2017.10.028

[CR16] Berryman, C., Stanton, T. R., Bowering, K. J., Tabor, A., McFarlane, A., & Moseley, G. L. (2014). Do people with chronic pain have impaired executive function? *A Meta-Analytical Review. Clinical Psychology Review,**34*(7), 563–579. 10.1016/j.cpr.2014.08.003 25265056 10.1016/j.cpr.2014.08.003

[CR17] Blank, A., Frush Holt, R., Pisoni, D. B., & Kronenberger, W. G. (2020). Associations between parenting stress, language comprehension, and inhibitory control in children with hearing loss. *Journal of Speech, Language, and Hearing Research,**63*(1), 321–333. 10.1044/2019_JSLHR-19-00230 31940261 10.1044/2019_JSLHR-19-00230 PMC7213483

[CR18] Borenstein, M., Hedges, L., Higgins, J., & Rothstein, H. (2005). Comprehensive meta-analysis: A computer program for research synthesis. In Biostat.

[CR19] Brearly, T. W., Rowland, J. A., Martindale, S. L., Shura, R. D., Curry, D., & Taber, K. H. (2019). Comparability of iPad and web-based NIH toolbox cognitive battery administration in veterans. *Archives of Clinical Neuropsychology,**34*(4), 524–530. 10.1093/arclin/acy070 30260372 10.1093/arclin/acy070 PMC9586718

[CR20] Brewster, G. S., Molinari, V., McCrae, C., Beckstead, J., D’Aoust, R., & Rowe, M. (2022). Cognitive function and sleep in caregivers of persons living with dementia. *Western Journal of Nursing Research,**44*(3), 260–268. 10.1177/01939459211041163 34467789 10.1177/01939459211041163 PMC9112431

[CR21] Campbell, L. K., Scaduto, M., Sharp, W., Dufton, L., Van Slyke, D., Whitlock, J. A., & Compas, B. (2007). A meta-analysis of the neurocognitive sequelae of treatment for childhood acute lymphocytic leukemia. *Pediatric Blood & Cancer,**49*(1), 65–73. 10.1002/pbc.20860 16628558 10.1002/pbc.20860

[CR22] Carlozzi, N. E., Beaumont, J. L., Tulsky, D. S., & Gershon, R. C. (2015). The NIH Toolbox pattern comparison processing speed test: Normative data. *Archives of Clinical Neuropsychology,**30*(5), 359–368. 10.1093/arclin/acv031 26025230 10.1093/arclin/acv031 PMC4542749

[CR23] Carlozzi, N. E., Goodnight, S., Casaletto, K. B., Goldsmith, A., Heaton, R. K., Wong, A. W. K., Baum, C. M., Gershon, R., Heinemann, A. W., & Tulsky, D. S. (2017). Validation of the NIH toolbox in individuals with neurologic disorders. *Archives of Clinical Neuropsychology,**32*(5), 555–573. 10.1093/arclin/acx020 28334392 10.1093/arclin/acx020 PMC5860275

[CR24] Carlozzi, N. E., Tulsky, D. S., Kail, R. V., & Beaumont, J. L. (2013). NIH toolbox cognition battery (CB): Measuring processing speed. *Monographs of the Society for Research in Child Development*, *78*(4), 88–102. http://www.jstor.org.ezproxy.lib.utah.edu/stable/43772792 10.1111/mono.12036 PMC4425122 23952204

[CR25] (*) Casaletto, K. B. (2016). A metacognition-based approach to improve HIV-associated neurocognitive disorders among substance users [Doctoral dissertation, University of California, San Diego]. ProQuest.

[CR26] Casaletto, K. B., Umlauf, A., Beaumont, J., Gershon, R., Slotkin, J., Akshoomoff, N., & Heaton, R. K. (2015). Demographically corrected normative standards for the English version of the NIH toolbox cognition battery. *Journal of the International Neuropsychological Society,**21*(5), 378–391. 10.1017/S1355617715000351 26030001 10.1017/S1355617715000351 PMC4490030

[CR27] Cassetta, B. D., Menon, M., Carrion, P. B., Pearce, H., DeGraaf, A., Leonova, O., White, R. F., Stowe, R. M., Honer, W. G., Woodward, T. S., & Torres, I. J. (2020). Preliminary examination of the validity of the NIH toolbox cognition battery in treatment-resistant psychosis. *Clinical Neuropsychologist,**34*(5), 981–1003. 10.1080/13854046.2019.1694072 31782350 10.1080/13854046.2019.1694072

[CR28] Catalan, A., Salazar de Pablo, G., Aymerich, C., Damiani, S., Sordi, V., Radua, J., Oliver, D., McGuire, P., Giuliano, A. J., Stone, W. S., & Fusar-Poli, P. (2021). Neurocognitive functioning in individuals at clinical high risk for psychosis: A systematic review and meta-analysis. *JAMA Psychiatry,**78*(8), 859–867. 10.1001/jamapsychiatry.2021.1290 34132736 10.1001/jamapsychiatry.2021.1290 PMC8209603

[CR29] Cattell, R. B. (1971). *Abilities: Their structure, growth, and action*. Houghton Mifflin.

[CR30] * Chen, B. T., Ye, N., Wong, C. W., Patel, S. K., Jin, T., Sun, C. L., Rockne, R. C., Kim, H., Root, J. C., Saykin, A. J., Ahles, T. A., Holodny, A. I., Prakash, N., Mortimer, J., Sedrak, M. S., Waisman, J., Yuan, Y., Li, D., Vazquez, J., . . . Dale, W. (2020). Effects of chemotherapy on aging white matter microstructure: A longitudinal diffusion tensor imaging study. *Journal of Geriatric Oncology*, *11*(2), 290–296. 10.1016/j.jgo.2019.09.016 10.1016/j.jgo.2019.09.016 PMC7054164 31685415

[CR31] Centers for Disease Control and Prevention. *Chronic Diseases and Cognitive Decline - A Public Health Issue*. (2023). Retrieved November 2023 from https://www.cdc.gov/aging/publications/chronic-diseases-brief.html

[CR32] Clausen, A. N., Bouchard, H. C., Workgroup, V.A.M.-A.M., Welsh-Bohmer, K. A., & Morey, R. A. (2021). Assessment of neuropsychological function in veterans with blast-related mild traumatic brain injury and subconcussive blast exposure. *Frontiers in Psychology,**12*, Article 686330. 10.3389/fpsyg.2021.686330 34262512 10.3389/fpsyg.2021.686330 PMC8273541

[CR33] Cohen, J. (1988). Statistical power analysis for the behavioral sciences (2 ed.). Lawrence Erlbaum Associates.

[CR34] * Cohen, J. D. (2019). A Pilot study exploring the acute effects of aerobic exercise and relaxation on fatigue and executive function in breast cancer survivors [Doctoral dissertation, University of Illinois at Urbana-Champaign].

[CR35] Compas, B. E., Jaser, S. S., Reeslund, K., Patel, N., & Yarboi, J. (2017). Neurocognitive deficits in children with chronic health conditions. *American Psychologist,**72*(4), 326–338. 10.1037/amp0000042 28481580 10.1037/amp0000042 PMC7212494

[CR36] Corbett, B. A., Constantine, L. J., Hendren, R., Rocke, D., & Ozonoff, S. (2009). Examining executive functioning in children with autism spectrum disorder, attention deficit hyperactivity disorder and typical development. *Psychiatry Research,**166*(2–3), 210–222. 10.1016/j.psychres.2008.02.005 19285351 10.1016/j.psychres.2008.02.005 PMC2683039

[CR37] Crivelli, L., Palmer, K., Calandri, I., Guekht, A., Beghi, E., Carroll, W., Frontera, J., Garcia-Azorin, D., Westenberg, E., Winkler, A. S., Mangialasche, F., Allegri, R. F., & Kivipelto, M. (2022). Changes in cognitive functioning after COVID-19: A systematic review and meta-analysis. *Alzheimer’s & Dementia,**18*(5), 1047–1066. 10.1002/alz.12644 10.1002/alz.12644 PMC9073922 35297561

[CR38] Dalton, J. E., Bolen, S. D., & Mascha, E. J. (2016). Publication bias: The elephant in the review. *Anesthesia and Analgesia,**123*(4), 812–813. 10.1213/ANE.0000000000001596 27636569 10.1213/ANE.0000000000001596 PMC5482177

[CR39] Davey, J., Turner, R. M., Clarke, M. J., & Higgins, J. P. (2011). Characteristics of meta-analyses and their component studies in the Cochrane Database of Systematic Reviews: A cross-sectional, descriptive analysis. *BMC Medical Research Methodology,**11*(1471–2288), 160. 10.1186/1471-2288-11-160 22114982 10.1186/1471-2288-11-160 PMC3247075

[CR40] * Davis, A., Wolf, T. J., & Foster, E. R. (2019). Complex task performance assessment (CTPA) and functional cognition in people with Parkinson's disease. *American Journal of Occupational Therapy*, *73*(5), 7305205060p7305205061–7305205060p7305205069. 10.5014/ajot.2019.031492 10.5014/ajot.2019.031492 PMC6813512 31484030

[CR41] de Silva, A., Neel, M. L., Maitre, N., Busch, T., & Taylor, H. G. (2021). Resilience and vulnerability in very preterm 4-year-olds. *Clinical Neuropsychologist,**35*(5), 904–924. 10.1080/13854046.2020.1817565 32924801 10.1080/13854046.2020.1817565

[CR42] Downes, M., Keenan, L., Duane, Y., Duffy, K., Fortune, G., Geoghegan, R., Conroy, H., & McMahon, C. (2022). Executive function in children with sickle cell anemia on transfusion: NIH toolbox utility in the clinical context. *Clinical Neuropsychologist,**36*(6), 1573–1588. 10.1080/13854046.2020.1847325 33200651 10.1080/13854046.2020.1847325

[CR43] * Dudley-Javoroski, S., Lee, J., & Shields, R. K. (2022). Cognitive function, quality of life, and aging: Relationships in individuals with and without spinal cord injury. *Physiotherapy Theory and Practice,**38*(1), 36–45. 10.1080/09593985.2020.1712755 31914347 10.1080/09593985.2020.1712755 PMC7702216

[CR44] Dunbar, K. E., Raboy, A. L., Kirby, Z. M., Taylor, P. L., & Roy, M. J. (2019). Distinguishing the relative impact of post-traumatic stress disorder and traumatic brain injury on iPad-measured cognitive function. *Cyberpsychology, Behavior, and Social Networking,**22*(12), 761–765. 10.1089/cyber.2019.0296 31841649 10.1089/cyber.2019.0296

[CR45] * Dutta, M. (2020). Evaluating the relationship between executive functioning, spoken discourse, and life participation in aphasia [Doctoral dissertation, Indiana University]. ProQuest.

[CR46] Duval, S., & Tweedie, R. (2000). Trim and fill: A simple funnel-plot-based method of testing and adjusting for publication bias in meta-analysis. *Biometrics,**56*(2), 455–463. 10.1111/j.0006-341x.2000.00455.x 10877304 10.1111/j.0006-341x.2000.00455.x

[CR47] Egger, M., Davey Smith, G., Schneider, M., & Minder, C. (1997). Bias in meta-analysis detected by a simple, graphical test. *BMJ,**315*(7109), 629–634. 10.1136/bmj.315.7109.629 9310563 10.1136/bmj.315.7109.629 PMC2127453

[CR48] Elias, M. N., Munro, C. L., & Liang, Z. (2020). Executive function, dexterity, and discharge disposition in older intensive care unit survivors. *American Journal of Critical Care,**29*(6), 484–488. 10.4037/ajcc2020132 33130868 10.4037/ajcc2020132 PMC10467841

[CR49] Eneva, K. T., Arlt, J. M., Yiu, A., Murray, S. M., & Chen, E. Y. (2017). Assessment of executive functioning in binge-eating disorder independent of weight status. *International Journal of Eating Disorders,**50*(8), 942–951. 10.1002/eat.22738 28644541 10.1002/eat.22738 PMC5672821

[CR50] * Engel, L. L. (2018). Promoting financial management activities after an acquired brain injury: Enhancing assessment evidence (Publication Number 10690596) [Thesis, University of Toronto]. ProQuest.

[CR51] Fox, R. S., Zhang, M., Amagai, S., Bassard, A., Dworak, E. M., Han, Y. C., Kassanits, J., Miller, C. H., Nowinski, C. J., Giella, A. K., Stoeger, J. N., Swantek, K., Hook, J. N., & Gershon, R. C. (2022). Uses of the NIH Toolbox(R) in clinical samples: A scoping review. Neurology: *Clinical Practice,**12*(4), 307–319. 10.1212/CPJ.0000000000200060 10.1212/CPJ.0000000000200060 PMC9647815 36382124

[CR52] Frazer, K. M., Manly, J. J., Downey, G., & Hart, C. L. (2018). Assessing cognitive functioning in individuals with cocaine use disorder. *Journal of Clinical and Experimental Neuropsychology,**40*(6), 619–632. 10.1080/13803395.2017.1403569 29226762 10.1080/13803395.2017.1403569

[CR53] French, M. A., Cohen, M. L., Pohlig, R. T., & Reisman, D. S. (2021). Fluid cognitive abilities are important for learning and retention of a new, explicitly learned walking pattern in individuals after stroke. *Neurorehabilitation and Neural Repair,**35*(5), 419–430. 10.1177/15459683211001025 33754890 10.1177/15459683211001025 PMC8122051

[CR54] Gershon, R., Nowinski, C., Peipert, J. D., Bedjeti, K., Ustsinovich, V., Hook, J., Fox, R., & Weintraub, S. (2020a). Use of the NIH Toolbox for assessment of mild cognitive impairment and Alzheimer’s disease in general population, African-American and Spanish-speaking samples of older adults. *Alzheimer’s & Dementia,**16*(S6), e043372. 10.1002/alz.043372

[CR55] Gershon, R. C., Cella, D., Fox, N. A., Havlik, R. J., Hendrie, H. C., & Wagster, M. V. (2010). Assessment of neurological and behavioural function: The NIH Toolbox. *The Lancet Neurology,**9*(2), 138–139. 10.1016/S1474-4422(09)70335-7 20129161 10.1016/S1474-4422(09)70335-7

[CR56] Gershon, R. C., Fox, R. S., Manly, J. J., Mungas, D. M., Nowinski, C. J., Roney, E. M., & Slotkin, J. (2020b). The NIH toolbox: Overview of development for use with Hispanic populations. *Journal of the International Neuropsychological Society,**26*(6), 567–575. 10.1017/S1355617720000028 32063249 10.1017/S1355617720000028 PMC7319898

[CR57] Gershon, R. C., Slotkin, J., Manly, J. J., Blitz, D. L., Beaumont, J. L., Schnipke, D., Wallner-Allen, K., Golinkoff, R. M., Gleason, J. B., Hirsh-Pasek, K., Adams, M. J., & Weintraub, S. (2013). NIH TOOLBOX COGNITION BATTERY (CB): Measuring language (vocabulary comprehension and reading decoding). *Monographs of the Society for Research in Child Development*, *78*(4), 49–69. http://www.jstor.org.ezproxy.lib.utah.edu/stable/43772790 10.1111/mono.12034 PMC7659464 23952202

[CR58] Gills, J. L., Bott, N. T., Madero, E. N., Glenn, J. M., & Gray, M. (2021). A short digital eye-tracking assessment predicts cognitive status among adults. *Geroscience,**43*(1), 297–308. 10.1007/s11357-020-00254-5 32870437 10.1007/s11357-020-00254-5 PMC8050116

[CR59] Gladstone, E., Narad, M. E., Hussain, F., Quatman-Yates, C. C., Hugentobler, J., Wade, S. L., Gubanich, P. J., & Kurowski, B. G. (2019). Neurocognitive and quality of life improvements associated with aerobic training for individuals with persistent symptoms after mild traumatic brain injury: Secondary outcome analysis of a pilot randomized clinical trial. *Frontiers in Neurology,**10*, 1002. 10.3389/fneur.2019.01002 31620073 10.3389/fneur.2019.01002 PMC6759771

[CR60] Hackett, K., Krikorian, R., Giovannetti, T., Melendez-Cabrero, J., Rahman, A., Caesar, E. E., Chen, J. L., Hristov, H., Seifan, A., Mosconi, L., & Isaacson, R. S. (2018). Utility of the NIH Toolbox for assessment of prodromal Alzheimer’s disease and dementia. *Alzheimer’s & Dementia,**10*, 764–772. 10.1016/j.dadm.2018.10.002 10.1016/j.dadm.2018.10.002 PMC6247399 30505926

[CR61] Hartman, S. J., Nelson, S. H., Myers, E., Natarajan, L., Sears, D. D., Palmer, B. W., Weiner, L. S., Parker, B. A., & Patterson, R. E. (2018). Randomized controlled trial of increasing physical activity on objectively measured and self-reported cognitive functioning among breast cancer survivors: The memory & motion study. *Cancer,**124*(1), 192–202. 10.1002/cncr.30987 28926676 10.1002/cncr.30987 PMC5735009

[CR62] Hawkins, M. A. W., Colaizzi, J. M., Cole, A. B., Keirns, N. G., Smith, C. E., Stout, M., Chaney, J., Sawhney, M., & Gahn, D. (2021a). Pilot trial of acceptance-based behavioral weight loss and neurocognition among American Indians. *Behavior Therapy,**52*(2), 350–364. 10.1016/j.beth.2020.04.009 33622505 10.1016/j.beth.2020.04.009 PMC8694275

[CR63] Hawkins, M. A. W., Keirns, N. G., Baraldi, A. N., Layman, H. M., Stout, M. E., Smith, C. E., Gunstad, J., Hildebrand, D. A., Vohs, K. D., & Lovallo, W. R. (2021b). Baseline associations between biomarkers, cognitive function, and self-regulation indices in the Cognitive and Self-regulatory Mechanisms of Obesity Study. *Obesity Science & Practice,**7*(6), 669–681. 10.1002/osp4.537 34877006 10.1002/osp4.537 PMC8633928

[CR64] Heaton, R. K., Akshoomoff, N., Tulsky, D., Mungas, D., Weintraub, S., Dikmen, S., Beaumont, J., Casaletto, K. B., Conway, K., Slotkin, J., & Gershon, R. (2014). Reliability and validity of composite scores from the NIH Toolbox Cognition Battery in adults. *Journal of the International Neuropsychological Society,**20*(6), 588–598. 10.1017/S1355617714000241 24960398 10.1017/S1355617714000241 PMC4103963

[CR65] Heaton, R. K., Miller, S. W., Taylor, J. T., & Grant, I. (2004). Revised comprehensive norms for an expanded Halstead-Reitan battery: Demographically adjusted neuropsychological norms for African American and Caucasian adults, professional manual. In: Psychological Assessment Resources.

[CR66] * Henry, B. (2020). The role of opioid dose, duration of opioid use, and pain intensity in cognitive functioning and quality of life in chronic pain patients (Publication Number 28026895) [Doctoral dissertation, Alliant International University]. ProQuest. San Diego, CA.

[CR67] Higgins, J., & Green, S. (Eds.). 2011. Cochrane handbook for systematic reviews of interventions (Version 5.1.0). *The Cochrane Collaboration.*

[CR68] Higgins, J., Thompson, S. G., Deeks, J. J., & Altman, D. G. (2003). Measuring inconsistency in meta-analyses. *British Medical Journal,**327*(7414), 557–560. 10.1136/bmj.327.7414.557 12958120 10.1136/bmj.327.7414.557 PMC192859

[CR69] Hoffman, R. M., Trevarrow, M. P., Bergwell, H. R., Embury, C. M., Heinrichs-Graham, E., Wilson, T. W., & Kurz, M. J. (2021). Cortical oscillations that underlie working memory are altered in adults with cerebral palsy. *Clinical Neurophysiology,**132*(4), 938–945. 10.1016/j.clinph.2020.12.029 33636609 10.1016/j.clinph.2020.12.029 PMC8218310

[CR70] * Hollister, J. E. (2015). Effortful control and adaptive functioning in school-age children who stutter (Publication Number 37266894) [Thesis, University of Iowa]. Proquest.

[CR71] Hook, J., & Giella, A. K. (2023). National Institutes of Health (NIH) Toolbox V3 Administration Manual In: Toolbox Assessments, Inc.

[CR72] Hoover, D. W., & Romero, E. M. G. (2019). The interactive trauma scale: A web-based measure for children with autism. *Journal of Autism and Developmental Disorders,**49*(4), 1686–1692. 10.1007/s10803-018-03864-3 30604349 10.1007/s10803-018-03864-3

[CR73] Horn, J. L. (1968). Organization of abilities and the development of intelligence. *Psychological Review,**75*(3), 242–259. 10.1037/h0025662 4875815 10.1037/h0025662

[CR74] * Hwang, G., Dabbs, K., Conant, L., Nair, V. A., Mathis, J., Almane, D. N., Nencka, A., Birn, R., Humphries, C., Raghavan, M., DeYoe, E. A., Struck, A. F., Maganti, R., Binder, J. R., Meyerand, E., Prabhakaran, V., & Hermann, B. (2019). Cognitive slowing and its underlying neurobiology in temporal lobe epilepsy. *Cortex*, *117*, 41–52. 10.1016/j.cortex.2019.02.022 10.1016/j.cortex.2019.02.022 PMC6650302 30927560

[CR75] Jones, D. R., Macias, C., Barreira, P. J., Fisher, W. H., Hargreaves, W. A., & Harding, C. M. (2004). Prevalence, severity, and co-occurrence of chronic physical health problems of persons with serious mental illness. *Psychiatric Services,**55*(11), 1250–1257. 15534013 10.1176/appi.ps.55.11.1250 PMC2759895

[CR76] Jim, H. S., Phillips, K. M., Chait, S., Faul, L. A., Popa, M. A., Lee, Y. H., Hussin, M. G., Jacobsen, P. B., & Small, B. J. (2012). Meta-analysis of cognitive functioning in breast cancer survivors previously treated with standard-dose chemotherapy. *Journal of Clinical Oncology,**30*(29), 3578–3587. 10.1200/JCO.2011.39.5640 22927526 10.1200/JCO.2011.39.5640 PMC3462044

[CR77] Kaat, A. J., McKenzie, F. J., Shields, R. H., LaForte, E., Coleman, J., Michalak, C., & Hessl, D. R. (2022). Assessing processing speed among individuals with intellectual and developmental disabilities: A match-to-sample paradigm. *Child Neuropsychology,**28*(1), 1–13. 10.1080/09297049.2021.1938987 34126855 10.1080/09297049.2021.1938987 PMC8648883

[CR78] Kalyani, H. H. N., Sullivan, K. A., Moyle, G., Brauer, S., Jeffrey, E. R., & Kerr, G. K. (2019). Impacts of dance on cognition, psychological symptoms and quality of life in Parkinson’s disease. *NeuroRehabilitation,**45*(2), 273–283. 10.3233/NRE-192788 31561398 10.3233/NRE-192788

[CR79] Kamerer, A. M., AuBuchon, A., Fultz, S. E., Kopun, J. G., Neely, S. T., & Rasetshwane, D. M. (2019). The role of cognition in common measures of peripheral synaptopathy and hidden hearing loss. *American Journal of Audiology,**28*(4), 843–856. 10.1044/2019_AJA-19-0063 31647880 10.1044/2019_AJA-19-0063 PMC7210438

[CR80] Kang, H. (2013). The prevention and handling of the missing data. *Korean Journal of Anesthesiology,**64*(5), 402–406. 10.4097/kjae.2013.64.5.402 23741561 10.4097/kjae.2013.64.5.402 PMC3668100

[CR81] Karawani, H., Jenkins, K., & Anderson, S. (2018). Restoration of sensory input may improve cognitive and neural function. *Neuropsychologia,**114*, 203–213. 10.1016/j.neuropsychologia.2018.04.041 29729278 10.1016/j.neuropsychologia.2018.04.041 PMC5988995

[CR82] Karsdorp, P. A., Everaerd, W., Kindt, M., & Mulder, B. J. (2007). Psychological and cognitive functioning in children and adolescents with congenital heart disease: A meta-analysis. *Journal of Pediatric Psychology,**32*(5), 527–541. 10.1093/jpepsy/jsl047 17182669 10.1093/jpepsy/jsl047

[CR83] Keyes, K. M., Platt, J., Kaufman, A. S., & McLaughlin, K. A. (2017). Association of fluid intelligence and psychiatric disorders in a population-representative sample of US adolescents. *JAMA Psychiatry,**74*(2), 179–188. 10.1001/jamapsychiatry.2016.3723 28030746 10.1001/jamapsychiatry.2016.3723 PMC5288266

[CR84] * Kim, M., Liotta, E. M., Maas, M. B., Braun, R. I., Garcia-Canga, B., Ganger, D. R., Ladner, D. P., Reid, K. J., & Zee, P. C. (2021). Rest-activity rhythm disturbance in liver cirrhosis and association with cognitive impairment. *Sleep*, *44*(6). 10.1093/sleep/zsaa288 10.1093/sleep/zsaa288 PMC8193561 33367862

[CR85] King, D. L. O., Henson, R. N., Kievit, R., Wolpe, N., Brayne, C., Tyler, L. K., Rowe, J. B., Bullmore, E. T., Calder, A. C., Cusack, R., Dalgleish, T., Duncan, J., Matthews, F. E., Marslen-Wilson, W. D., Shafto, M. A., Campbell, K., Cheung, T., Davis, S., Geerligs, L., & Cam, C. A. N. (2023). Distinct components of cardiovascular health are linked with age-related differences in cognitive abilities. *Scientific Reports,**13*(1), 978. 10.1038/s41598-022-27252-1 36653428 10.1038/s41598-022-27252-1 PMC9849401

[CR86] Koopowitz, S. M., Mare, K. T., Zar, H. J., Stein, D. J., & Ipser, J. C. (2021). The neurocognitive profile of post-traumatic stress disorder (PTSD), major depressive disorder (MDD), and PTSD with comorbid MDD. *Brain and Behavior,**11*(4), Article e01950. 10.1002/brb3.1950 33666359 10.1002/brb3.1950 PMC8035469

[CR87] Kratz, A. L., Whibley, D., Kim, S., Sliwinski, M., Clauw, D., & Williams, D. A. (2020). Fibrofog in daily life: An examination of ambulatory subjective and objective cognitive function in fibromyalgia. *Arthritis Care & Research,**72*(12), 1669–1677. 10.1002/acr.24089 31609548 10.1002/acr.24089 PMC7153985

[CR88] * Kringle, E.A. (2019). Influencing sedentary behavior through participation after stroke. (Publication No. 13858094). [Doctoral dissertation, University of Pittsburgh]. ProQuest.

[CR89] Kringle, E. A., Novelli, E. M., Butters, M. A., & Skidmore, E. R. (2023). Validation of the NIH Toolbox-Cognition Battery against legacy neurocognitive measures in adults with cognitive impairments: An exploratory analysis. *Journal of the International Neuropsychological Society,**29*(5), 472–479. 10.1017/S1355617722000406 36062530 10.1017/S1355617722000406 PMC9985667

[CR90] Kunker, K., Peters, D. M., & Mohapatra, S. (2020). Long-term impact of mild traumatic brain injury on postural stability and executive function. *Neurological Sciences,**41*(7), 1899–1907. 10.1007/s10072-020-04300-0 32095948 10.1007/s10072-020-04300-0

[CR91] Liberati, A., Altman, D. G., Tetzlaff, J., Mulrow, C., Gotzsche, P. C., Ioannidis, J. P., Clarke, M., Devereaux, P. J., Kleijnen, J., & Moher, D. (2009). The PRISMA statement for reporting systematic reviews and meta-analyses of studies that evaluate health care interventions: Explanation and elaboration. *PLoS Medicine,**6*(7), Article e1000100. 10.1371/journal.pmed.1000100 19621070 10.1371/journal.pmed.1000100 PMC2707010

[CR92] Lovden, M., Fratiglioni, L., Glymour, M. M., Lindenberger, U., & Tucker-Drob, E. M. (2020). Education and cognitive functioning across the life span. *Psychological Science in the Public Interest,**21*(1), 6–41. 10.1177/1529100620920576 32772803 10.1177/1529100620920576 PMC7425377

[CR93] Ludyga, S., Puhse, U., Gerber, M., & Mucke, M. (2021). Muscle strength and executive function in children and adolescents with autism spectrum disorder. *Autism Research,**14*(12), 2555–2563. 10.1002/aur.2587 34351051 10.1002/aur.2587 PMC9292567

[CR94] Lundine, J. P., Harnish, S. M., McCauley, R. J., Zezinka, A. B., Blackett, D. S., & Fox, R. A. (2018). Exploring summarization differences for two types of expository discourse in adolescents with traumatic brain injury. *American Journal of Speech-Language Pathology,**27*(1), 247–257. 10.1044/2017_AJSLP-16-0131 29121200 10.1044/2017_AJSLP-16-0131

[CR95] Maas, M. B., Lizza, B. D., Kim, M., Gendy, M., Liotta, E. M., Reid, K. J., Zee, P. C., & Griffith, J. W. (2021). The feasibility and validity of objective and patient-reported measurements of cognition during early critical illness recovery. *Neurocritical Care,**34*(2), 403–412. 10.1007/s12028-020-01126-8 33094468 10.1007/s12028-020-01126-8 PMC8060361

[CR96] *MacIsaac, J. (2018). Group separation and classification of non-suicidal self-injury in a university student population. (Publication No. 10973632) [Master’s thesis, University of Windsor]. ProQuest.

[CR97] Manglani, H. R., Fisher, M. E., Duraney, E. J., Nicholas, J. A., & Prakash, R. S. (2022). A promising cognitive screener in multiple sclerosis: The NIH toolbox cognition battery concords with gold standard neuropsychological measures. *Multiple Sclerosis,**28*(11), 1762–1772. 10.1177/13524585221088731 35531593 10.1177/13524585221088731 PMC10315105

[CR98] Marinac, C. R., Nelson, S. H., Cadmus-Bertram, L., Kerr, J., Natarajan, L., Godbole, S., & Hartman, S. J. (2019). Dimensions of sedentary behavior and objective cognitive functioning in breast cancer survivors. *Supportive Care in Cancer,**27*(4), 1435–1441. 10.1007/s00520-018-4459-8 30225570 10.1007/s00520-018-4459-8 PMC6391205

[CR99] Mazzoli, E., Salmon, J., Pesce, C., Teo, W. P., Rinehart, N., May, T., & Barnett, L. M. (2021). Effects of classroom-based active breaks on cognition, sitting and on-task behaviour in children with intellectual disability: A pilot study. *Journal of Intellectual Disability Research,**65*(5), 464–488. 10.1111/jir.12826 33719112 10.1111/jir.12826

[CR100] McDonough, I. M., Bischof, G. N., Kennedy, K. M., Rodrigue, K. M., Farrell, M. E., & Park, D. C. (2016). Discrepancies between fluid and crystallized ability in healthy adults: a behavioral marker of preclinical Alzheimer’s disease. *Neurobiology of Aging,**46*, 68–75. 10.1016/j.neurobiolaging.2016.06.011 27460151 10.1016/j.neurobiolaging.2016.06.011 PMC5018443

[CR101] McHugh, M. L. (2012). Interrater reliability: The kappa statistic. *Biochemia Medica,**22*(3), 276–282. 23092060 PMC3900052

[CR102] McTeague, L. M., Goodkind, M. S., & Etkin, A. (2016). Transdiagnostic impairment of cognitive control in mental illness. *Journal of Psychiatric Research,**83*, 37–46. 10.1016/j.jpsychires.2016.08.001 27552532 10.1016/j.jpsychires.2016.08.001 PMC5107153

[CR103] McTeague, L. M., Huemer, J., Carreon, D. M., Jiang, Y., Eickhoff, S. B., & Etkin, A. (2017). Identification of common neural circuit disruptions in cognitive control across psychiatric disorders. *American Journal of Psychiatry,**174*(7), 676–685. 10.1176/appi.ajp.2017.16040400 28320224 10.1176/appi.ajp.2017.16040400 PMC5543416

[CR104] Meier, E. L., Kelly, C. R., Goldberg, E. B., & Hillis, A. E. (2022). Executive control deficits and lesion correlates in acute left hemisphere stroke survivors with and without aphasia. *Brain Imaging and Behavior,**16*(2), 868–877. 10.1007/s11682-021-00580-y 34647269 10.1007/s11682-021-00580-y PMC8514281

[CR105] Meredith, L. R., Lim, A. C., & Ray, L. A. (2020). Neurocognitive performance in alcohol use disorder using the NIH toolbox: Role of severity and sex differences. *Drug and Alcohol Dependence,**216*, Article 108269. 10.1016/j.drugalcdep.2020.108269 32906037 10.1016/j.drugalcdep.2020.108269 PMC7972314

[CR106] Modi, A. C., Vannest, J., Combs, A., Turnier, L., & Wade, S. L. (2018). Pattern of executive functioning in adolescents with epilepsy: A multimethod measurement approach. *Epilepsy & Behavior,**80*, 5–10. 10.1016/j.yebeh.2017.12.021 29396361 10.1016/j.yebeh.2017.12.021

[CR107] Moher, D., Shamseer, L., Clarke, M., Ghersi, D., Liberati, A., Petticrew, M., Shekelle, P., Stewart, L. A., & Group, P.-P. (2015). Preferred reporting items for systematic review and meta-analysis protocols (PRISMA-P) 2015 statement. *Systematic Reviews,**4*(1), 1. 10.1186/2046-4053-4-1 25554246 10.1186/2046-4053-4-1 PMC4320440

[CR108] Molinaro, M., Adams, H. R., Mwanza-Kabaghe, S., Mbewe, E. G., Kabundula, P. P., Mweemba, M., Birbeck, G. L., & Bearden, D. R. (2021). Evaluating the relationship between depression and cognitive function among children and adolescents with HIV in Zambia. *AIDS and Behavior,**25*(9), 2669–2679. 10.1007/s10461-021-03193-0 33630200 10.1007/s10461-021-03193-0 PMC8456506

[CR109] Moore, B. F., Shapiro, A. L., Wilkening, G., Magzamen, S., Starling, A. P., Allshouse, W. B., Adgate, J. L., & Dabelea, D. (2020). Prenatal exposure to tobacco and offspring neurocognitive development in the healthy start study. *Journal of Pediatrics,**218*(28–34), Article e22. 10.1016/j.jpeds.2019.10.056 10.1016/j.jpeds.2019.10.056 PMC7042047 31759580

[CR110] Moran, T. P. (2016). Anxiety and working memory capacity: A meta-analysis and narrative review. *Psychological Bulletin,**142*(8), 831–864. 10.1037/bul0000051 26963369 10.1037/bul0000051

[CR111] * Moriarty, T. A. (2019). Exercise-based cardiac rehabilitation improves cognitive function among CVD patients (Publication Number 22584154) [Doctoral dissertation, University of New Mexico]. ProQuest. Albuquerque, NM.

[CR112] Morrow, E. L., Dulas, M. R., Cohen, N. J., & Duff, M. C. (2020). Relational memory at short and long delays in individuals with moderate-severe traumatic brain injury. *Frontiers in Human Neuroscience,**14*, 270. 10.3389/fnhum.2020.00270 32754022 10.3389/fnhum.2020.00270 PMC7366514

[CR113] Mulhauser, K., Reynolds, E. L., Callaghan, B. C., Fierro, C., Giordani, B., & Votruba, K. (2021). Executive functioning in extreme obesity: Contributions from metabolic status, medical comorbidities, and psychiatric factors. *Obesity Surgery,**31*(6), 2669–2681. 10.1007/s11695-021-05319-8 33660154 10.1007/s11695-021-05319-8 PMC11632671

[CR114] Mungas, D., Heaton, R., Tulsky, D., Zelazo, P. D., Slotkin, J., Blitz, D., Lai, J. S., & Gershon, R. (2014). Factor structure, convergent validity, and discriminant validity of the NIH Toolbox cognitive health battery (NIHTB-CHB) in adults. *Journal of the International Neuropsychological Society,**20*(6), 579–587. 10.1017/S1355617714000307 24960474 10.1017/S1355617714000307 PMC4103956

[CR115] Naguib, J. M., Kulinskaya, E., Lomax, C. L., & Garralda, M. E. (2009). Neuro-cognitive performance in children with type 1 diabetes—A meta-analysis. *Journal of Pediatric Psychology,**34*(3), 271–282. 10.1093/jpepsy/jsn074 18635605 10.1093/jpepsy/jsn074

[CR116] Nashiro, K., Yoo, H. J., Cho, C., Min, J., Feng, T., Nasseri, P., Bachman, S. L., Lehrer, P., Thayer, J. F., & Mather, M. (2023). Effects of a randomised trial of 5-week heart rate variability biofeedback intervention on cognitive function: Possible benefits for inhibitory control. *Applied Psychophysiology and Biofeedback,**48*(1), 35–48. 10.1007/s10484-022-09558-y 36030457 10.1007/s10484-022-09558-y PMC9420180

[CR117] National Institutes of Health and Centers (2024). Adolescent brain cognitive development (ABCD) study. (Version 5.1) . National Institute of Mental Health Data Archive. 10.15154/z563-zd24

[CR118] *The NIH Blueprint for Neuroscience Research*. https://neuroscienceblueprint.nih.gov/

[CR119] Norman, R. S., Shah, M. N., & Turkstra, L. S. (2019). Reaction time and cognitive-linguistic performance in adults with mild traumatic brain injury. *Brain Injury,**33*(9), 1173–1183. 10.1080/02699052.2019.1632487 31291747 10.1080/02699052.2019.1632487 PMC6696949

[CR120] Novak, A., Vizjak, K., & Rakusa, M. (2022). Cognitive impairment in people with epilepsy. *Journal of Clinical Medicine,**11*(1), 267. 10.3390/jcm11010267 35012007 10.3390/jcm11010267 PMC8746065

[CR121] Ott, L. R., Schantell, M., Willett, M. P., Johnson, H. J., Eastman, J. A., Okelberry, H. J., Wilson, T. W., Taylor, B. K., & May, P. E. (2022). Construct validity of the NIH toolbox cognitive domains: A comparison with conventional neuropsychological assessments. *Neuropsychology,**36*(5), 468–481. 10.1037/neu0000813 35482626 10.1037/neu0000813 PMC10468104

[CR122] * Paolillo, E. W. (2021). Using ecological momentary assessment to improve assessment of self-reported cognitive difficulties among adults with comorbid hiv and heavy alcohol use [Doctoral dissertation, University of California, San Diego; San Diego State University].

[CR123] Papalambros, N. A., Weintraub, S., Chen, T., Grimaldi, D., Santostasi, G., Paller, K. A., Zee, P. C., & Malkani, R. G. (2019). Acoustic enhancement of sleep slow oscillations in mild cognitive impairment. *Annals of Clinical and Translational Neurology,**6*(7), 1191–1201. 10.1002/acn3.796 31353857 10.1002/acn3.796 PMC6649400

[CR124] * Pardej, S.K.(2020). A psychometric evaluation of computerized attention measures in young children with neurofibromatosis type 1. [Master’s thesis, University of Wisconsin Milwaukee]. UWM Digital Commons. https://dc.uwm.edu/etd/2574

[CR125] Pendergrass, J. C., Targum, S. D., & Harrison, J. E. (2018). Cognitive impairment associated with cancer: A brief review. *Innovations in Clinical Neuroscience,**15*(1–2), 36–44. 29497579 PMC5819720

[CR126] Pozar, R., Giordani, B., & Kavcic, V. (2020). Effective differentiation of mild cognitive impairment by functional brain graph analysis and computerized testing. *PLoS ONE,**15*(3), Article e0230099. 10.1371/journal.pone.0230099 32176709 10.1371/journal.pone.0230099 PMC7075594

[CR127] Prussien, K. V., Compas, B. E., Siciliano, R. E., Ciriegio, A. E., Lee, C. A., Kassim, A. A., DeBaun, M. R., Donahue, M. J., & Jordan, L. C. (2021). Cerebral hemodynamics and executive function in sickle cell anemia. *Stroke,**52*(5), 1830–1834. 10.1161/STROKEAHA.120.032741 33840223 10.1161/STROKEAHA.120.032741 PMC8483619

[CR128] Prussien, K. V., Jordan, L. C., DeBaun, M. R., & Compas, B. E. (2019). Cognitive function in sickle cell disease across domains, cerebral infarct status, and the lifespan: A meta-analysis. *Journal of Pediatric Psychology,**44*(8), 948–958. 10.1093/jpepsy/jsz031 31050352 10.1093/jpepsy/jsz031 PMC6706005

[CR129] Read, N., Mulraney, M., McGillivray, J., & Sciberras, E. (2020). Comorbid anxiety and irritability symptoms and their association with cognitive functioning in children with ADHD. *Journal of Abnormal Child Psychology,**48*(8), 1035–1046. 10.1007/s10802-020-00658-z 32462307 10.1007/s10802-020-00658-z

[CR130] Rebchuk, A. D., Kuzmuk, L. E., Deptuck, H. M., Silverberg, N. D., & Field, T. S. (2022). Evaluating high-functioning young stroke survivors with cognitive complaints. *Canadian Journal of Neurological Sciences,**49*(3), 368–372. 10.1017/cjn.2021.137 10.1017/cjn.2021.137 34134793

[CR131] Richerson, W. T., Umfleet, L. G., Schmit, B. D., & Wolfgram, D. F. (2021). Changes in cerebral volume and white matter integrity in adults on hemodialysis and relationship to cognitive function. *Nephron,**145*(1), 35–43. 10.1159/000510614 33049742 10.1159/000510614 PMC7785532

[CR132] Robinson, K. E., Kuttesch, J. F., Champion, J. E., Andreotti, C. F., Hipp, D. W., Bettis, A., Barnwell, A., & Compas, B. E. (2010). A quantitative meta-analysis of neurocognitive sequelae in survivors of pediatric brain tumors. *Pediatric Blood & Cancer,**55*(3), 525–531. 10.1002/pbc.22568 20658625 10.1002/pbc.22568

[CR133] Rockhold, M. N., Krueger, A. M., de Water, E., Lindgren, C. W., Sandness, K. E., Eckerle, J. K., Schumacher, M. J., Fink, B. A., Boys, C. J., Carlson, S. M., Fuglestad, A. J., Mattson, S. N., Jones, K. L., Riley, E. P., & Wozniak, J. R. (2021). Executive and social functioning across development in children and adolescents with prenatal alcohol exposure. *Alcoholism, Clinical and Experimental Research,**45*(2), 457–469. 10.1111/acer.14538 33349933 10.1111/acer.14538 PMC7887051

[CR134] Russell-Schulz, B., Vavasour, I. M., Zhang, J., MacKay, A. L., Purcell, V., Muller, A. M., Brucar, L. R., Torres, I. J., Panenka, W. J., & Virji-Babul, N. (2021). Myelin water fraction decrease in individuals with chronic mild traumatic brain injury and persistent symptoms. *Heliyon,**7*(4), Article e06709. 10.1016/j.heliyon.2021.e06709 33898831 10.1016/j.heliyon.2021.e06709 PMC8056430

[CR135] Sanborn, V., Gunstad, J., Shrestha, R., Mistler, C. B., & Copenhaver, M. M. (2022). Cognitive profiles in persons with opioid use disorder enrolled in methadone treatment. *Applied Neuropsychology: Adult,**29*(4), 462–468. 10.1080/23279095.2020.1769099 32463730 10.1080/23279095.2020.1769099 PMC7704535

[CR136] Schillerstrom, J. E., Horton, M. S., & Royall, D. R. (2005). The impact of medical illness on executive function. *Psychosomatics,**46*(6), 508–516. 10.1176/appi.psy.46.6.508 16288129 10.1176/appi.psy.46.6.508

[CR137] Schmithorst, V. J., Badaly, D., Beers, S. R., Lee, V. K., Weinberg, J., Lo, C. W., & Panigrahy, A. (2022). Relationships between regional cerebral blood flow and neurocognitive outcomes in children and adolescents with congenital heart disease. *Seminars in Thoracic and Cardiovascular Surgery,**34*(4), 1285–1295. 10.1053/j.semtcvs.2021.10.014 34767938 10.1053/j.semtcvs.2021.10.014 PMC9085965

[CR138] * Scholl, J. L., Espinoza, A. I., Rai, W., Leedom, M., Baugh, L. A., Berg-Poppe, P., & Singh, A. (2021). Relationships between freezing of gait severity and cognitive deficits in Parkinson's disease. *Brain Sciences*, *11*(11). 10.3390/brainsci11111496 10.3390/brainsci11111496 PMC8615553 34827496

[CR139] Scott, J. C., Matt, G. E., Wrocklage, K. M., Crnich, C., Jordan, J., Southwick, S. M., Krystal, J. H., & Schweinsburg, B. C. (2015). A quantitative meta-analysis of neurocognitive functioning in posttraumatic stress disorder. *Psychological Bulletin,**141*(1), 105–140. 10.1037/a0038039 25365762 10.1037/a0038039 PMC4293317

[CR140] Sedgwick, P., & Marston, L. (2015). How to read a funnel plot in a meta-analysis. *BMJ,**351*, Article h4718. 10.1136/bmj.h4718 26377337 10.1136/bmj.h4718

[CR141] Semkovska, M., Quinlivan, L., O’Grady, T., Johnson, R., Collins, A., O’Connor, J., Knittle, H., Ahern, E., & Gload, T. (2019). Cognitive function following a major depressive episode: A systematic review and meta-analysis. *Lancet Psychiatry,**6*(10), 851–861. 10.1016/S2215-0366(19)30291-3 31422920 10.1016/S2215-0366(19)30291-3

[CR142] Shapiro, A. L. B., Dabelea, D., Stafford, J. M., D’Agostino, R., Pihoker, C., Liese, A. D., Shah, A. S., Bellatorre, A., Lawrence, J. M., Henkin, L., Saydah, S., Wilkening, G., & Group, S. f. D. i. Y. S. (2021). Cognitive function in adolescents and young adults with youth-onset type 1 versus type 2 diabetes: The SEARCH for diabetes in youth study. *Diabetes Care,**44*(6), 1273–1280. 10.2337/dc20-2308 33905344 10.2337/dc20-2308 PMC8247514

[CR143] Sharma, V., Coleman, S., Nixon, J., Sharples, L., Hamilton-Shield, J., Rutter, H., & Bryant, M. (2019). A systematic review and meta-analysis estimating the population prevalence of comorbidities in children and adolescents aged 5 to 18 years. *Obesity Reviews,**20*(10), 1341–1349. 31342672 10.1111/obr.12904 PMC6851579

[CR144] Shiau, S., Arpadi, S. M., Shen, Y., Cantos, A., Ramon, C. V., Shah, J., Jang, G., Manly, J. J., Brickman, A. M., Baccarelli, A. A., & Yin, M. T. (2021). epigenetic aging biomarkers associated with cognitive impairment in older african american adults with human immunodeficiency virus (HIV). *Clinical Infectious Diseases,**73*(11), 1982–1991. 10.1093/cid/ciab563 34143869 10.1093/cid/ciab563 PMC8664485

[CR145] Siciliano, R. E., Murphy, L. K., Prussien, K. V., Henry, L. M., Watson, K. H., Patel, N. J., Lee, C. A., McNally, C. M., Markham, L. W., Compas, B. E., & Jordan, L. C. (2021). Cognitive and attentional function in children with hypoplastic left heart syndrome: A pilot study. *Journal of Clinical Psychology in Medical Settings,**28*(3), 619–626. 10.1007/s10880-020-09753-1 33222094 10.1007/s10880-020-09753-1 PMC8140062

[CR146] Siciliano, R. E., Thigpen, J. C., Desjardins, L., Cook, J. L., Steele, E. H., Gruhn, M. A., Ichinose, M., Park, S., Esbenshade, A. J., Pastakia, D., Wellons, J. C., & Compas, B. E. (2022). Working memory training in pediatric brain tumor survivors after recent diagnosis: Challenges and initial effects. *Applied Neuropsychology. Child,**11*(3), 412–421. 10.1080/21622965.2021.1875226 33501845 10.1080/21622965.2021.1875226 PMC11913254

[CR147] Sinha, P., Wong, A. W. K., Kallogjeri, D., & Piccirillo, J. F. (2018). Baseline cognition assessment among patients with oropharyngeal cancer using PROMIS and NIH toolbox. *JAMA Otolaryngology-Head & Neck Surgery,**144*(11), 978–987. 10.1001/jamaoto.2018.0283 29710116 10.1001/jamaoto.2018.0283 PMC6248179

[CR148] * Slack, J. A. (2018). Relationships between domain-specific cognitive function, functional performance and life satisfaction in persons with chronic obstructive pulmonary disease (COPD) (Publication Number 10902977) [Doctoral dissertation, University of Michigan]. ProQuest.

[CR149] Solomon, M., Gordon, A., Iosif, A. M., Geddert, R., Krug, M. K., Mundy, P., & Hessl, D. (2021). Using the NIH toolbox to assess cognition in adolescents and young adults with autism spectrum disorders. *Autism Research,**14*(3), 500–511. 10.1002/aur.2399 33006263 10.1002/aur.2399 PMC8106946

[CR150] Terry, D. P., Brassil, M., Iverson, G. L., Panenka, W. J., & Silverberg, N. D. (2019). Effect of depression on cognition after mild traumatic brain injury in adults. *Clinical Neuropsychologist,**33*(1), 124–136. 10.1080/13854046.2018.1459853 29726314 10.1080/13854046.2018.1459853

[CR151] Thomas, B. P., Tarumi, T., Wang, C., Zhu, D. C., Tomoto, T., Munro Cullum, C., Dieppa, M., Diaz-Arrastia, R., Bell, K., Madden, C., Zhang, R., & Ding, K. (2021). Hippocampal and rostral anterior cingulate blood flow is associated with affective symptoms in chronic traumatic brain injury. *Brain Research,**1771*, Article 147631. 10.1016/j.brainres.2021.147631 34464600 10.1016/j.brainres.2021.147631 PMC8490292

[CR152] Thompson, M. D., Martin, R. C., Grayson, L. P., Ampah, S. B., Cutter, G., Szaflarski, J. P., & Bebin, E. M. (2020). Cognitive function and adaptive skills after a one-year trial of cannabidiol (CBD) in a pediatric sample with treatment-resistant epilepsy. *Epilepsy & Behavior,**111*, Article 107299. 10.1016/j.yebeh.2020.107299 32759071 10.1016/j.yebeh.2020.107299

[CR153] Tulsky, D. S., Carlozzi, N. E., Chevalier, N., Espy, K. A., Beaumont, J. L., & Mungas, D. (2013). NIH toolbox cognition battery (CB): Measuring working memory. *Monographs of the Society for Research in Child Development*, *78*(4), 70–87. http://www.jstor.org.ezproxy.lib.utah.edu/stable/43772791 10.1111/mono.12035 PMC4427028 23952203

[CR154] Tulsky, D. S., Carlozzi, N. E., Holdnack, J., Heaton, R. K., Wong, A., Goldsmith, A., & Heinemann, A. W. (2017). Using the NIH Toolbox Cognition Battery (NIHTB-CB) in individuals with traumatic brain injury. *Rehabilitation Psychology,**62*(4), 413–424. 10.1037/rep0000174 29265862 10.1037/rep0000174 PMC6462276

[CR155] Van Essen, D. C., Ugurbil, K., Auerbach, E., Barch, D., Behrens, T. E., Bucholz, R., Chang, A., Chen, L., Corbetta, M., Curtiss, S. W., Della Penna, S., Feinberg, D., Glasser, M. F., Harel, N., Heath, A. C., Larson-Prior, L., Marcus, D., Michalareas, G., Moeller, S., Oostenveld, R., … & WU-Minn HCP Consortium. (2012). The Human Connectome Project: a data acquisition perspective. *NeuroImage*, *62*(4), 2222–2231. 10.1016/j.neuroimage.2012.02.018 10.1016/j.neuroimage.2012.02.018 PMC3606888 22366334

[CR156] Verbaan, D., Marinus, J., Visser, M., van Rooden, S. M., Stiggelbout, A. M., Middelkoop, H. A., & van Hilten, J. J. (2007). Cognitive impairment in Parkinson’s disease. *Journal of Neurology, Neurosurgery & Psychiatry,**78*(11), 1182–1187. 17442759 10.1136/jnnp.2006.112367 PMC2117586

[CR157] Walker, K. A., & Brown, G. G. (2018). HIV-associated executive dysfunction in the era of modern antiretroviral therapy: A systematic review and meta-analysis. *Journal of Clinical and Experimental Neuropsychology,**40*(4), 357–376. 10.1080/13803395.2017.1349879 28689493 10.1080/13803395.2017.1349879 PMC6164174

[CR158] Wakaizumi, K., Vigotsky, A. D., Jabakhanji, R., Abdallah, M., Barroso, J., Schnitzer, T. J., Apkarian, A. V., & Baliki, M. N. (2021). Psychosocial, functional, and emotional correlates of long-term opioid use in patients with chronic back pain: A cross-sectional case-control study. *Pain and Therapy,**10*(1), 691–709. 10.1007/s40122-021-00257-w 33844170 10.1007/s40122-021-00257-w PMC8119524

[CR159] Watson, W., Pedowitz, A., Nowak, S., Neumayer, C., Kaplan, E., & Shah, S. (2020). Feasibility of national institutes of health toolbox cognition battery in pediatric brain injury rehabilitation settings. *Rehabilitation Psychology,**65*(1), 22–30. 10.1037/rep0000309 31944784 10.1037/rep0000309

[CR160] *Weintraub, S., Bauer, P. J., Zelazo, P. D., Wallner-*Allen, K., Dikmen, S. S., Heaton, R. K., Tulsky, D. S., Slotkin, J., Blitz, D. L., Carlozzi, N. E., Havlik, R. J., Beaumont, J. L., Mungas, D., Manly, J. J., Borosh, B. G., Nowinski, C. J., & Gershon, R. C. (2013a). I. NIH Toolbox Cognition Battery (CB): Introduction and pediatric data. In *Monographs of the Society for Research in Child Development* (Vol. 78, pp. 1–15). 10.1111/mono.12031 PMC3954750 23952199

[CR161] Weintraub, S., Dikmen, S. S., Heaton, R. K., Tulsky, D. S., Zelazo, P. D., Bauer, P. J., Carlozzi, N. E., Slotkin, J., Blitz, D., Wallner-Allen, K., Fox, N. A., Beaumont, J. L., Mungas, D., Nowinski, C. J., Richler, J., Deocampo, J. A., Anderson, J. E., Manly, J. J., Borosh, B., & Gershon, R. C. (2013b). Cognition assessment using the NIH Toolbox. *Neurology,**80*(11 Suppl 3), S54-64. 10.1212/WNL.0b013e3182872ded 23479546 10.1212/WNL.0b013e3182872ded PMC3662346

[CR162] Whiting, P. F., Rutjes, A. W., Westwood, M. E., Mallett, S., Deeks, J. J., Reitsma, J. B., Leeflang, M. M., Sterne, J. A., Bossuyt, P. M., & QUADAS-2 Group. (2011). QUADAS-2: A revised tool for the quality assessment of diagnostic accuracy studies. *Annals of Internal Medicine,**155*(8), 529–536. 10.7326/0003-4819-155-8-201110180-00009 22007046 10.7326/0003-4819-155-8-201110180-00009

[CR163] World Health Organization. (2022). *International Classification of Diseases Eleventh Revision (ICD-11)*. https://icd.who.int/en

[CR164] Zelazo, P. D., Anderson, J. E., Richler, J., Wallner-Allen, K., Beaumont, J. L., & Weintraub, S. (2013). NIH toolbox cognition battery (CB): Measuring executive function and attention. *Monographs of the Society for Research in Child Development*, *78*(4), 16–33. http://www.jstor.org.ezproxy.lib.utah.edu/stable/43772788 10.1111/mono.12032 23952200

[CR165] Zimmerman, M. E., Kim, M. B., Hale, C., Westwood, A. J., Brickman, A. M., & Shechter, A. (2019). Neuropsychological function response to nocturnal blue light blockage in individuals with symptoms of insomnia: A pilot randomized controlled study. *Journal of the International Neuropsychological Society,**25*(7), 668–677. 10.1017/S1355617719000055 30890197 10.1017/S1355617719000055 PMC7045510

[CR166] * Zuniga, K. E., & Moran, N. E. (2018). Low serum carotenoids are associated with self-reported cognitive dysfunction and inflammatory markers in breast cancer survivors. *Nutrients*, *10*(8). 10.3390/nu10081111 10.3390/nu10081111 PMC6116006 30126098

