# Supplementary material for: A Systematic Review and Meta-Analysis of the Use of the National Institutes of Health Toolbox Cognition Battery in Clinical Populations
Source: Neuropsychol Rev. 2025 Jul 7;36(2):225–60. doi: 10.1007/s11065-025-09669-3 (PMC12875090; doi:10.1007/s11065-025-09669-3)

**Supplemental Table 1**. *Clinical groupings for the included studies*

| Clinical Sample | Number of Studies | Clinical Sub-Group |
| --- | --- | --- |
| Acquired Brain Injury | 2 | Acquired Brain Injury |
| Alcohol Use Disorder | 1 | Substance Use Disorder |
| Alzheimer’s Disease | 1 | Neurodegenerative Disorder |
| Anxiety | 1 |  |
| Aphasia | 1 | Acquired Brain Injury |
| Attention-Deficit/Hyperactivity Disorder | 1 | Neurodevelopmental Disorder |
| Autism Spectrum Disorder | 3 | Neurodevelopmental Disorder |
| Binge Eating Disorder | 1 |  |
| Brain Tumor | 2 | Cancer and Tumor |
| Breast Cancer | 6 | Cancer and Tumor |
| Cardiovascular Disease | 1 | Metabolic and Vascular |
| Cerebral Palsy | 1 | Neurodevelopmental Disorder |
| Chronic Pain | 2 | Pain Syndrome |
| Chronic Obstructive Pulmonary Disease | 1 |  |
| Cirrhosis | 1 | Metabolic and Vascular |
| Cocaine Use Disorder | 1 | Substance Use Disorder |
| Congenital Heart Disease | 2 |  |
| Developmental Delays | 1 | Neurodevelopmental Disorder |
| Diabetes | 1 | Metabolic and Vascular |
| Down Syndrome | 1 | Neurodevelopmental Disorder |
| Encephalitis | 1 | Acquired Brain Injury |
| Epilepsy | 3 | Epilepsy |
| Fibromyalgia | 1 | Pain Syndrome |
| Fragile X | 1 | Neurodevelopmental Disorder |
| Intellectual Disability | 2 | Neurodevelopmental Disorder |
| Hearing Loss | 3 | Hearing Loss |
| Human Immunodeficiency Virus | 5 | Human Immunodeficiency Virus |
| Intensive Care Unit | 2 |  |
| Insomnia | 2 |  |
| Overweight/Obese | 3 | Metabolic and Vascular |
| Major Depressive Disorder | 2 |  |
| Mild Cognitive Impairment | 4 | Neurodegenerative Disorder |
| Neurofibromatosis | 1 | Neurodevelopmental Disorder |
| Non-Suicidal Self Injury | 1 |  |
| Opioid Use Disorder | 1 | Substance Use Disorder |
| Oropharyngeal Cancer | 1 | Cancer and Tumor |
| Orthopedic Injury | 1 |  |
| Other Brain Injury | 1 | Acquired Brain Injury |
| Parkinson’s Disease | 3 | Neurodegenerative Disorder |
| Posttraumatic Stress Disorder | 1 |  |
| Prenatal Alcohol Exposure | 1 |  |
| Prenatal Tobacco Exposure | 1 |  |
| Preterm Children | 1 |  |
| Psychosis | 1 |  |
| Renal Disease | 1 |  |
| Sickle Cell Disease | 2 |  |
| Spinal Cord Injury | 2 |  |
| Stroke | 5 | Acquired Brain Injury |
| Stutter | 1 | Neurodevelopmental Disorder |
| Substance Use Disorder | 1 | Substance Use Disorder |
| Traumatic Brain Injury | 12 | Acquired Brain Injury |
| Vascular Brain Injury | 1 | Acquired Brain Injury |

**Supplemental Table 2.** *QUADAS-2 Signaling Questions*

| **Risk of Bias Domain** | **Signaling Question** |
| --- | --- |
| Patient Selection | - Was a consecutive or random sample of patients enrolled? - Did the study avoid inappropriate exclusions? |
| Index Test (NIHTB-CB) | - Was there a standard administration? |
| Reference Standard  (Clinical Diagnosis) | - Is the reference standard likely to correctly classify the target condition? - Did a qualified person administer or interpret the reference standard? |
| Flow and Timing | - Did all patients receive a reference standard? - Did all patients receive the same reference standard? - Were all enrolled patients included in the analysis (i.e., >89%)? |
| **Applicability Concerns Domain** | **Signaling Question** |
| Patient Selection | - Is there concern that the included patients do not match the review question? |
| Reference Standard | - Is there concern that the target condition as defined by the reference standard does not match the review question? |

**Supplemental Table 3.** *Moderator analyses using meta-regression.*

| **Moderator** | ***k*** | **Coefficient** | **SE** | **95% CI** | **Z value** | **Tau** | ***I^2^*** | **Q** | **df** |
| --- | --- | --- | --- | --- | --- | --- | --- | --- | --- |
| **CFC** | | | | | | | | | |
| Age | 22 | .02 | .01 | [.01, .03] | 2.93** | .62 | 91.20 | 227.34** | 20 |
| Sex | 21 | .01 | .01 | [-.01, .02] | .97 | .62 | 92.26 | 245.40** | 19 |
| Education | 7 | .26 | .06 | [.15, .38] | 4.42** | .36 | 65.38 | 14.44* | 5 |
| **FCC** | | | | | | | | | |
| Age | 27 | .01 | .01 | [.00, .02] | 1.98* | .57 | 88.61 | 219.51** | 25 |
| Sex | 27 | .01 | .00 | [.00, .01] | 1.36 | .52 | 87.70 | 203.24** | 25 |
| Education | 8 | .24 | .05 | [.13, .34] | 4.33** | .33 | 63.07 | 16.25* | 6 |
| **CCC** | | | | | | | | | |
| Age | 21 | .01 | .01 | [.00, .02] | 2.21* | .51 | 84.76 | 124.66** | 19 |
| Sex | 21 | .01 | .00 | [.00, .02] | 2.07* | .48 | 84.97 | 126.42** | 19 |
| Education | 8 | .22 | .05 | [.13, .32] | 4.56** | .25 | 51.19 | 12.29 | 6 |
| **FICA** | | | | | | | | | |
| Age | 56 | .00 | .00 | [-.01, .01] | -.42 | .52 | 91.13 | 608.60** | 54 |
| Sex | 57 | .00 | .00 | [-.01, .01] | .25 | .54 | 91.52 | 648.90** | 55 |
| Education | 14 | .06 | .06 | [-.05, .18] | 1.12 | .46 | 85.52 | 82.88** | 12 |
| **DCCS** | | | | | | | | | |
| Age | 54 | .00 | .00 | [.00, .01] | .82 | .61 | 93.06 | 749.75** | 52 |
| Sex | 55 | .00 | .00 | [.00, .01] | .62 | .65 | 93.82 | 857.25** | 53 |
| Education | 14 | .02 | .11 | [-.18, .22] | .19 | .95 | 95.53 | 268.60** | 12 |
| **LSWM** | | | | | | | | | |
| Age | 53 | .01 | .00 | [.00, .01] | 2.46* | .44 | 88.34 | 437.53** | 51 |
| Sex | 54 | .01 | .00 | [.00, .01] | 1.49 | .47 | 89.62 | 500.97** | 52 |
| Education | 15 | .08 | .05 | [-.02, .19] | 1.65 | .36 | 77.52 | 57.82** | 13 |
| **PCPS** | | | | | | | | | |
| Age | 49 | .00 | .00 | [-.01, .01] | .91 | .71 | 94.96 | 933.45** | 47 |
| Sex | 50 | .00 | .00 | [-.01, .01] | .65 | .74 | 95.29 | 1019.01** | 48 |
| Education | 14 | .13 | .06 | [.02, .24] | 2.23* | .43 | 83.47 | 72.61** | 12 |
| **PSM** | | | | | | | | | |
| Age | 45 | .01 | .00 | [.00, .01] | 1.52 | .40 | 84.21 | 272.35** | 43 |
| Sex | 47 | .00 | .00 | [.00, .01] | 1.61 | .40 | 84.82 | 296.50** | 45 |
| Education | 13 | .07 | .06 | [-.05, .18] | 1.13 | .42 | 82.45 | 62.67** | 11 |
| **ORR** | | | | | | | | | |
| Age | 28 | .01 | .00 | [.00, .02] | 2.56* | .40 | 83.95 | 162.04** | 26 |
| Sex | 27 | .00 | .00 | [,00, .01] | .80 | .47 | 88.86 | 224.44** | 25 |
| Education | 9 | .08 | .05 | [-.02, .18] | 1.59 | .28 | 61.81 | 18.33* | 7 |
| **PV** | | | | | | | | | |
| Age | 30 | .01 | .00 | [.00, .02] | 2.16* | .44 | 85.73 | 196.23** | 28 |
| Sex | 29 | .01 | .00 | [.00, .02] | 2.59* | .42 | 85.57 | 203.10** | 28 |
| Education | 10 | .16 | .06 | [.03, .28] | 2.51* | .49 | 81.83 | 44.02** | 8 |

**Supplemental Table 4.** *Categorical moderator analyses using subgroup comparisons*

| **Moderator** | ***k*** | **Point Estimate (SE)** | **Variance** | **95% CI** | **Z value** | ***p*** | **Tau** | ***Q* (df)** | ***p*** |
| --- | --- | --- | --- | --- | --- | --- | --- | --- | --- |
| **CFC** | | | | | | | | | |
| Test Version |  |  |  |  |  |  |  | .00 (1) | 1.00 |
| Desktop (V1) | 6 | -.33 (.29) | .08 | [-.89, .23] | -1.16 | .25 | .65 |  |  |
| iPad (V2) | 13 | -.33 (.18) | .03 | [-.69, .03] | -1.81 | .07 | .65 |  |  |
| Article Type |  |  |  |  |  |  |  | 2.56 (1) | .11 |
| Peer-Reviewed | 21 | -.37 (.15) | .02 | [-.67, -.07] | -2.39* | .02 | .66 |  |  |
| Thesis | 4 | .25 (.35) | .12 | [-.44, .93] | .70 | .49 | .66 |  |  |
| Patient Selection – Risk of Bias |  |  |  |  |  |  |  | 2.19 (2) | .34 |
| High | 12 | -.13 (.21) | .04 | [-.54, .28] | -.62 | .53 | .68 |  |  |
| Low | 1 | .35 (.70) | .49 | [-1.01, 1.72] | .51 | .61 | .68 |  |  |
| Unclear | 12 | -.47 (.21) | .05 | [-.89, -.06] | -2.24* | .03 | .68 |  |  |
| Patient Selection - Applicability |  |  |  |  |  |  |  | .52 (2) | .77 |
| High | 4 | -.06 (.35) | .12 | [-.75, .62] | -.18 | .86 | .68 |  |  |
| Low | 20 | -.32 (.16) | .02 | [-.64, -.01] | -2.00 | .05 | .68 |  |  |
| Unclear | 1 | -.10 (.72) | .52 | [-1.51, 1.31] | -.14 | .89 | .68 |  |  |
| Reference Standard – Risk of Bias |  |  |  |  |  |  |  | 1.60 (2) | .45 |
| High | 1 | .54 (.74) | .55 | [-.91, 2.00] | .73 | .46 | .67 |  |  |
| Low | 16 | -.24 (.18) | .03 | [-.59, .11] | -1.33 | .18 | .67 |  |  |
| Unclear | 8 | -.42 (.25) | .06 | [-.91, .07] | -1.69 | .09 | .67 |  |  |
| Flow and Timing – Risk of Bias |  |  |  |  |  |  |  | 6.74 (2) | .03 |
| High | 5 | -.98 (.32) | .10 | [-1.60, -.36] | -3.09** | .00 | .41 |  |  |
| Low | 10 | .01 (.21) | .05 | [-.41, .43] | .03 | .97 | .41 |  |  |
| Unclear | 10 | -.22 (.21) | .05 | [-.64, .20] | -1.01 | .31 | .41 |  |  |
| **FCC** | | | | | | | | | |
| Test Version |  |  |  |  |  |  |  | .41 (1) | .52 |
| Desktop (V1) | 9 | -.54 (.20) | .04 | [-.93, -.15] | -2.75* | .01 | .55 |  |  |
| iPad (V2) | 16 | -.69 (.15) | .02 | [-.98, -.41] | -4.83** | .00 | .55 |  |  |
| Article Type |  |  |  |  |  |  |  | 2.05 (1) | .15 |
| Peer-Reviewed | 27 | -.65 (.12) | .01 | [-.87, -.42] | -5.57** | .00 | .56 |  |  |
| Thesis | 6 | -.26 (.25) | .06 | [-.74, .23] | -1.03 | .30 | .56 |  |  |
| Patient Selection – Risk of Bias |  |  |  |  |  |  |  | .46 (2) | .80 |
| High | 13 | -.56 (.18) | .03 | [-.90, -.21] | -3.16** | .00 | .59 |  |  |
| Low | 6 | -.45 (.26) | .07 | [-.95, .05] | -1.77 | .08 | .59 |  |  |
| Unclear | 14 | -.66 (.17) | .03 | [-.99, -.32] | -3.85** | .00 | .59 |  |  |
| Patient Selection - Applicability |  |  |  |  |  |  |  | .15 (2) | .93 |
| High | 4 | -.48 (.30) | .09 | [-1.07, .11] | -1.60 | .11 | .57 |  |  |
| Low | 28 | -.59 (.12) | .01 | [-.82, -.37] | -5.11** | .00 | .57 |  |  |
| Unclear | 1 | -.50 (.62) | .39 | [-1.72, .72] | -.80 | .42 | .57 |  |  |
| Reference Standard – Risk of Bias |  |  |  |  |  |  |  | 1.24 (2) | .54 |
| High | 1 | .08 (.65) | .42 | [-1.19, 1.34] | .12 | .90 | .56 |  |  |
| Low | 22 | -.56 (.13) | .02 | [-.82, -.31] | -4.40** | .00 | .56 |  |  |
| Unclear | 10 | -.66 (.18) | .04 | [-1.40, -.29] | -3.44* | .00 | .56 |  |  |
| Flow and Timing – Risk of Bias |  |  |  |  |  |  |  | 10.49 (2)* | .01 |
| High | 10 | -1.70 (.18) | .03 | [-1.43, -.71] | -5.84** | .00 | .53 |  |  |
| Low | 9 | -.33 (.19) | .04 | [-.70, .03] | -1.78 | .07 | .53 |  |  |
| Unclear | 14 | -.39 (.15) | .02 | [-.69, -.08] | -2.50* | .01 | .53 |  |  |
| **CCC** | | | | | | | | | |
| Test Version |  |  |  |  |  |  |  | .26 (1) | .60 |
| Desktop (V1) | 6 | .39 (.35) | .13 | [-.31, 1.08] | 1.03 | .30 | .90 |  |  |
| iPad (V2) | 12 | .16 (.23) | .05 | [-.30, .61] | .62 | .53 | .90 |  |  |
| Article Type |  |  |  |  |  |  |  | .33 (1) | .57 |
| Peer-Reviewed | 20 | .23 (.19) | .04 | [-.14, .61] | 1.22 | .22 | .83 |  |  |
| Thesis | 6 | .46 (.35) | .12 | [-.23, 1.15] | 1.32 | .19 | .83 |  |  |
| Patient Selection – Risk of Bias |  |  |  |  |  |  |  | 1.19 (2) | .55 |
| High | 13 | .44 (.23) | .06 | [-.02, .90] | 1.89 | .06 | .81 |  |  |
| Low | 2 | .44 (.59) | .34 | [-.71, 1.59] | .75 | .46 | .81 |  |  |
| Unclear | 11 | .08 (.25) | .06 | [-.42, .57] | .30 | .76 | .81 |  |  |
| Patient Selection - Applicability |  |  |  |  |  |  |  | .73 (2) | .69 |
| High | 4 | .62 (.42) | .18 | [-.21, 1.45] | 1.46 | .14 | .83 |  |  |
| Low | 21 | .22 (.19) | .04 | [-.15, .59] | 1.18 | .24 | .83 |  |  |
| Unclear | 1 | .30 (.86) | .74 | [-1.39, 1.99] | .35 | .73 | .83 |  |  |
| Reference Standard – Risk of Bias |  |  |  |  |  |  |  | 1.17 (2) | .56 |
| High | 1 | .88 (.87) | .76 | [-.82, 2.59] | 1.01 | .31 | .81 |  |  |
| Low | 16 | .37 (.21) | .04 | [-.04, .78] | 1.76 | .08 | .81 |  |  |
| Unclear | 9 | .08 (.28) | .08 | [-.47, .63] | .28 | .78 | .81 |  |  |
| Flow and Timing – Risk of Bias |  |  |  |  |  |  |  | 6.98 (2)* | .03 |
| High | 8 | -.31 (.28) | .08 | [-.86, .23] | -1.12 | .26 | .75 |  |  |
| Low | 7 | .69 (.29) | .09 | [.11, 1.26] | 2.33* | .02 | .75 |  |  |
| Unclear | 11 | .46 (.24) | .06 | [.00, .93] | 1.95 | .05 | .75 |  |  |
| **FICA** | | | | | | | | | |
| Test Version |  |  |  |  |  |  |  | 10.25 (1)** | .00 |
| Desktop (V1) | 17 | -.50 (.13) | .02 | [-.75, -.24] | -3.90** | .00 | .48 |  |  |
| iPad (V2) | 35 | -.99 (.09) | .01 | [-1.15, -.82] | -11.73** | .00 | .48 |  |  |
| Article Type |  |  |  |  |  |  |  | 4.61 (1)* | .03 |
| Peer-Reviewed | 58 | -.86 (.07) | .01 | [-1.00, -.72] | -12.02** | .00 | .51 |  |  |
| Thesis | 12 | -.49 (.16) | .03 | [-.80, -.18] | -3.08** | .00 | .51 |  |  |
| Patient Selection – Risk of Bias |  |  |  |  |  |  |  | .04 (2) | .98 |
| High | 36 | -.79 (.10) | .01 | [-.98, -.60] | -8.19** | .00 | .54 |  |  |
| Low | 8 | -.79 (.20) | .04 | [-1.19, -.39] | -3.90** | .00 | .54 |  |  |
| Unclear | 26 | -.82 (.11) | .01 | [-1.04, -.60] | -7.18** | .00 | .54 |  |  |
| Patient Selection - Applicability |  |  |  |  |  |  |  | .00 (2) | 1.00 |
| High | 10 | -.80 (.18) | .03 | [-1.15, -.45] | -4.48** | .00 | .53 |  |  |
| Low | 58 | -.80 (.08) | .01 | [-.95, -.65] | -10.61** | .00 | .53 |  |  |
| Unclear | 2 | -.80 (.40) | .16 | [-1.59, -.01] | -1.99* | .047 | .53 |  |  |
| Reference Standard – Risk of Bias |  |  |  |  |  |  |  | .56 (2) | .76 |
| High | 1 | -.74 (.62) | .38 | [-1.95, .47] | -1.19 | .23 | .53 |  |  |
| Low | 47 | -.84 (.08) | .01 | [-1.00, -.67] | -10.09** | .00 | .53 |  |  |
| Unclear | 22 | -.73 (.12) | .02 | [-.96, -.49] | -6.00** | .00 | .53 |  |  |
| Flow and Timing – Risk of Bias |  |  |  |  |  |  |  | 4.42 (2) | .11 |
| High | 18 | -1.02 (.14) | .02 | [-1.29, -.76] | -7.56** | .00 | .53 |  |  |
| Low | 28 | -.66 (.11) | .01 | [-.87, -.45] | -6.16** | .00 | .53 |  |  |
| Unclear | 24 | -.80 (.12) | .01 | [-1.03, -.57] | -6.85** | .00 | .53 |  |  |
| **DCCS** | | | | | | | | | |
| Test Version |  |  |  |  |  |  |  | .04 (1) | .85 |
| Desktop (V1) | 18 | -.41 (.15) | .02 | [-.71, -.12] | -2.66* | .01 | .62 |  |  |
| iPad (V2) | 34 | -.45 (.11) | .01 | [-.66, -.24] | -4.14** | .00 | .62 |  |  |
| Article Type |  |  |  |  |  |  |  | .04 (1) | .84 |
| Peer-Reviewed | 56 | -.37 (.08) | .01 | [-.53, -.21] | -4.50** | .00 | .59 |  |  |
| Thesis | 10 | -.33 (.20) | .04 | [-.72, .06] | -1.65 | .10 | .59 |  |  |
| Patient Selection – Risk of Bias |  |  |  |  |  |  |  | .90 (2) | .64 |
| High | 32 | -.43 (.11) | .01 | [-.65, -.20] | -3.76** | .00 | .61 |  |  |
| Low | 9 | -.42 (.21) | .05 | [-.84, -.01] | -1.99* | .047 | .61 |  |  |
| Unclear | 25 | -.27 (.13) | .02 | [-.52, -.02] | -2.10* | .04 | .61 |  |  |
| Patient Selection - Applicability |  |  |  |  |  |  |  | 1.81 (2) | .41 |
| High | 11 | -.45 (.19) | .04 | [-.82, -.08] | -2.40* | .02 | .59 |  |  |
| Low | 53 | -.37 (.09) | .01 | [-.54, -.20] | -4.28** | .00 | .59 |  |  |
| Unclear | 2 | .19 (.44) | .20 | [-.67, 1.06] | .44 | .66 | .59 |  |  |
| Reference Standard – Risk of Bias |  |  |  |  |  |  |  | 3.57 (2) | .17 |
| High | 1 | .59 (.67) | .44 | [-.72, 1.89] | .88 | .38 | .58 |  |  |
| Low | 45 | -.44 (.09) | .01 | [-.62, -.26] | -4.79** | .00 | .58 |  |  |
| Unclear | 20 | -.24 (.14) | .02 | [-.51, .03] | -1.71 | .09 | .58 |  |  |
| Flow and Timing – Risk of Bias |  |  |  |  |  |  |  | 3.56 (2) | .17 |
| High | 16 | -.59 (.16) | .03 | [-.90, -.28] | -3.69** | .00 | .59 |  |  |
| Low | 27 | -.38 (.12) | .02 | [-.62, -.15] | -3.18** | .00 | .59 |  |  |
| Unclear | 23 | -.20 (.08) | .02 | [-.46, .06] | -1.51 | .13 | .59 |  |  |
| **LSWM** | | | | | | | | | |
| Test Version |  |  |  |  |  |  |  | .24 (1) | .63 |
| Desktop (V1) | 17 | -.24 (.12) | .02 | [-.48, -.01] | -1.97* | .049 | .46 |  |  |
| iPad (V2) | 29 | -.35 (.09) | .01 | [-.49, -.15] | -3.57** | .00 | .46 |  |  |
| Article Type |  |  |  |  |  |  |  | .75 (1) | .39 |
| Peer-Reviewed | 54 | -.23 (.07) | .00 | [-.36, -.10] | -3.42** | .00 | .44 |  |  |
| Thesis | 9 | -.32 (.17) | .03 | [-.65, .00] | -1.94 | .05 | .44 |  |  |
| Patient Selection – Risk of Bias |  |  |  |  |  |  |  | 2.11 (2) | .35 |
| High | 33 | -.16 (.08) | .01 | [-.32, .01] | -1.85 | .07 | .44 |  |  |
| Low | 7 | -.30 (.18) | .03 | [-.66, .05] | -1.68 | .09 | .44 |  |  |
| Unclear | 23 | -.34 (.10) | .01 | [-.54, -.14] | -3.35** | .00 | .44 |  |  |
| Patient Selection - Applicability |  |  |  |  |  |  |  | .30 (1) | .86 |
| High | 10 | -.32 (.15) | .02 | [-.62, -.02] | -2.06* | .04 | .45 |  |  |
| Low | 52 | -.23 (.07) | .01 | [-.36, -.09] | -3.30** | .00 | .45 |  |  |
| Unclear | 1 | -.20 (.51) | .26 | [-1.21, .81] | -.39 | .70 | .45 |  |  |
| Reference Standard – Risk of Bias |  |  |  |  |  |  |  | .03 (2) | .99 |
| High | 1 | -.32 (.55) | .30 | [-1.40, .75] | -.59 | .56 | .45 |  |  |
| Low | 43 | -.24 (.08) | .01 | [-.39, -.10] | -3.24* | .00 | .45 |  |  |
| Unclear | 19 | -.23 (.11) | .01 | [-.45, -.01] | -2.03* | .04 | .45 |  |  |
| Flow and Timing – Risk of Bias |  |  |  |  |  |  |  | 8.29 (2)* | .02 |
| High | 14 | -.56 (.13) | .02 | [-.81, -.31] | -4.42** | .00 | .43 |  |  |
| Low | 26 | -.13 (.09) | .01 | [-.31, .05] | -1.43 | .15 | .43 |  |  |
| Unclear | 23 | -.17 (.10) | .01 | [-.36, .03] | -1.67 | .10 | .43 |  |  |
| **PCPS** | | | | | | | | | |
| Test Version |  |  |  |  |  |  |  |  |  |
| Desktop (V1) | 17 | -.77 (.19) | .03 | [-1.14, -.41] | -4.17** | .00 | .73 | .01 (1) | .90 |
| iPad (V2) | 28 | -.74 (.14) | .02 | [-1.01, -.48] | -5.41** | .00 | .73 |  |  |
| Article Type |  |  |  |  |  |  |  | .02 (1) | .88 |
| Peer-Reviewed | 56 | -.74 (.10) | .01 | [-.92, -.55] | -7.73** | .00 | .68 |  |  |
| Thesis | 7 | -.69 (.27) | .07 | [-1.22, -.16] | -2.55* | .01 | .68 |  |  |
| Patient Selection – Risk of Bias |  |  |  |  |  |  |  | 3.38 (2) | .19 |
| High | 32 | -.61 (.12) | .02 | [-.84, -.37] | -4.97** | .00 | .66 |  |  |
| Low | 8 | -.63 (.24) | .06 | [-1.10, -.15] | -2.58* | .01 | .66 |  |  |
| Unclear | 23 | -.94 (.15) | .02 | [-1.23, -.66] | -6.51** | .00 | .66 |  |  |
| Patient Selection - Applicability |  |  |  |  |  |  |  | .63 (2) | .73 |
| High | 10 | -.66 (.22) | .05 | [-1.10, -.22] | -2.95** | .00 | .68 |  |  |
| Low | 51 | -.73 (.10) | .01 | [-.93, -.53] | -7.29** | .00 | .68 |  |  |
| Unclear | 2 | -1.10 (.50) | .25 | [-2.08, -.11] | -2.19* | .03 | .68 |  |  |
| Reference Standard – Risk of Bias |  |  |  |  |  |  |  |  |  |
| High | 1 | -.10 (.75) | .57 | [-1.58, 1.38] | -.13 | .90 | .68 |  |  |
| Low | 43 | -.69 (.11) | .01 | [-.90, -.48] | -6.35** | .00 | .68 |  |  |
| Unclear | 19 | -.85 (.17) | .03 | [-1.18, -.53] | -5.17** | .00 | .68 |  |  |
| Flow and Timing – Risk of Bias |  |  |  |  |  |  |  | 5.12 (2) | .08 |
| High | 14 | -1.12 (.20) | .04 | [-1.50, -.74] | -5.74** | .00 | .69 |  |  |
| Low | 26 | -.63 (.14) | .02 | [-.91, -.35] | -4.45** | .00 | .69 |  |  |
| Unclear | 23 | -.61 (.15) | .02 | [-.91, -.31] | -4.01** | .00 | .69 |  |  |
| **PSM** | | | | | | | | | |
| Test Version |  |  |  |  |  |  |  | .95 (1) | .33 |
| Desktop (V1) | 14 | -.29 (.12) | .01 | [-.53, -.06] | -2.44* | .02 | .39 |  |  |
| iPad (V2) | 26 | -.15 (.08) | .01 | [-.31, .01] | -1.82 | .07 | .39 |  |  |
| Article Type |  |  |  |  |  |  |  | .05 (1) | .83 |
| Peer-Reviewed | 46 | -.14 (.07) | .00 | [-.27, -.01] | -2.14* | .03 | .40 |  |  |
| Thesis | 8 | -.10 (.16) | .03 | [-.42, .22] | -.64 | .53 | .40 |  |  |
| Patient Selection – Risk of Bias |  |  |  |  |  |  |  | 3.43 (2) | .18 |
| High | 29 | -.03 (.08) | .01 | [-.19, .13] | -.38 | .70 | .39 |  |  |
| Low | 6 | -.26 (.18) | .03 | [-.62, .09] | -1.46 | .14 | .39 |  |  |
| Unclear | 19 | -.25 (.10) | .01 | [-.45, -.05] | -2.48* | .01 | .39 |  |  |
| Patient Selection - Applicability |  |  |  |  |  |  |  | .76 (2) | .68 |
| High | 7 | -.19 (.17) | .03 | [-.52, .14] | -1.14 | .26 | .40 |  |  |
| Low | 45 | -.12 (.07) | .00 | [-.25, .02] | -1.72 | .09 | .40 |  |  |
| Unclear | 2 | -.37 (.31) | .10 | [-.98, .25] | -1.18 | .24 | .40 |  |  |
| Reference Standard – Risk of Bias |  |  |  |  |  |  |  | .81 (2) | .67 |
| High | 1 | .36 (.51) | .26 | [-.73, 1.25] | .51 | .61 | .39 |  |  |
| Low | 35 | -.12 (.07) | .01 | [-.27, .03] | -1.62 | .10 | .39 |  |  |
| Unclear | 18 | -.18 (.11) | .01 | [-.39, .03] | -1.70 | .09 | .39 |  |  |
| Flow and Timing – Risk of Bias |  |  |  |  |  |  |  | 2.04 (2) | .36 |
| High | 10 | -.31 (.14) | .02 | [-.59, -.04] | -2.25* | .03 | .38 |  |  |
| Low | 25 | -.09 (.09) | .01 | [-.26, .08] | -1.00 | .32 | .38 |  |  |
| Unclear | 19 | -.11 (.10) | .01 | [-.30, .09] | -1.05 | .30 | .38 |  |  |
| **ORR** | | | | | | | | | |
| Test Version |  |  |  |  |  |  |  | .44 (1) | .51 |
| Desktop (V1) | 9 | .47 (.17) | .03 | [.14, .80] | 2.81* | .01 | .45 |  |  |
| iPad (V2) | 14 | .33 (.12) | .02 | [.09, .58] | 2.71* | .01 | .45 |  |  |
| Article Type |  |  |  |  |  |  |  | .04 (1) | .85 |
| Peer-Reviewed | 21 | .39 (.11) | .01 | [.18, .60] | 3.69** | .00 | .44 |  |  |
| Thesis | 4 | .34 (.25) | .06 | [-.15, .83] | 1.34 | .18 | .44 |  |  |
| Patient Selection – Risk of Bias |  |  |  |  |  |  |  | 1.36 (2) | .51 |
| High | 20 | .44 (.11) | .01 | [.21, .66] | 3.83** | .00 | .47 |  |  |
| Low | 1 | 1.01 (.49) | .24 | [.05, 1.96] | 2.06* | .03 | .47 |  |  |
| Unclear | 10 | .42 (.17) | .03 | [.09, .74] | 2.52* | .01 | .47 |  |  |
| Patient Selection - Applicability |  |  |  |  |  |  |  | .34 (2) | .85 |
| High | 5 | .33 (.23) | .05 | [-.13, .78] | 1.41 | .16 | .48 |  |  |
| Low | 25 | .47 (.11) | .01 | [.27, .68] | 4.52** | .00 | .48 |  |  |
| Unclear | 1 | .50 (.54) | .29 | [-.56, 1.56] | .92 | .36 | .48 |  |  |
| Reference Standard – Risk of Bias |  |  |  |  |  |  |  | .59 (2) | .75 |
| High | 1 | .63 (.57) | .32 | [-.49, 1.74] | 1.11 | .27 | .47 |  |  |
| Low | 21 | .40 (.11) | .01 | [.18, .62] | 3.59** | .00 | .47 |  |  |
| Unclear | 9 | .55 (.17) | .03 | [.21, .88] | 3.18* | .00 | .47 |  |  |
| Flow and Timing – Risk of Bias |  |  |  |  |  |  |  | 2.46 (2) | .29 |
| High | 7 | .26 (.21) | .04 | [-.14, .66] | 1.26 | .21 | .48 |  |  |
| Low | 13 | .61 (.14) | .02 | [.33, .90] | 4.27** | .00 | .48 |  |  |
| Unclear | 11 | .37 (.16) | .03 | [.05, .68] | 2.29* | .05 | .48 |  |  |
| **PV** | | | | | | | | | |
| Test Version |  |  |  |  |  |  |  | 1.50 (1) | .22 |
| Desktop (V1) | 10 | .41 (.15) | .02 | [.11, .71] | 2.65* | .01 | .43 |  |  |
| iPad (V2) | 16 | .17 (.11) | .01 | [-.05, .40] | 1.54 | .12 | .43 |  |  |
| Article Type |  |  |  |  |  |  |  | .13 (1) | .72 |
| Peer-Reviewed | 24 | .27 (.10) | .01 | [.08, .46] | 2.78* | .01 | .43 |  |  |
| Thesis | 4 | .18 (.24) | .06 | [-.30, .65] | .72 | .47 | .43 |  |  |
| Patient Selection – Risk of Bias |  |  |  |  |  |  |  | 3.14 (2) | .21 |
| High | 21 | .33 (.10) | .01 | [.14, .53] | 3.41** | .00 | .40 |  |  |
| Low | 3 | -.08 (.25) | .06 | [-.57, .41] | -.32 | .75 | .40 |  |  |
| Unclear | 14 | .15 (.12) | .02 | [-.09, .39] | 1.20 | .23 | .40 |  |  |
| Patient Selection - Applicability |  |  |  |  |  |  |  | .04 (2) | .98 |
| High | 5 | .21 (.20) | .04 | [-.19, .61] | 1.05 | .30 | .40 |  |  |
| Low | 31 | .24 (.08) | .01 | [.08, .40] | 2.89** | .00 | .40 |  |  |
| Unclear | 2 | .18 (.32) | .10 | [-.44, .80] | .58 | .57 | .40 |  |  |
| Reference Standard – Risk of Bias |  |  |  |  |  |  |  | 1.70 (2) | .43 |
| High | 1 | .85 (.51) | .26 | [-.15, 1.84] | 1.67 | .10 | .40 |  |  |
| Low | 23 | .24 (.09) | .01 | [.06, .43] | 2.60* | .01 | .40 |  |  |
| Unclear | 14 | .18 (.12) | .01 | [-.06, .41] | 1.48 | .14 | .40 |  |  |
| Flow and Timing – Risk of Bias |  |  |  |  |  |  |  | 7.70 (2)* | .02 |
| High | 9 | -.10 (.15) | .02 | [-.38, .19] | -.65 | .51 | .38 |  |  |
| Low | 14 | .42 (.11) | .01 | [.20, .64] | 3.70** | .00 | .38 |  |  |
| Unclear | 15 | .24 (.11) | .01 | [.02, .46] | 2.09* | .04 | .38 |  |  |

*Note.* SE = standard error; V1 = Version 1; V2 = Version 2; FCC = Fluid Cognition Composite, CCC = Crystallized Cognition Composite, CFC = Cognitive Function Composite, FICA = Flanker Inhibitory Control Attention Test, DCCS = Dimensional Change Card Sort Test, LSWM = List Sort Working Memory Test, PCPS = Pattern Comparison Processing Speed Test, PSM = Picture Sequence Memory Test, ORR = Oral Reading Recognition Test, PV = Picture Vocabulary Test, **p* < .05, *** p* < .01

**Supplemental Figure 1.** *Proportion of studies rated as high, low, and unclear risk of bias.*


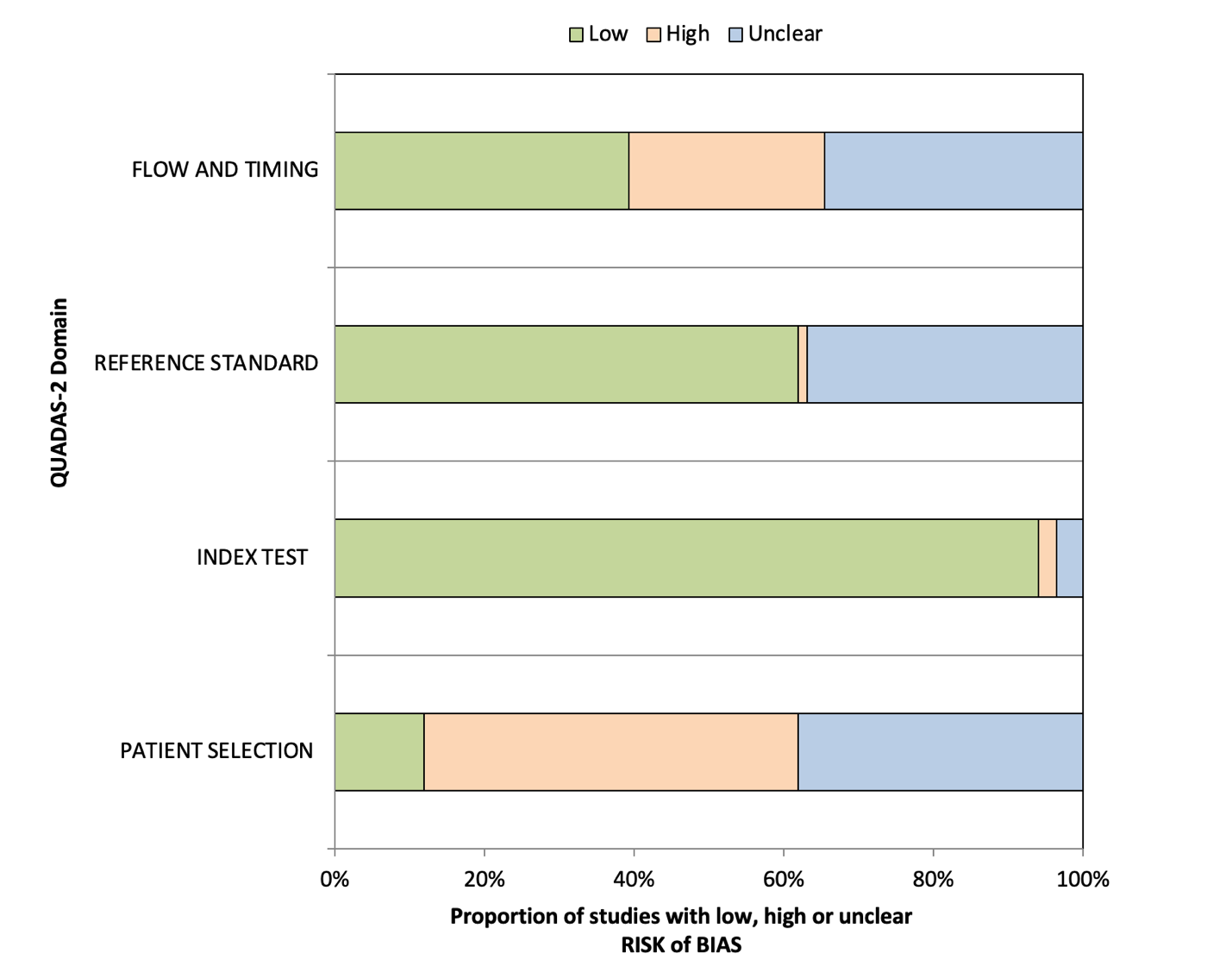


**Supplemental Figure 2.** *Proportion of studies rated as high, low, and unclear concerns regarding applicability.*


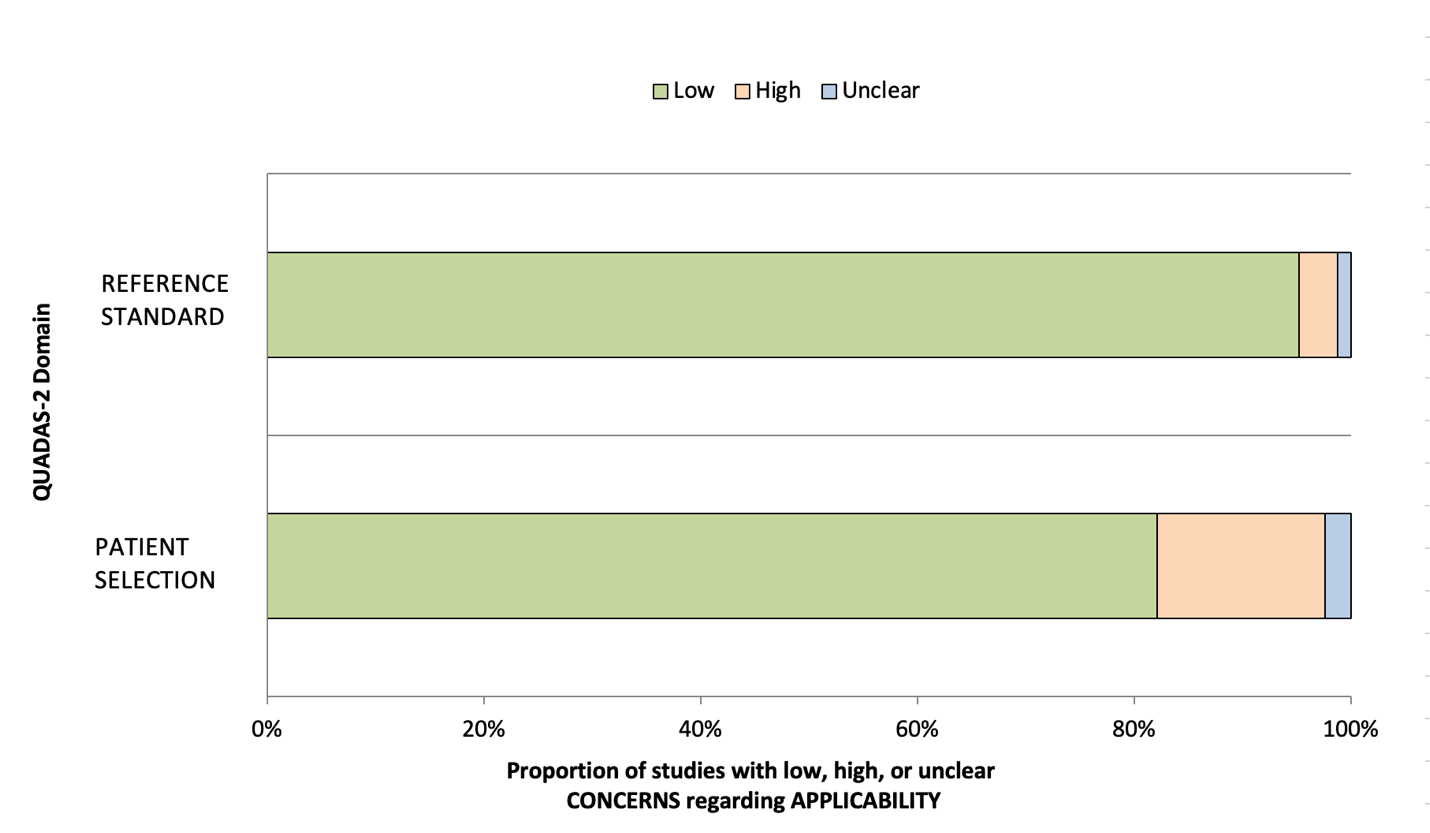

Supplement: Supplementary file 1 — (DOCX 254 KB) [file 11065_2025_9669_MOESM1_ESM.docx]
